# Excess Deaths of Gastrointestinal, Liver, and Pancreatic Diseases During the COVID-19 Pandemic in the United States

**DOI:** 10.3389/ijph.2023.1606305

**Published:** 2023-08-15

**Authors:** Lefei Han, Haoting Shi, Yongxuan Li, Hongchao Qi, Yuhua Wang, Jiawei Gu, Jiayin Wu, Shi Zhao, Peihua Cao, Lan Xu, Xiaobei Deng, Xiaoxin I. Yao, Jinjun Ran

**Affiliations:** ^1^ School of Global Health, Chinese Center for Tropical Diseases Research, Shanghai Jiao Tong University School of Medicine, Shanghai, China; ^2^ Ruijin Hospital, Shanghai Jiao Tong University School of Medicine, Shanghai, China; ^3^ School of Public Health, Shanghai Jiao Tong University School of Medicine, Shanghai, China; ^4^ British Heart Foundation Cardiovascular Epidemiology Unit, Department of Public Health and Primary Care, University of Cambridge, Cambridge, United Kingdom; ^5^ JC School of Public Health and Primary Care, Chinese University of Hong Kong, Hong Kong, Hong Kong SAR, China; ^6^ Clinical Research Center, Zhujiang Hospital, Southern Medical University, Guangzhou, China; ^7^ Department of Clinical Research, The Eighth Affiliated Hospital, Sun Yat-sen University, Shenzhen, China

**Keywords:** excess mortality, GI hemorrhage, liver disorder, disparity, COVID-19

## Abstract

**Objectives:** To evaluate excess deaths of gastrointestinal, liver, and pancreatic diseases in the United States during the COVID-19 pandemic.

**Methods:** We retrieved weekly death counts from National Vital Statistics System and fitted them with a quasi-Poisson regression model. Cause-specific excess deaths were calculated by the difference between observed and expected deaths with adjustment for temporal trend and seasonality. Demographic disparities and temporal-spatial patterns were evaluated for different diseases.

**Results:** From March 2020 to September 2022, the increased mortality (measured by excess risks) for *Clostridium difficile* colitis, gastrointestinal hemorrhage, and acute pancreatitis were 35.9%; 24.8%; and 20.6% higher than the expected. For alcoholic liver disease, fibrosis/cirrhosis, and hepatic failure, the excess risks were 1.4–2.8 times higher among younger inhabitants than older inhabitants. The excess deaths of selected diseases were persistently observed across multiple epidemic waves with fluctuating trends for gastrointestinal hemorrhage and fibrosis/cirrhosis and an increasing trend for *C. difficile* colitis.

**Conclusion:** The persistently observed excess deaths of digestive diseases highlights the importance for healthcare authorities to develop sustainable strategies in response to the long-term circulating of SARS-CoV-2 in the community.

## Introduction

Gastrointestinal (GI), liver, and pancreatic diseases pose a substantial healthcare burden in the United States (US), accounting for more than 54 million annual ambulatory visits and about 3 million hospital admissions before the COVID-19 pandemic [1]. During the pandemic, healthcare systems have suffered from heavy burdens in response to the emerging crisis and maintaining routine care services, which might have substantial additional deaths [2, 3]. The estimated excess all-cause mortality during the pandemic was about three times higher than the reported COVID-19 deaths [4]. The measurement quantified the overall mortality burden directly caused by COVID-19 infections and that indirectly caused by the pandemic [5]. Previous evidence has indicated patients with digestive-related abnormalities were vulnerable to the pandemic, not only because of their higher mortality risk of COVID-19, but their unmet healthcare needs due to the overwhelmed healthcare services [6]. However, few studies have quantified the impact of the pandemic on individuals with digestive diseases. There is also limited research examining the excess deaths associated with digestive diseases during the pandemic.

With an in-depth understanding of SARS-CoV-2 and a significant improvement in the healthcare capacity, COVID-19 has become manageable. Although the World Health Organization (WHO) announced that COVID-19 no longer has constitutes a public health emergency of international concern (PHEIC) since May 2023, it remains an ongoing health issue that requires long-term management and preparedness [7]. However, the long-term impact of COVID-19 on patients with underlying medical conditions warrants more evaluation. As the SARS-CoV-2 will continuously circulate in the community and bring a persistent impact to the public, it is important to assess the mortality burden over a longer period to comprehend the healthcare needs among patients with the digestive-related illness.

In this study, we estimated the excess deaths associated with selected subtype digestive diseases across the multiple waves of the COVID-19 pandemic in the United States. We also compared the difference of excess deaths by age, sex, race/ethnicity and evaluated temporal patterns and regional differences.

## Methods

### Data Sources

We retrieved the provisional death counts from January 2018 to November 2022 from the National Vital Statistics System through the Wide-ranging Online Data for the Epidemiologic Research database of the Centers for Disease Control and Prevention (CDC WONDER) [8]. The database is an integrated public health information and communication system, containing death data of more than 99% of decedents in the United States. The aggregated data were stratified by cause of death, age (20–64 years and 65–84 years), sex (male and female), race and ethnicity (non-Hispanic White, non-Hispanic Black and Hispanic), and state (50 states and District of Columbia). Considering a potential reporting delay of the death registry data, we excluded data after October 2022 to ensure the data completeness. We retrieved population data during 2018–2021 from the Census Bureau, and the data in 2022 were assumed stable with that in 2021 [9]. Since all the data were publicly available and personal information was de-identifiable, the institutional review board approval and informed consent were exempted.

The causes of death were ascertained according to the International Statistical Classification of Diseases and Related Health Problems, 10th Revision (ICD-10) stated on the death certificate. Both underlying and contributing causes of death that assigned to digestive diseases were considered in this study. The underlying cause of death was identified if a certain disease was listed as the primary cause of death on the death certificate. Since some digestive diseases could be one of chronic conditions for patients, the contributing causes of death were also considered if digestive diseases were listed as one of the multiple causes of death on the death certificate. We primarily reported results associated with contributing causes of death in the main text. The study selected the dominant causes of death relevant to the digestive system for analyses based on the previous reports [10], including GI hemorrhage (not otherwise specified, NOS), ulcers, paralytic ileus and intestinal obstruction, vascular disorders of the intestine, *Clostridium difficile* colitis (*C. difficile* colitis), esophageal cancer (EC), gastric cancer (GC), colorectal cancer (CRC), alcoholic liver disease (ALD), fibrosis/cirrhosis, chronic hepatitis C, hepatic failure, liver and intrahepatic bile duct cancer (LIHC), acute pancreatitis (AP), and pancreatic cancer (PC). The ICD-10 codes for each specific disease were listed in Supplementary Table S1.

Since the first death case of COVID-19 was reported in March 2020, the US has experienced several COVID-19 mortality waves as of September 2022. We identified the start and end dates of each wave according to the calendar date of a trough between two consecutive waves. The periods from Wave I to VI were: March 2020 to June 2020 (Wave I), June 2020 to October 2020 (Wave II), October 2020 to June 2021 (Wave III), June 2021 to November 2021 (Wave IV), November 2021 to May 2022 (Wave V), and June 2022 to September 2022 (Wave VI). Information on COVID-19 mortality waves in detail, including the number of weeks and the dominant variants in each wave, was shown in Supplementary Table S2.

### Statistical Analysis

To quantify excess deaths of the selected causes, we first estimated the expected deaths, counterfactually assuming there was no pandemic during the period. We fitted the weekly death counts of each specific cause of death from January 2018 to February 2020 by a quasi-Poisson time-series regression model to predict the expected death counts from March 2020 to September 2022. The dispersion estimates and corresponding *p*-values were shown in Supplementary Table S3. The regression model was adjusted for temporal trend and seasonality, where a linear term for the calendar years from 2018 to 2022 and a smoothing cubic spline function for epidemiological weeks from week 1 to week 52 was added to the model. To determine the number of knots per year for the smoothing cubic spline function, we calculated the quasi-Akaike information criterion (qAIC) scores of regression models, for each cause of interest with the number of knots ranging from 1 to 12. Overall, the spline with six knots offered the smallest sum of qAIC scores for the fifteen cause-specific models (Supplementary Table S4). We also fitted cause-specific models by age, sex, ethnic/race, state, and pandemic wave, respectively.

Excess deaths were measured by absolute counts and relative percentage change. We calculated excess death counts by expected death counts subtracted from observed death counts, excess mortality (per million persons) by excess death counts divided by population size, and excess risk (ER, %) by excess death counts divided by expected death counts. The statistical uncertainty of expected death counts and excess death metrics were assessed by 95% confidence interval (CI). The following strategies were operated to verify the robustness of results: 1) to ensure the modeling period contains equal seasonal effects, we removed the death counts in January and February 2018 (i.e., restricted from March 2018 to March 2020) and then estimated the excess deaths; 2) we adjusted the seasonal effect through harmonics with varying periodicities. The study followed the Strengthening the Reporting of Observational Studies in Epidemiology (STROBE) reporting guidelines. All the analyses, figures and tables were performed using **R** version 4.4.2. The package “excessmort” was used for processing all the excess estimates [11].

## Results

**FIGURE 1:**
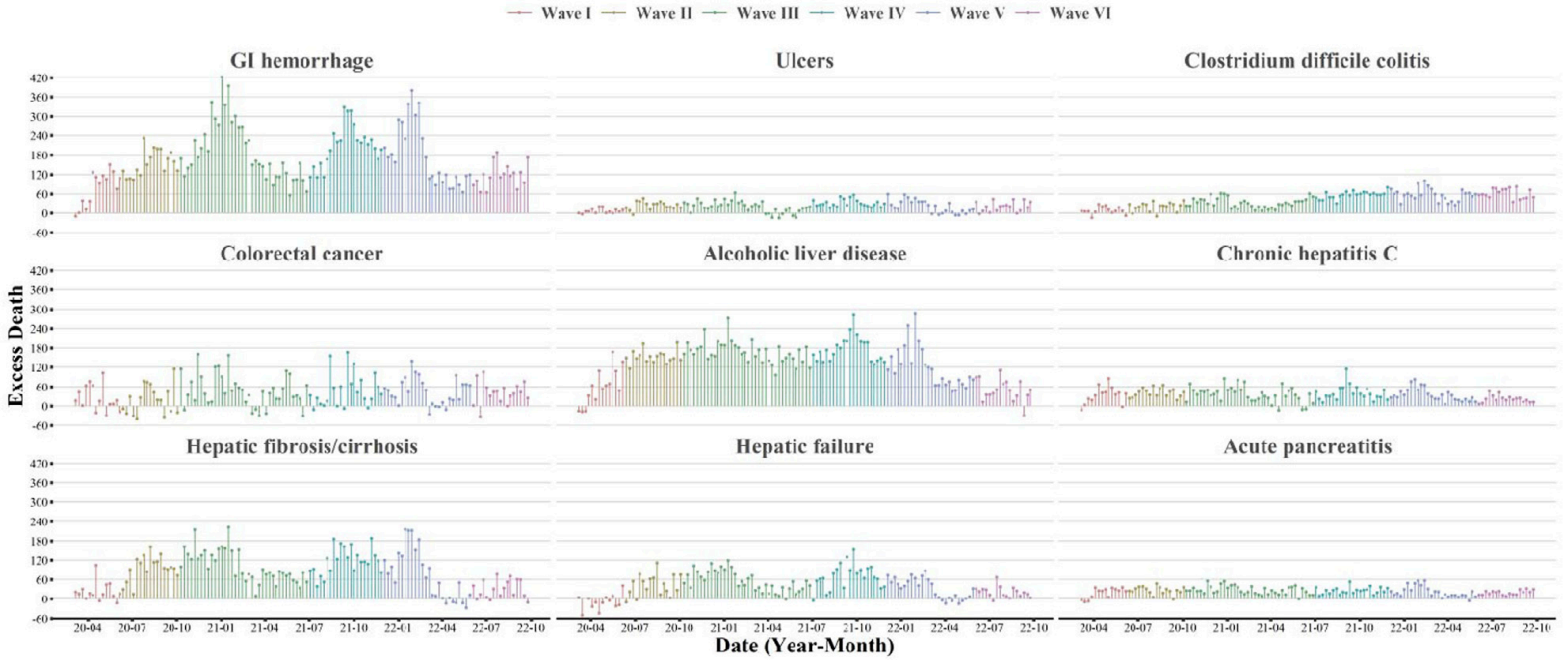
Weekly estimates of excess deaths associated with selected gastrointestinal, liver, and pancreatic diseases (United States, March 2020 to September 2022). This figure shows the time-series estimates of excess death counts associated with selected gastrointestinal, liver, and pancreatic diseases from March 2020 to September 2022. The contributing cause of death was adopted. The subtypes included: GI hemorrhage (not otherwise specified), ulcers, *Clostridium difficile* colitis, colorectal cancer, alcoholic liver disease, chronic hepatitis C, hepatic fibrosis/cirrhosis, hepatic failure, and acute pancreatitis. Waves were identified according to the weekly surveillance of COVID-19 deaths in the United States. Wave I was from March 2020 to June 2020, Wave II was from June 2020 to October 2020, Wave III was from October 2020 to June 2021, Wave IV was from June 2021 to November 2021, Wave V was from November 2021 to May 2022 and Wave VI was from May 2022 to September 2022.

During March 2020 and September 2022, the excess deaths of cause-specific digestive diseases have been persistently observed in the United States (Figure 1). The excess death counts were notable for GI hemorrhage (22,156, 95% CI, 21,478–22,504), ALD (17,531, 95% CI, 16,936–17,837), and fibrosis/cirrhosis (10,741, 95% CI, 9,930–11,157). Increased death risks for GI diseases were found largest for *C*. *difficile* colitis (ER 35.9%, 95% CI, 34.1%–37.8%), followed by GI hemorrhage (ER 24.8%, 95% CI, 24.0%–25.5%), ulcers (ER 15.1%, 95% CI, 13.6%–16.7%) and CRC (ER 3.4%, 95% CI, 2.9%–3.9%) (Table 1). For liver and pancreatic diseases, AP has the largest ER (20.6%, 95% CI, 18.9%–22.4%), followed by ALD (19.9%, 95% CI, 19.2%–20.6%), chronic hepatitis C (15.1%, 95% CI, 13.9%–16.3%), fibrosis/cirrhosis (8.5%, 95% CI, 7.9%–9.1%), and hepatic failure (7.1%, 95% CI, 6.4%–7.9%). The excess death estimates measured by the underlying causes of death were shown in Supplementary Table S5. The annual ERs for subtype diseases were shown in Supplementary Tables S6–S8.

**TABLE 1:** Excess mortality associated with digestive related diseases (United States, March 2020 to September 2022).

Cause of death^a^	Observed deaths, No.	Expected deaths No. (95% CI)	Excess deaths No. (95% CI)^b^	Excess mortality (95% CI)^c^	Excess risk % (95% CI)^d^
GI diseases					
GI hemorrhage, NOS	111,669	89,513 (88,835, 89,861)	22,156 (21,478, 22,504)	66.8 (64.7, 67.8)	24.8 (24.0, 25.5)
Ulcers	21,310	18,512 (18,230, 18,658)	2,798 (2,515, 2,944)	8.4 (7.6, 8.9)	15.1 (13.6, 16.7)
Paralytic ileus and intestinal obstruction	51,971	50,967 (50,463, 51,226)	1,004 (500, 1,263)	3.0 (1.5, 3.8)	2.0 (1.1, 2.8)
Vascular disorder of intestine	41,516	42,115 (41,653, 42,353)	−599 (−1,062, −362)	−1.8 (−3.2, −1.1)	−1.4 (−2.4, −0.5)
*C. difficile* colitis	20,533	15,108 (14,864, 15,234)	5,425 (5,182, 5,552)	16.3 (15.6, 16.7)	35.9 (34.1, 37.8)
EC	45,080	47,573 (47,118, 47,808)	−2,493 (−2,949, −2,259)	−7.5 (−8.9, −6.8)	−5.2 (−6.1, −4.4)
GC	31,249	32,044 (31,693, 32,225)	−795 (−1,146, −614)	−2.4 (−3.5, −1.8)	−2.5 (−3.6, −1.4)
CRC	162,973	157,576 (156,651, 158,049)	5,397 (4,473, 5,871)	16.3 (13.5, 17.7)	3.4 (2.9, 3.9)
Liver and pancreatic diseases					
ALD	105,612	88,081 (87,485, 88,386)	17,531 (16,936, 17,837)	52.8 (51.0, 53.7)	19.9 (19.2, 20.6)
Fibrosis/cirrhosis	136,997	126,256 (125,445, 126,672)	10,741 (9,930, 11,157)	32.4 (29.9, 33.6)	8.5 (7.9, 9.1)
Chronic hepatitis C	35,540	30,869 (30,509, 31,054)	4,671 (4,312, 4,857)	14.1 (13.0, 14.6)	15.1 (13.9, 16.3)
Hepatic failure	79,265	73,991 (73,409, 74,290)	5,274 (4,692, 5,573)	15.9 (14.1, 16.8)	7.1 (6.4, 7.9)
LIHC	82,556	82,160 (81,530, 82,483)	396 (−234, 719)	1.2 (−0.7, 2.2)	0.5 (−0.2, 1.2)
AP	18,297	15,170 (14,916, 15,302)	3,127 (2,873, 3,258)	9.4 (8.7, 9.8)	20.6 (18.9, 22.4)
PC	129,727	132,454 (131,611, 132,886)	−2,727 (−3,570, −2,295)	−8.2 (−10.8, −6.9)	−2.1 (−2.6, −1.5)

Abbreviations: CI, confidence interval; GI, gastrointestinal; NOS, not otherwise specified; *C. difficile*, *Clostridium difficile*; EC, esophageal cancer; GC, gastric cancer; CRC, colorectal cancer; ALD, alcohol liver disease; LIHC, liver and intrahepatic cancer; AP, acute pancreatitis; PC, pancreatic cancer.

^a^ The contributing cause of death was adopted.

^b^ Excess death number estimated by subtract the expected number from the observed number of death.

^c^ Excess mortality per 1,000,000 persons was estimated via the excess death number divided by population size.

^d^ Excess risk was calculated as the ratio of the excess-to-expected number of death.

The increased GI-disease related deaths were disproportionally distributed by age and race/ethnicity, but not by sex (Table 2 and Supplementary Tables S9–S11). Although the elderly population accounted for more excess death counts, younger population tended to have higher ERs of GI diseases, particularly for GI hemorrhage (38.7% in adults aged 20–64 years vs. 26.2% in adults aged 65–84 years), ulcers (23.4% vs. 9.8%), and *C*. *difficile* colitis (54.7% vs. 39.6%). Disproportional ERs were also observed in the stratification of race and ethnicity. Compared with non-Hispanic Whites, there was a 2.5 times higher ER of GI hemorrhage among Hispanics and a 1.7 times higher ER of ulcers among non-Hispanic Blacks. Also, the ER of *C. difficile* colitis was 49.2% (95% CI, 42.8%–55.7%) among the non-Hispanic Blacks, −0.2% (95% CI, −5.5%–5.4%) among the Hispanic inhabitants, and 37.2% (95% CI, 35.1%–39.4%) among the non-Hispanic Whites. A mild disparity was found between males and females for ERs of GI diseases.

**TABLE 2:** Excess mortality associated with selected gastrointestinal diseases by demographic factor and pandemic wave (United States, March 2020 to September 2022).

Cause of death^a^	Observed deaths, No.	Expected deaths No. (95% CI)	Excess deaths No. (95% CI)^b^	Excess mortality (95% CI)^c^	Excess risk % (95% CI)^d^
GI hemorrhage, NOS					
Age					
20–64 years	29,214	21,065 (20,720, 21,243)	8,149 (7,804, 8,327)	42.0 (40.3, 43.0)	38.7 (37.1, 40.3)
65–84 years	51,555	40,862 (40,443, 41,077)	10,693 (10,275, 10,909)	229.5 (220.5, 234.2)	26.2 (25.1, 27.3)
Sex					
Female	49,701	39,857 (39,422, 40,080)	9,844 (9,410, 10,068)	59.1 (56.5, 60.5)	24.7 (23.6, 25.8)
Male	61,968	49,302 (48,782, 49,569)	12,666 (12,147, 12,933)	77.6 (74.4, 79.2)	25.7 (24.7, 26.7)
Race/ethnicity^e^					
NHW	81,314	66,962 (66,360, 67,272)	14,352 (13,749, 14,661)	73.2 (70.1, 74.8)	21.4 (20.6, 22.3)
NHB	13,875	10,525 (10,292, 10,645)	3,350 (3,118, 3,471)	83.3 (77.6, 86.4)	31.8 (29.6, 34.0)
Hispanics	10,322	6,760 (6,599, 6,844)	3,562 (3,401, 3,646)	58.6 (55.9, 60.0)	52.7 (49.8, 55.7)
Wave^f^					
Wave I	10,532	9,439 (9,219, 9,554)	1,093 (873, 1,207)	3.3 (2.6, 3.6)	11.6 (9.5, 13.7)
Wave II	13,288	10,655 (10,421, 10,776)	2,633 (2,399, 2,754)	7.9 (7.2, 8.3)	24.7 (22.6, 26.8)
Wave III	33,477	26,215 (25,848, 26,404)	7,262 (6,895, 7,451)	21.9 (20.8, 22.4)	27.7 (26.3, 29.1)
Wave IV	18,776	14,177 (13,907, 14,317)	4,599 (4,329, 4,738)	13.9 (13.0, 14.3)	32.4 (30.6, 34.3)
Wave V	22,879	18,299 (17,993, 18,458)	4,580 (4,273, 4,738)	13.8 (12.9, 14.3)	25.0 (23.4, 26.7)
Wave VI	12,717	10,727 (10,492, 10,848)	1,990 (1,756, 2,112)	6.0 (5.3, 6.4)	18.6 (16.5, 20.6)
Ulcers					
Age					
20–64 years	3,156	2,557 (2,396, 2,641)	599 (438, 683)	3.1 (2.3, 3.5)	23.4 (19.2, 27.8)
65–84 years	10,705	9,752 (9,447, 9,910)	953 (647, 1,111)	20.5 (13.9, 23.8)	9.8 (7.7, 11.9)
Sex					
Female	9,976	8,918 (8,726, 9,018)	1,058 (866, 1,158)	6.4 (5.2, 7.0)	11.9 (9.7, 14.1)
Male	11,334	9,520 (9,314, 9,627)	1,814 (1,608, 1,921)	11.1 (9.9, 11.8)	19.1 (16.9, 21.3)
Race/ethnicity^e^					
NHW	15,851	14,027 (13,795, 14,148)	1824 (1,591, 1944)	9.3 (8.1, 9.9)	13.0 (11.3, 14.8)
NHB	2,216	1,806 (1,722, 1,850)	410 (327, 455)	10.2 (8.1, 11.3)	22.7 (17.6, 27.9)
Hispanic	1,676	1,495 (1,419, 1,535)	181 (106, 222)	3.0 (1.7, 3.7)	12.1 (6.8, 17.5)
Wave^f^					
Wave I	2,059	1,967 (1,875, 2,016)	92 (0, 141)	0.3 (0.0, 0.4)	4.7 (0.2, 9.2)
Wave II	2,478	2,075 (1,981, 2,126)	403 (308, 453)	1.2 (0.9, 1.4)	19.4 (14.8, 24.2)
Wave III	6,201	5,471 (5,317, 5,551)	730 (576, 810)	2.2 (1.7, 2.4)	13.3 (10.5, 16.2)
Wave IV	3,559	2,874 (2,763, 2,933)	685 (574, 744)	2.1 (1.7, 2.2)	23.8 (19.8, 27.9)
Wave V	4,481	3,941 (3,810, 4,009)	540 (410, 609)	1.6 (1.2, 1.8)	13.7 (10.4, 17.1)
Wave VI	2,532	2,185 (2,088, 2,236)	347 (250, 399)	1.0 (0.8, 1.2)	15.9 (11.4, 20.4)
*C. difficile* colitis					
Age					
20–64 years	1,250	808 (741, 844)	442 (375, 478)	2.3 (1.9, 2.5)	54.7 (46.2, 63.4)
65–84 years	11,177	8,004 (7,799, 8,111)	3,173 (2,968, 3,279)	68.1 (63.7, 70.4)	39.6 (37.1, 42.2)
Sex					
Female	11,111	7,998 (7,820, 8,091)	3,113 (2,934, 3,206)	18.7 (17.6, 19.3)	38.9 (36.4, 41.5)
Male	9,422	7,096 (6,931, 7,182)	2,326 (2,161, 2,412)	14.3 (13.2, 14.8)	32.8 (30.1, 35.5)
Race/ethnicity^e^					
NHW	15,999	11,658 (11,430, 11,776)	4,341 (4,113, 4,459)	22.1 (21.0, 22.7)	37.2 (35.1, 39.4)
NHB	2,081	1,395 (1,321, 1,434)	686 (613, 726)	17.1 (15.3, 18.1)	49.2 (42.8, 55.7)
Hispanics	1,283	1,285 (1,215, 1,323)	−2 (−72, 36)	0.0 (−1.2, 0.6)	−0.2 (−5.5, 5.4)
Wave^f^					
Wave I	2,105	1,991 (1,902, 2,038)	114 (26, 161)	0.3 (0.1, 0.5)	5.7 (1.3, 10.3)
Wave II	2,349	2,006 (1,918, 2,054)	343 (254, 390)	1.0 (0.8, 1.2)	17.1 (12.4, 21.9)
Wave III	5,856	4,650 (4,515, 4,721)	1,206 (1,071, 1,277)	3.6 (3.2, 3.8)	25.9 (22.7, 29.2)
Wave IV	3,436	2,195 (2,102, 2,244)	1,241 (1,148, 1,290)	3.7 (3.5, 3.9)	56.5 (51.3, 61.8)
Wave V	4,277	2,798 (2,693, 2,853)	1,479 (1,374, 1,535)	4.5 (4.1, 4.6)	52.9 (48.3, 57.5)
Wave VI	2,510	1,468 (1,392, 1,509)	1,042 (966, 1,083)	3.1 (2.9, 3.3)	71.0 (64.4, 77.7)
Colorectal cancer					
Age					
20–64 years	47,489	46,504 (46,008, 46,759)	985 (489, 1,240)	5.1 (2.5, 6.4)	2.1 (1.2, 3.0)
65–84 years	80,438	74,462 (73,882, 74,760)	5,976 (5,396, 6,274)	128.3 (115.8, 134.7)	8.0 (7.3, 8.8)
Sex					
Female	75,149	72,649 (72,029, 72,967)	2,500 (1,880, 2,819)	15.0 (11.3, 16.9)	3.4 (2.7, 4.2)
Male	87,824	84,278 (83,703, 84,573)	3,546 (2,971, 3,841)	21.7 (18.2, 23.5)	4.2 (3.5, 4.9)
Race/ethnicity^e^					
NHW	119,910	115,342 (114,579, 115,733)	4,568 (3,806, 4,959)	23.3 (19.4, 25.3)	4.0 (3.4, 4.5)
NHB	21,136	20,057 (19,779, 20,200)	1,079 (802, 1,223)	26.8 (20.0, 30.4)	5.4 (4.0, 6.8)
Hispanics	13,527	12,776 (12,530, 12,904)	751 (505, 878)	12.4 (8.3, 14.4)	5.9 (4.1, 7.7)
Wave^f^					
Wave I	16,586	16,243 (15,946, 16,396)	343 (47, 497)	1.0 (0.1, 1.5)	2.1 (0.6, 3.7)
Wave II	20,033	19,699 (19,372, 19,868)	334 (7, 502)	1.0 (0.0, 1.5)	1.7 (0.3, 3.1)
Wave III	46,842	45,030 (44,536, 45,284)	1812 (1,318, 2066)	5.5 (4.0, 6.2)	4.0 (3.1, 5.0)
Wave IV	27,015	25,955 (25,580, 26,149)	1,060 (684, 1,253)	3.2 (2.1, 3.8)	4.1 (2.8, 5.3)
Wave V	32,039	30,877 (30,467, 31,087)	1,162 (753, 1,373)	3.5 (2.3, 4.1)	3.8 (2.6, 4.9)
Wave VI	20,458	19,772 (19,444, 19,941)	686 (359, 855)	2.1 (1.1, 2.6)	3.5 (2.1, 4.9)

Abbreviations: CI, confidence interval; GI, gastrointestinal; NOS, not otherwise specified; *C. difficile*, *Clostridium difficile*; NHW, non-Hispanic Whites; NHB, non-Hispanic Blacks.

^a^ The contributing cause of death was adopted.

^b^ Excess death number estimated by subtract the expected number from the observed number of death.

^c^ Excess mortality per 1,000,000 persons was estimated via the excess death number divided by population size.

^d^ Excess risk was calculated as the ratio of the excess-to-expected number of death.

^e^ Non-Hispanic unknown and non-Hispanic AIAN was excluded when stratified by race/ethnicity.

^f^ Waves were identified according to the weekly surveillance of COVID-19 deaths in the US. Wave I was from March 2020 to June 2020, Wave II was from June 2020 to October 2020, Wave III was from October 2020 to June 2021, Wave IV was from June 2021 to November 2021, Wave V was from November 2021 to May 2022 and Wave VI was from May 2022 to September 2022.

Higher ERs of liver disease were more likely occurred in young adults, including ALD (23.0% in adults aged 20–64 years vs. 15.9% in adults aged 65–84 years), fibrosis/cirrhosis (16.0% vs. 6.8%), and hepatic failure (13.0% vs. 4.6%), but not for AP (19.7% vs. 44.4%) (Table 3 and Supplementary Tables S9–S11). There were higher excess death counts of liver diseases among the non-Hispanic Whites, while higher ERs were observed among the non-Hispanic Blacks or Hispanics. The ERs of ALD in non-Hispanic Blacks, Hispanics, and non-Hispanic Whites were 27.4%, 23.6%, and 16.8%, respectively. The ERs in Hispanics and non-Hispanic Whites were 27.1% vs. 14.0% for chronic hepatitis C, 23.6% vs. 7.2% for fibrosis/cirrhosis, and 13.4% vs. 6.7% for hepatic failure, respectively. The excess estimates for AP were more pronounced in non-Hispanic Whites and non-Hispanic Blacks than that in Hispanics. The sex disparity was observed for chronic hepatitis C (ER 23.6% in females vs. 12.4% in males).

**TABLE 3:** Excess mortality associated with selected liver and pancreatic diseases by demographic factor and pandemic wave (United States, March 2020 to September 2022).

Cause of death^a^	Observed deaths, No.	Expected deaths No. (95% CI)	Excess deaths No. (95% CI)^b^	Excess mortality (95% CI)^c^	Excess risk % (95% CI)^d^
Alcoholic liver disease					
Age					
20–64 years	76,511	62,213 (61,701, 62,476)	14,298 (13,787, 14,561)	73.8 (71.1, 75.1)	23.0 (22.1, 23.9)
65–84 years	27,348	23,599 (23,269, 23,769)	3,749 (3,419, 3,919)	80.5 (73.4, 84.1)	15.9 (14.5, 17.3)
Sex					
Female	32,163	26,324 (26,006, 26,488)	5,839 (5,521, 6,003)	35.1 (33.2, 36.0)	22.2 (20.8, 23.5)
Male	73,449	61,495 (61,009, 61,745)	11,954 (11,468, 12,204)	73.2 (70.3, 74.8)	19.4 (18.6, 20.3)
Race/ethnicity^e^					
NHW	72,923	62,452 (61,962, 62,704)	10,471 (9,981, 10,723)	53.4 (50.9, 54.7)	16.8 (15.9, 17.6)
NHB	8,472	6,652 (6,487, 6,738)	1,820 (1,655, 1,906)	45.3 (41.2, 47.4)	27.4 (24.7, 30.1)
Hispanics	16,669	13,483 (13,242, 13,609)	3,186 (2,944, 3,311)	52.4 (48.4, 54.5)	23.6 (21.8, 25.5)
Wave^f^					
Wave I	9,457	8,639 (8,452, 8,736)	818 (632, 916)	2.5 (1.9, 2.8)	9.5 (7.3, 11.7)
Wave II	13,166	10,571 (10,365, 10,678)	2,595 (2,388, 2,702)	7.8 (7.2, 8.1)	24.5 (22.4, 26.7)
Wave III	31,286	24,947 (24,630, 25,111)	6,339 (6,022, 6,502)	19.1 (18.1, 19.6)	25.4 (24.0, 26.8)
Wave IV	18,434	14,566 (14,324, 14,691)	3,868 (3,626, 3,994)	11.7 (10.9, 12.0)	26.6 (24.7, 28.4)
Wave V	20,945	17,864 (17,595, 18,002)	3,081 (2,813, 3,220)	9.3 (8.5, 9.7)	17.2 (15.7, 18.8)
Wave VI	12,324	11,494 (11,279, 11,606)	830 (615, 942)	2.5 (1.9, 2.8)	7.2 (5.3, 9.1)
Chronic hepatitis C					
Age					
20–64 years	16,749	14,763 (14,508, 14,895)	1,986 (1,731, 2,118)	10.2 (8.9, 10.9)	13.5 (11.7, 15.2)
65–84 years	14,793	12,573 (12,304, 12,713)	2,220 (1,950, 2,359)	47.7 (41.9, 50.6)	17.7 (15.8, 19.6)
Sex					
Female	10,436	8,446 (8,266, 8,540)	1,990 (1,810, 2,084)	12.0 (10.9, 12.5)	23.6 (21.2, 25.9)
Male	25,104	22,344 (22,051, 22,495)	2,760 (2,467, 2,912)	16.9 (15.1, 17.8)	12.4 (11.0, 13.7)
Race/ethnicity^e^					
NHW	22,453	19,688 (19,401, 19,836)	2,765 (2,478, 2,914)	14.1 (12.6, 14.9)	14.0 (12.6, 15.5)
NHB	6,393	5,398 (5,254, 5,473)	995 (851, 1,070)	24.8 (21.2, 26.6)	18.4 (15.5, 21.4)
Hispanic	4,488	3,530 (3,405, 3,596)	958 (832, 1,024)	15.8 (13.7, 16.8)	27.1 (23.4, 30.9)
Wave^f^					
Wave I	4,065	3,598 (3,475, 3,662)	467 (345, 532)	1.4 (1.0, 1.6)	13.0 (9.5, 16.5)
Wave II	4,731	4,032 (3,902, 4,100)	699 (569, 767)	2.1 (1.7, 2.3)	17.3 (14.0, 20.7)
Wave III	10,560	9,206 (9,010, 9,308)	1,354 (1,157, 1,456)	4.1 (3.5, 4.4)	14.7 (12.5, 16.9)
Wave IV	5,715	4,835 (4,692, 4,909)	880 (738, 955)	2.7 (2.2, 2.9)	18.2 (15.2, 21.3)
Wave V	6,708	5,804 (5,648, 5,885)	904 (748, 986)	2.7 (2.3, 3.0)	15.6 (12.8, 18.4)
Wave VI	3,761	3,394 (3,275, 3,457)	367 (247, 429)	1.1 (0.7, 1.3)	10.8 (7.3, 14.4)
Fibrosis/cirrhosis					
Age					
20–64 years	55,791	48,102 (47,546, 48,387)	7,689 (7,133, 7,975)	39.7 (36.8, 41.1)	16.0 (15.0, 16.9)
65–84 years	70,205	65,708 (65,183, 65,977)	4,497 (3,972, 4,767)	96.5 (85.3, 102.3)	6.8 (6.1, 7.6)
Sex					
Female	58,190	52,913 (52,446, 53,153)	5,277 (4,810, 5,518)	31.7 (28.9, 33.1)	10.0 (9.1, 10.9)
Male	78,807	72,864 (72,321, 73,142)	5,943 (5,401, 6,222)	36.4 (33.1, 38.1)	8.2 (7.4, 8.9)
Race/ethnicity^e^					
NHW	97,028	90,493 (89,850, 90,823)	6,535 (5,892, 6,865)	33.3 (30.1, 35.0)	7.2 (6.5, 7.9)
NHB	11,965	11,197 (10,969, 11,315)	768 (540, 886)	19.1 (13.4, 22.0)	6.9 (5.0, 8.8)
Hispanics	20,576	16,647 (16,381, 16,785)	3,929 (3,663, 4,067)	64.6 (60.2, 66.9)	23.6 (21.9, 25.3)
Wave^f^					
Wave I	12,871	12,564 (12,308, 12,696)	307 (51, 440)	0.9 (0.2, 1.3)	2.4 (0.7, 4.2)
Wave II	16,352	14,736 (14,459, 14,879)	1,616 (1,339, 1,759)	4.9 (4.0, 5.3)	11.0 (9.3, 12.7)
Wave III	39,900	36,079 (35,646, 36,303)	3,821 (3,387, 4,044)	11.5 (10.2, 12.2)	10.6 (9.5, 11.7)
Wave IV	23,043	20,475 (20,148, 20,643)	2,568 (2,242, 2,737)	7.7 (6.8, 8.2)	12.5 (11.1, 14.0)
Wave V	28,129	26,213 (25,844, 26,404)	1,916 (1,546, 2,106)	5.8 (4.7, 6.3)	7.3 (6.1, 8.6)
Wave VI	16,702	16,189 (15,899, 16,339)	513 (223, 663)	1.5 (0.7, 2.0)	3.2 (1.6, 4.7)
Hepatic failure					
Age					
20–64 years	39,474	34,923 (34,507, 35,137)	4,551 (4,135, 4,765)	23.5 (21.3, 24.6)	13.0 (11.9, 14.1)
65–84 years	33,526	32,057 (31,673, 32,254)	1,469 (1,086, 1,667)	31.5 (23.3, 35.8)	4.6 (3.5, 5.7)
Sex					
Female	34,853	32,871 (32,516, 33,055)	1,982 (1,626, 2,165)	11.9 (9.8, 13.0)	6.0 (4.9, 7.1)
Male	44,412	40,844 (40,442, 41,051)	3,568 (3,166, 3,775)	21.9 (19.4, 23.1)	8.7 (7.7, 9.7)
Race/ethnicity^e^					
NHW	54,979	51,539 (51,086, 51,772)	3,440 (2,987, 3,673)	17.6 (15.2, 18.7)	6.7 (5.8, 7.6)
NHB	8,881	8,152 (7,975, 8,244)	729 (552, 821)	18.1 (13.7, 20.4)	8.9 (6.7, 11.2)
Hispanics	10,343	9,120 (8,933, 9,218)	1,223 (1,035, 1,320)	20.1 (17.0, 21.7)	13.4 (11.2, 15.6)
Wave^f^					
Wave I	7,433	7,625 (7,438, 7,722)	−192 (−379, −95)	−0.6 (−1.1, −0.3)	−2.5 (−4.7, −0.3)
Wave II	9,599	8,879 (8,677, 8,983)	720 (519, 825)	2.2 (1.6, 2.5)	8.1 (6.0, 10.3)
Wave III	23,338	21,275 (20,963, 21,436)	2,063 (1,751, 2,224)	6.2 (5.3, 6.7)	9.7 (8.3, 11.1)
Wave IV	13,496	11,985 (11,750, 12,106)	1,511 (1,277, 1,633)	4.6 (3.8, 4.9)	12.6 (10.7, 14.5)
Wave V	15,832	15,006 (14,743, 15,141)	826 (564, 962)	2.5 (1.7, 2.9)	5.5 (3.9, 7.2)
Wave VI	9,567	9,222 (9,016, 9,329)	345 (140, 452)	1.0 (0.4, 1.4)	3.7 (1.7, 5.8)
Acute pancreatitis					
Age					
20–64 years	5,174	4,323 (4,086, 4,446)	851 (614, 974)	4.4 (3.2, 5.0)	19.7 (16.4, 23.0)
65–84 years	6,078	4,209 (3,934, 4,352)	1,869 (1,593, 2,011)	40.1 (34.2, 43.2)	44.4 (40.8, 48.1)
Sex					
Female	7,323	5,945 (5,783, 6,029)	1,378 (1,217, 1,463)	8.3 (7.3, 8.8)	23.2 (20.4, 26.0)
Male	10,974	9,178 (8,967, 9,288)	1,796 (1,584, 1,906)	11.0 (9.7, 11.7)	19.6 (17.3, 21.8)
Race/ethnicity^e^					
NHW	13,166	10,793 (10,576, 10,906)	2,373 (2,155, 2,485)	12.1 (11.0, 12.7)	22.0 (19.9, 24.1)
NHB	2,382	1,971 (1,884, 2,017)	411 (324, 457)	10.2 (8.1, 11.4)	20.9 (16.0, 25.8)
Hispanics	1,582	1,665 (1,585, 1,708)	−83 (−163, −41)	−1.4 (−2.7, −0.7)	−5.0 (−9.6, −0.2)
Wave^f^					
Wave I	1,806	1,553 (1,471, 1,596)	253 (172, 297)	0.8 (0.5, 0.9)	16.3 (11.0, 21.7)
Wave II	2,264	1,819 (1,731, 1,866)	445 (357, 492)	1.3 (1.1, 1.5)	24.5 (19.4, 29.6)
Wave III	5,365	4,357 (4,221, 4,429)	1,008 (871, 1,079)	3.0 (2.6, 3.3)	23.1 (19.9, 26.5)
Wave IV	2,994	2,444 (2,342, 2,498)	550 (448, 604)	1.7 (1.3, 1.8)	22.5 (18.2, 26.9)
Wave V	3,647	3,100 (2,985, 3,160)	547 (433, 608)	1.6 (1.3, 1.8)	17.6 (13.9, 21.5)
Wave VI	2,221	1,898 (1,808, 1,946)	323 (233, 371)	1.0 (0.7, 1.1)	17.0 (12.2, 21.9)

Abbreviations: CI, confidence interval; NOS, not otherwise specified; NHW, non-Hispanic Whites; NHB, non-Hispanic Blacks.

^a^ The contributing cause of death was adopted.

^b^ Excess death number estimated by subtract the expected number from the observed number of death.

^c^ Excess mortality per 1,000,000 persons was estimated via the excess death number divided by population size.

^d^ Excess risk was calculated as the ratio of the excess-to-expected number of death.

^e^ Non-Hispanic unknown and non-Hispanic AIAN, was excluded when stratified by race/ethnicity.

^f^ Waves were identified according to the weekly surveillance of COVID-19 deaths in the US. Wave I was from March 2020 to June 2020, Wave II was from June 2020 to October 2020, Wave III was from October 2020 to June 2021, Wave IV was from June 2021 to November 2021, Wave V was from November 2021 to May 2022 and Wave VI was from May 2022 to September 2022.

The temporal patterns of excess digestive-related death risks were different by subtype disease. For most selected GI, liver and pancreatic diseases, the ERs were more pronounced since Wave II and highest in Wave IV (Tables 2, 3). For GI hemorrhage and fibrosis/cirrhosis, we observed fluctuating temporal trends with three clear peaks in Wave III, Wave IV, and Wave V (Figure 1 and Supplementary Figures S1, S2). For *C. difficile* colitis, there showed a persistently increasing trend during the pandemic. Although for ulcers, ALD, CRC, chronic hepatitis C, AP, etc., there were less varied temporal trends, excess deaths of these subtype diseases were consistently observed across different waves, even in the mild Omicron BA.2 wave (Wave VI).

**FIGURE 2:**
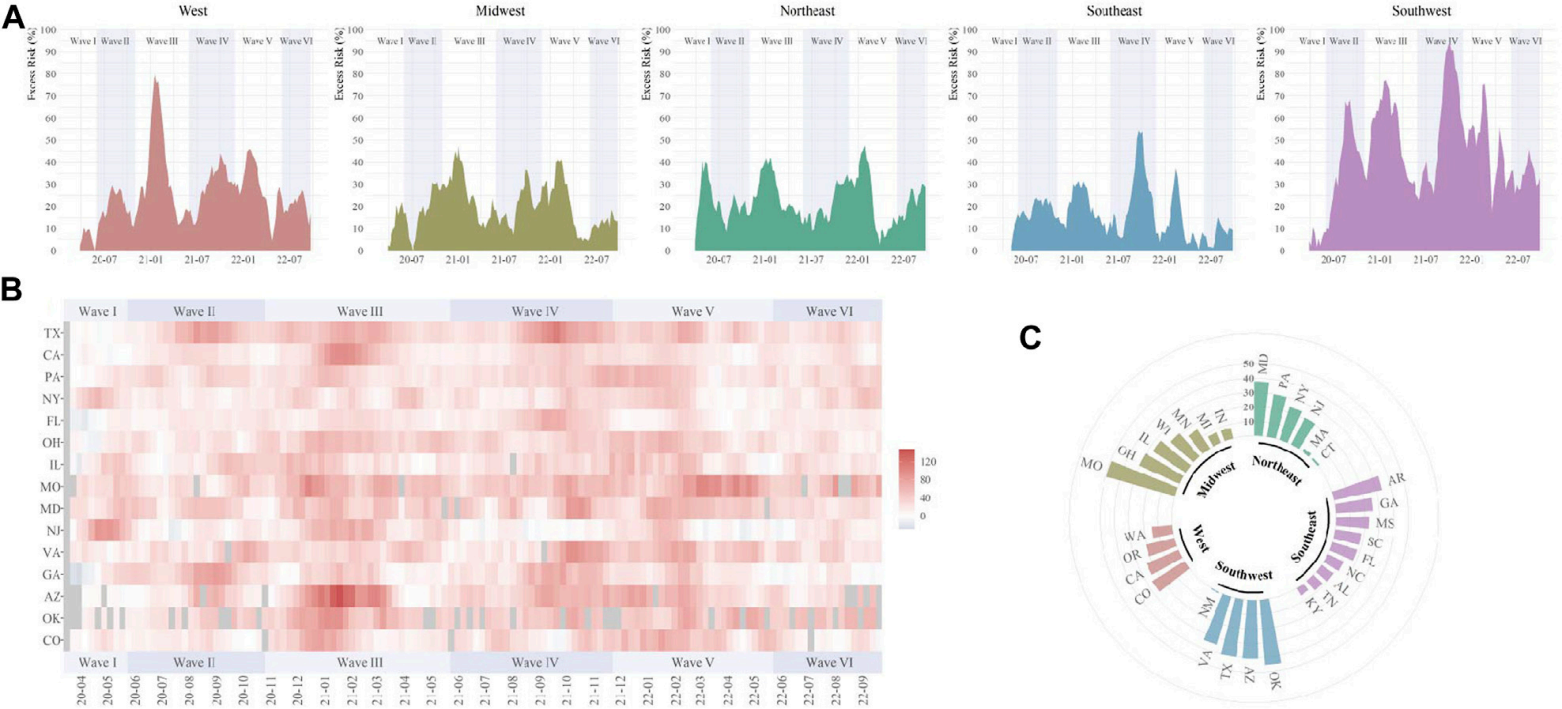
Temporal and spatial excess risks (%) associated with gastrointestinal hemorrhage (United States, March 2020 to September 2022). **(A)** temporal variated excess risks stratified by region; **(B)** temporal variated excess risks stratified by state, where the 4-week moving average was operated for each time series; **(C)** overall estimates by states (states with negative estimated values were not shown). The contributing cause of death was adopted. The six COVID-19 death waves were defined as Wave I (March 2020 to June 2020), Wave II (June 2020 to October 2020), Wave III (October 2020 to June 2021), Wave IV (June 2021 to November 2021), Wave V (November 2021 to May 2022), and Wave VI (May 2022 to September 2022).

**FIGURE 3:**
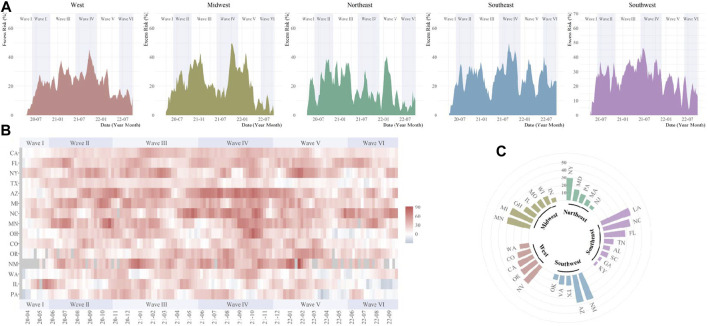
Temporal and spatial excess risks (%) associated with alcoholic liver disease (United States, March 2020 to September 2022). **(A)** temporal variated excess risks stratified by region; **(B)** temporal variated excess risks stratified by state, where the 4-week moving average was operated for each time series; **(C)** overall estimates by states (states with negative estimated values were not shown). The contributing cause of death was adopted. The six COVID-19 death waves were defined as Wave I (March 2020 to June 2020), Wave II (June 2020 to October 2020), Wave III (October 2020 to June 2021), Wave IV (June 2021 to November 2021), Wave V (November 2021 to May 2022), and Wave VI (May 2022 to September 2022).

The temporal variations of excess digestive-related death risks were different across regions. For GI hemorrhage, the substantial ER increase was firstly observed in the Northeast and Midwest in Wave I, then, expanded to all five regions (Figure 2). Higher ER waves were observed in the West in Wave III and Southwest in Wave II to V. During the pandemic, Missouri, Oklahoma, Texas, and Arizona were affected most for GI hemorrhage (ER range: 40.4%–49.3%) (Supplementary Tables S12–S16). For ALD, temporal fluctuations were variated across regions (Figure 3). Temporal and spatial excess risks associated with colorectal cancer, hepatic fibrosis/cirrhosis, and hepatic failure were shown in Supplementary Figures S3–S5. The major proportion of ALD excess risks occurred in Wave II to IV. Louisiana, North Carolina, Arizona, and New Mexico showed the highest increases in ERs of ALD. The stratification estimates by digestive system diseases, digestive organ malignancies, and gastrointestinal hemorrhage were shown in Supplementary Tables S17–S19. Results from sensitivity analyses showed the robustness of the estimates (Supplementary Tables S20–S25).

## Discussion

In this study, we provided updated evidence on excess mortality associated with GI, liver, and pancreatic diseases in the US during the nearly 3-year pandemic. We found prominent increased death risks among persons with GI hemorrhage, *C. difficile* colitis, ulcer, ALD, AP, chronic hepatitis C, and fibrosis/cirrhosis. Those excess deaths showed age and racial/ethnic disparities. Higher ERs were generally shown among non-Hispanic Blacks and Hispanics. We observed varied temporal patterns of excess deaths for different causes of death. The trends for GI hemorrhage and fibrosis/cirrhosis fluctuated across multiple pandemic waves, the trends for ulcers, ALD, chronic hepatitis C, AP, etc., have been persistently observed throughout the pandemic, whereas that for *C. difficile* colitis has continuously increased.

The increased deaths in our results included those with co-occurrence of SARS-CoV-2 infection and digestive diseases, those with impairment of digestive systems induced by SARS-CoV-2 infection, and those with underlying digestive conditions indirectly affected by the pandemic. Our study identified deaths according to the contributing causes of death listed on the death certificate (could contain a maximum of 20 causes). Since many digestive diseases are more likely to be considered as contributing factors than the underlying factors of death, our primary estimations based on the contributing causes of death could avoid underestimation and reflect the overall mortality burden among individuals with GI, liver, and pancreatic abnormalities during the COVID-19 pandemic.

Patients with underlying GI conditions are vulnerable to adverse COVID-19 outcomes [12, 13]. GI involvement has been reported among patients with COVID-19 infections with an incidence ranging from 11% to 39% [14–17]. The common GI manifestation included anorexia, nausea, vomiting, dysgeusia, and diarrhea, which could be explained by the high expression of angiotensin-converting enzyme 2 (ACE2) receptors in the gut [18]. In addition, critically ill patients with COVID-19 were more likely to develop GI complications and were associated with adverse outcomes [19, 20]. Our results showed higher ERs in GI hemorrhage and ulcer-related mortality during the pandemic. Although GI bleeding was uncommon among patients with COVID-19 [21, 22], it was one of the most prevalent GI diagnoses for emergency department visits or hospitalization before the pandemic, requiring timely endoscopic evaluation and treatments [10]. However, GI endoscopy is an aerosol-generating procedure which may facilitate virus transmission. In the early pandemic, more than 60% of endoscopy centers in the US reported an over 60% decrease in volume [23]. Such delay could lead to difficulty in localizing GI hemorrhage, which was further associated with prolonged hospitalization, increased deaths, or re-admission owing to rebleeding in patients even without SARS-CoV-2 infection [24]. In addition, GI mucosal damage, including acute musical injury and ischemic colitis, was found in COVID patients, which may aggravate ulcers and hemorrhage [25]. Also, anticoagulant use during COVID treatments may pose additional risks of GI bleeding for some patients [26].

We found the temporal fluctuation of excess deaths of GI hemorrhage. It might reflect the magnitude of overburdened healthcare services for GI patients across multiple pandemic waves. In contrast, there was a persistently increasing trend for excess deaths related to *C. difficile* colitis. During the SARS-CoV-2 infection, part of patients may experience changed gut microbiota composition. Frequent prescription of antibiotics to treat or prevent infections complicated with COVID also allowed opportunistic pathogens colonization, which could result in antibiotic-associated diarrhea, pseudomembranous colitis, and even systemic infections, and may further increase the risk of death [27, 28]. The findings suggest the management of GI bleeding and gut microbiota needs to adapt to the COVID-19 era.

Pre-existing chronic liver disease is more likely to experience poor outcomes of COVID due to immune dysregulation and coagulopathy. SARS-CoV-2 can provoke liver injury through systemic inflammation reaction, cytokine signaling, hypoxia ischemia-reperfusion, drug toxicity, and direct infection of hepatocytes [29], which might contribute to an increased death risk among patients with the chronic liver disease [30, 31]. The excess mortality estimates during the pandemic could be an integrated impact of changes in virus virulence, treatment modality, healthcare capacity, vaccination, infection control strategies, chronic disease management, and personal factors (such as hesitation in seeking medication, changed physical activity behavior, dietary intake, and income, etc.). We found the ER of liver-related diseases was predominant in young adults, which were 1.4 times, 2.4 times and 2.8 times higher for ALD, fibrosis/cirrhosis and hepatic failure in young adults than those in older adults, respectively. The reason could be that the pandemic disproportionately affected the lives of individuals. The higher ER of ALD was associated with increased alcohol overconsumption, which was more likely to occur in young adults [32]. Social isolation and difficulty in accessing regular medical care resources might bring additional challenges to patients with chronic liver diseases accessing affordable healthy food since they were advised to keep low-salt diets or other special dietary modifications [33]. Loss of jobs or insurance among those patients could also disrupt their routine costly medications. Those factors may lead to worsening malnutrition, frailty, and other poor outcomes. Substantial stress from social and economic uncertainty can also lead to psychological decompensation and 10%–19% increased drinking compared with the pre-pandemic period [34].

Limited studies reported the difference in GI, liver, and pancreatic cancer-related mortality before and during the pandemic, although there was an evident interruption in cancer management. A global study reported an overall 44.9% decline in the volume of CRC screening [35]. About 10% of patients reported their postponed curative surgery due to a COVID-related reason [36]. Moreover, GI cancer patients after operation might be at a high risk of death if infected with SARS-CoV-2 [37]. Therefore, it is crucial to comprehensively evaluate both risks and benefits of postponing or canceling elective surgery during the pandemic. Although there could be a growing mortality risk among patients with undetected cancer or postponed surgery, we did not observe such excess deaths for GI, liver, or pancreatic cancer so far. Nevertheless, continually monitoring the mortality is important for healthcare authorities to make a timely intervention in response to changing situations.

With substantial population recovering from COVID-19, the long-term impact of COVID-19 is raising the concern. Although fatal sequelae of the GI system were not reported in COVID-19 survivors, there were increased risks of GI disorders, dysphagia and abdominal pain with hazard ratios varied from 2.8 to 6.9 [38]. In addition, malnutrition remained at 54% and 32% among patients with distinct GI manifestations at 3- and 6-month of follow-up, respectively [39]. The readmission rate of AP after 1 year of recovery from COVID-19 was about 14%, most of whom were due to alcohol-related or gallstone pancreatitis [40]. Our study observed persistent excess deaths related to the GI system and an increasing trend for *C. difficile* colitis during the pandemic. The trends are likely to remain even after WHO announced the end of PHEIC. However, our results cannot distinguish the cause of death from the acute or post-acute stage. Further studies are warranted to understand the long-term impact of COVID-19 on the digestive system. As we will live with SARS-CoV-2, sustainable healthcare policy and resources are needed to meet the “new normal.”

### Limitations

There are several limitations should be noted. First, the provisional weekly mortality data may suffer from potential incompleteness due to reporting delay. We restricted data before September 2022 to minimize the effect of data incompleteness from the analysis. Second, due to weekly data availability, we only performed analysis for 15 selected causes of death, which account for most deaths for all GI, liver, and pancreatic-related deaths. We excluded other causes of death from analysis as the weekly stratification data was quite sparse. Thus, our results might not fully reflect the whole impact on the digestive system burden. In addition, we only had a 2-year reference to fit the regression model. There could be an uncertainty of long-term variation in mortality. Third, our results might be underestimated if some cases were delayed for diagnostic testing of abnormality during the pandemic. It highlights the importance to maintain regular diagnostic volume and treatment capacity for patients with underlying GI, liver, or pancreatic conditions. Fourth, since individual data was not available in our study, we did not investigate the mortality burden directly caused by COVID-19 infection or indirectly caused by the pandemic. Further studies are also warranted to understand changes in digestive system burden in ambulatory visits, emergency department visits, medication, and hospitalization during the pandemic. Last, for demographic stratification and regional comparisons, reporting findings were more likely to choose those with higher excess estimate values. It should be aware that those subgroups may have different age structures, socioeconomic characteristics, and infection control measures. The results should be carefully interpreted when using excess mortality. Nevertheless, estimates by the risk (ER) could limit the bias.

### Conclusion

This study identified the increased mortality associated with GI, liver, and pancreas diseases with demographic disparities and varied temporal and spatial patterns during the pandemic. As SARS-CoV-2 may continually circulate in the community and lead to potential epidemic waves in the future, it is important for pertinent stakeholders to further evaluate the long-term impact of the pandemic on healthcare workers and patients. It is also necessary to develop sustainable strategies to improve the diagnostic, treatment, and disease management capacities of patients with GI, liver, and pancreatic conditions in the era of living with COVID-19.

## Data Availability

All original data used in this work are publicly available, shown at: https://wonder.cdc.gov/controller/datarequest/D176. The data and syntax for analysis have been uploaded at: https://github.com/ran1991/excessDIGEST.

## Abbreviations

ALD, alcohol liver disease; AP, acute pancreatitis; CI, confidence interval; *C. difficile*, *Clostridium difficile*; CRC, colorectal cancer; EC, esophageal cancer; GC, gastric cancer; GI, gastrointestinal; LIHC, liver and intrahepatic cancer; NHB, non-Hispanic Blacks; NHW, non-Hispanic Whites; NOS, not otherwise specified; PC, pancreatic cancer; PHEIC, Public Health Emergency of International Concern; WHO, World Health Organization.
