# Supplementary material for: Excess Deaths of Gastrointestinal, Liver, and Pancreatic Diseases During the COVID-19 Pandemic in the United States
Source: Int J Public Health. 2023 Aug 15;68:1606305. doi: 10.3389/ijph.2023.1606305 (PMC10462779; doi:10.3389/ijph.2023.1606305)
Supplement: Supplementary file 1 [file DataSheet1.docx]

International Journal of Public Health

Excess Deaths of Gastrointestinal, Liver, and Pancreatic Diseases During the COVID-19 Pandemic in the United States

Supplementary Information

Table of Contents

[Table S1. ICD-10 codes of digestive related diseases (United States. 2020-2022) 4](#_Toc141917703)

[Table S2. Information about pandemic years and pandemic waves (United States. 2020-2022) 6](#_Toc141917704)

[Table S3. The dispersion parameter value of regression models for cause-specific diseases (United States. 2020-2022) 7](#_Toc141917705)

[Table S4. qAIC score of digestive related diseases by knots (United States. 2020-2022) 8](#_Toc141917706)

[Table S5. Excess mortality associated with digestive related diseases from March 2020 to September 2022. Digestive related diseases were the underlying causes of death (United States. 2020-2022) 9](#_Toc141917707)

[Table S6. Excess mortality associated with digestive related diseases from March 2020 to February 2021 (United States. 2020-2022) 10](#_Toc141917708)

[Table S7. Excess mortality associated with digestive related diseases from March 2021 to February 2022 (United States. 2020-2022) 11](#_Toc141917709)

[Table S8. Excess mortality associated with digestive related diseases from March 2022 to September 2022 (United States. 2020-2022) 12](#_Toc141917710)

[Table S9. Excess mortality associated with selected digestive related diseases stratified by demographic factor from March 2020 to February 2021 (United States. 2020-2022) 13](#_Toc141917711)

[Table S10. Excess mortality associated with selected digestive related diseases stratified by demographic factor from March 2021 to February 2022 (United States. 2020-2022) 17](#_Toc141917712)

[Table S11. Excess mortality associated with selected digestive related diseases stratified by demographic from March 2022 to September 2022 (United States. 2020-2022) 21](#_Toc141917713)

[Table S12. The estimated excess mortality of gastrointestinal hemorrhage by state from March 2020 to September 2022 (United States. 2020-2022) 25](#_Toc141917714)

[Table S13. The estimated excess mortality of colorectal cancer by state from March 2020 to September 2022 (United States. 2020-2022) 27](#_Toc141917715)

[Table S14. The estimated excess mortality of alcoholic liver disease by state from March 2020 to September 2022 (United States. 2020-2022) 29](#_Toc141917716)

[Table S15. The estimated excess mortality of hepatic fibrosis/cirrhosis by state from March 2020 to September 2022 (United States. 2020-2022) 31](#_Toc141917717)

[Table S16. The estimated excess mortality of hepatic failure by state from March 2020 to September 2022 (United States. 2020-2022) 33](#_Toc141917718)

[Table S17. Excess mortality associated with digestive system diseases stratified by demographic factors and wave (United States. 2020-2022) 35](#_Toc141917719)

[Table S18. Excess mortality associated with digestive organ malignancies stratified by demographic factors and wave (United States. 2020-2022) 36](#_Toc141917720)

[Table S19. Excess mortality associated with gastrointestinal hemorrhage stratified by demographic factors and wave (United States. 2020-2022) 37](#_Toc141917721)

[Table S20. Sensitivity analysis of excess mortality associated with digestive related diseases from March 2020 to September 2022.^a^ (United States. 2020-2022) 38](#_Toc141917722)

[Table S21. Sensitivity analysis of excess mortality associated with digestive related diseases from March 2020 to September 2022.^a^ (United States. 2020-2022) 39](#_Toc141917723)

[Table S22. Sensitivity analysis of excess mortality associated with selected gastrointestinal diseases stratified by demographic factor and pandemic wave. ^a^ (United States. 2020-2022) 40](#_Toc141917724)

[Table S23. Sensitivity analysis of excess mortality associated with selected liver and pancreatic diseases stratified by demographic factor and pandemic wave. ^a^ (United States. 2020-2022) 43](#_Toc141917725)

[Table S24. Sensitivity analysis of excess mortality associated with selected gastrointestinal diseases stratified by demographic factor and pandemic wave. ^a^ (United States. 2020-2022) 46](#_Toc141917726)

[Table S25. Sensitivity analysis of excess mortality associated with selected liver and pancreatic diseases stratified by demographic factor and pandemic wave. ^a^ (United States. 2020-2022) 49](#_Toc141917727)

[Fig. S1 Weekly estimates of excess risks (%) associated with selected gastrointestinal, liver, and pancreatic diseases (United States. 2020-2022) 52](#_Toc141917728)

[Fig. S2 Weekly estimates of excess deaths for A) GI hemorrhage, B) Alcoholic liver disease and C) Hepatic fibrosis/cirrhosis by demographic characteristic (United States. 2020-2022). 53](#_Toc141917729)

[Fig. S3 Temporal and spatial excess risks (%) associated with colorectal cancer in the United States (United States. 2020-2022). 54](#_Toc141917730)

[Fig. S4 Temporal and spatial excess risk (%) associated with hepatic fibrosis/cirrhosis in the United States (United States. 2020-2022) 55](#_Toc141917731)

[Fig. S5 Temporal and spatial excess risks (%) associated with hepatic failure (United States. 2020-2022) 56](#_Toc141917732)

# Table S1. ICD-10 codes of digestive related diseases (United States. 2020-2022)

| **Diseases** | **Abbreviations** | **ICD-10 codes** | **Description** |
| --- | --- | --- | --- |
| **Gastrointestinal diseases** | | | |
| Gastrointestinal hemorrhage, not otherwise specified | GI hemorrhage, NOS | K92.2 | Hemorrhage of the gastrointestinal tract with not otherwise specified, including gastric, NOS and intestinal, NOS. Did not include hemorrhage with peptic ulcer. |
| Ulcers (gastric/duodenal/peptic) | Ulcers | K25, K26, K27, K28 | Included erosion (acute) of stomach and ulcers of the stomach or pylorus (K25); erosion (acute) of and duodenal or postpyloric ulcers (K26); gastroduodenal ulcers, NOS, and peptic ulcers, NOS (K27); gastrojejunal ulcer or erosion (K28), with or without acute/chronic/unspecified hemorrhage and/or perforation.   - K25 did not include acute hemorrhagic erosive gastritis and gastric ulcers, NOS. - K26 did not include gastric ulcers, NOS. - K28 did not include primary ulcer of the small intestine (K63.3). - Did not include anus and rectum ulcer (K62.6), ulcer of the small intestine (K63.3) and ulcerate colitis (K51). |
| *Clostridium difficile* colitis | *C. difficile* colitis | A04.7 | Included food poisoning caused by *C. difficile* and pseudomembranous colitis. |
| Vascular disorders of the Intestine | - | K55.0, K55.1, K55.2, K55.8, K55.9 | Included acute vascular disorder of intestine (K55.0, fulminant ischemic colitis; intestinal infarction; small intestine ischemia; embolism, thrombosis or infarction of mesenteric artery or vein; and subacute ischemic colitis), chronic vascular disorder of intestine (K55.1, chronic ischemic colitis, enteritis or enterocolitis; ischemic stricture of intestine mesenteric atherosclerosis or vascular insufficiency), angiodysplasia of colon (K55.2), angiodysplasia of small intestine (K55.3), other intestinal vascular disorder of intestine (K55.8), and intestine vascular disorder, NOS (K55.9, ischemic colitis, enteritis or enterocolitis, NOS). |
| Paralytic ileus and intestinal obstruction | - | K56.0, K56.1, K56.2, K56.3, K56.4, K56.5, K56.6, K56.7 | Included paralytic ileus (K56.0), intussusception (K56.1), volvulus (K56.2), gallstone ileus (K56.3), other impaction of intestine (K56.4), intestinal adhesions with obstruction (K56.5), other and unspecified intestinal obstruction (K56.6), and ileus, NOS (K56.7) |
| Esophageal cancer | EC | C15.3, C15.4, C15.5, C15.8, C15.9 | Included the malignancies of the upper (C15.3), middle (C15.4) and lower (C15.5) third of the esophagus and overlapping lesion of esophageal (C15.8) and esophagus, NOS (C15.9). |
| Gastric cancer | GC | C16.0, C16.1, C16.2, C16.3, C16.4, C16.5, C16.6, C16.8, C16.9 | Malignancies of the stomach. Included malignancies of the cardia (C16.0), fundus (C16.1), body (C16.2), pyloric antrum (C16.3), pylorus (C16.4) of stomach, lesser curvature of stomach, unspecified (C16.5), greater curvature of stomach, unspecified (C16.6), overlapping lesion of stomach (C16.8) and malignancies of stomach, NOS (C16.9). |
| Colorectal cancer | CRC | C18, C19, C20, C21 | Malignancies of the colon (C18), rectosigmoid junction (C19), rectum (C20), and anus and anal canal (C21). |
| **Liver and pancreatic diseases** | | | |
| Alcoholic liver disease | ALD | K70.0, K70.1, K70.2, K70.3, K70.4, K70.9 | Included alcoholic fatty liver (K70.0), alcoholic hepatitis (K70.1), alcoholic fibrosis and sclerosis of liver (K70.2), alcoholic cirrhosis of liver (K70.3), alcoholic hepatic failure (K70.4, acute/chronic/subacute, with or without hepatic coma), alcoholic liver disease, NOS (K70.9). |
| Hepatic fibrosis/cirrhosis (all cause) | Fibrosis/cirrhosis | K74.0, K74.1, K74.2, K74.3, K74.5, K74.5, K74.6 | Included hepatic fibrosis (K74.0), hepatic sclerosis (K74.1), hepatic fibrosis with sclerosis (K74.2), primary biliary cirrhosis (K74.3, chronic nonsuppurative destructive cholangitis), secondary biliary cirrhosis (K74.4), biliary cirrhosis, NOS (K74.5), and other and NOS cirrhosis of liver (K74.6). |
| Chronic hepatitis C | - | B18.2 | Chronic viral hepatitis C. |
| Hepatic failure (acute and chronic) | Hepatic failure | K72.0, K72.1, K72.9 | Included acute and subacute hepatic failure (K72.0), chronic hepatic failure (K72.1) and hepatic failure, NOS (K72.9) |
| Liver and intrahepatic bile duct cancer | LIHC | C22.0, C22.1, C22.2, C22.3, C22.4, C22.7, C22.9 | Malignant neoplasm of liver and intrahepatic bile ducts. Included liver cell carcinoma (C22.0, hepatocellular carcinoma and hepatoma), intrahepatic bile duct carcinoma (C22.1), hepatoblastoma (C22.2), angiosarcoma of liver (C22.3), other sarcomas of liver (C22.4), other specified carcinomas of liver (C22.7) and malignancies of liver, NOS (C22.9). |
| Acute pancreatitis | AP | K85.0, K85.1, K85.2, K85.3, K85.5, K85.9 | Included abscess/necrosis of pancreas, acute/hemorrhagic/subacute/suppurative pancreatitis, classified as idiopathic acute pancreatitis (K85.0), biliary acute pancreatitis (K85.1), alcohol-induced acute pancreatitis (K85.2), drug-induced acute pancreatitis (K85.3), other acute pancreatitis (K85.8) and acute pancreatitis, NOS (K85.9). |
| Pancreatic cancer | PC | C25.0, C25.1, C25.2, C25.3, C25.4, C25.7, C25.8, C25.9 | Malignant neoplasm of pancreas. Included malignancies of pancreas head (C25.0), body (C25.1), tail (C25.2), pancreatic duct (C25.3), endocrine pancreas (C25.4), other parts of pancreas (C25.7), overlapping lesion of pancreas (C25.8) and malignancies of pancreas, NOS (C25.9). |

# Table S2. Information about pandemic years and pandemic waves (United States. 2020-2022)

| **Periods** | **Week interval** | **Date interval** | **Length of week** | **Dominate SARS-CoV-2 variants** |
| --- | --- | --- | --- | --- |
| **Pandemic year** |  |  |  |  |
| 1^st^ year | week 10, 2020 – week 8, 2021 | 2020-03-01 to 2021-02-27 | 52 | Original and D614G strain |
| 2^nd^ year | week 9, 2021 – week 8, 2022 | 2021-02-28 to 2022-02-26 | 52 | Alpha, Delta and Omicron BA.1 variant, |
| 3^rd^ year | week 9, 2022– week 38, 2022 | 2022-02-27 to 2022-09-24 | 30 | Omicron BA.2, Omicron BA.5 variant |
| **Pandemic wave** |  |  |  |  |
| Wave I | week 10, 2020 – week 23, 2020 | 2020-03-01 to 2020-06-06 | 14 | Original strain |
| Wave II | week 24, 2020 – week 40, 2020 | 2020-06-07 to 2020-10-03 | 17 | D614G strain |
| Wave III | week 41, 2020 – week 25, 2021 | 2020-10-04 to 2021-06-26 | 38 | D614G strain, Alpha variant |
| Wave IV | week 26, 2021 – week 47, 2021 | 2021-06-27 to 2021-11-27 | 22 | Delta variant |
| Wave V | week 48, 2021 – week 22, 2022 | 2021-11-28 to 2022-05-28 | 26 | Omicron BA.1 variant |
| Wave VI | week 23, 2022 – week 38, 2022 | 2022-05-29 to 2022-09-24 | 17 | Omicron BA.2, Omicron BA.5 variant |

Note: The pandemic waves were defined according to the COVID-19 death waves (shown at <https://covid.cdc.gov/covid-data-tracker/#trends_dailydeaths>). We identified the start and end dates of each wave according to the calendar date of a trough between two consecutive waves.

# Table S3. The dispersion parameter value of regression models for cause-specific diseases (United States. 2020-2022)

| **Cause of disease ^a^** | **Dispersion** | **P value** |
| --- | --- | --- |
| **GI disease** |  |  |
| GI hemorrhage, NOS | 17.42 | ＜0.001 |
| Ulcers | 3.45 | ＜0.001 |
| Paralytic ileus and intestine obstruction | 2.93 | ＜0.001 |
| Vascular disorder of intestine | 2.09 | ＜0.001 |
| *C. difficile* colitis | 3.51 | ＜0.001 |
| EC | 1.18 | 0.055 |
| GC | 1.01 | 0.452 |
| CRC | 2.41 | ＜0.001 |
| **Liver and pancreatic diseases** |  |  |
| ALD | 17.23 | ＜0.001 |
| Fibrosis/cirrhosis | 12.82 | ＜0.001 |
| Chronic hepatitis C | 3.35 | ＜0.001 |
| Hepatic failure | 4.38 | ＜0.001 |
| LIHC | 1.65 | ＜0.001 |
| AP | 3.01 | ＜0.001 |
| PC | 2.20 | ＜0.001 |

Abbreviations: GI, gastrointestinal; NOS, not otherwise specified; *C. difficile*, *Clostridium difficile*; EC, esophageal cancer; GC, gastric cancer; CRC, colorectal cancer; ALD, alcoholic liver disease; LIHC, liver and intrahepatic bile duct cancer; AP, acute pancreatitis; PC, pancreatic cancer.

^a^ The contributing causes of death was adopted.

# Table S4. qAIC score of digestive related diseases by knots (United States. 2020-2022)

| Knot | **GI diseases** | | | | | | | | **Liver and pancreatic diseases** | | | | | | **Sum** |
| --- | --- | --- | --- | --- | --- | --- | --- | --- | --- | --- | --- | --- | --- | --- | --- |
|  | GI hemorrhage, NOS | Ulcer | Paralytic ileus and intestine obstruction | Vascular disorder of intestine | *C. difficile* colitis | EC | GC | CRC | ALD | Fibrosis/  cirrhosis | Chronic hepatitis C | Hepatic failure | LIHC | AP |  |
| 1 | 2297.1 | 1835.1 | 1746.4 | 1676.7 | 2016.8 | 1898.6 | 2054.8 | 1839.7 | 2409.9 | 1940.9 | 2004.0 | 2021.3 | 1874.8 | 1755.9 | 29118.8 |
| 2 | 2069.6 | 1768.8 | 1724.6 | 1657.9 | 1957.7 | 1898.8 | 2062.0 | 1840.4 | 2312.4 | 1848.0 | 1986.3 | 1938.5 | 1875.0 | 1743.4 | 28435.6 |
| 3 | 2019.5 | 1774.8 | 1727.5 | 1664.7 | 1954.7 | 1907.1 | 2070.4 | 1843.9 | 2303.5 | 1852.8 | 1993.4 | 1945.8 | 1882.0 | 1745.3 | 28442.6 |
| 4 | 1948.3 | 1752.4 | 1731.9 | 1664.6 | 1943.4 | 1913.4 | 2078.8 | 1852.8 | 2299.8 | 1850.9 | 1993.8 | 1946.7 | 1880.2 | 1737.5 | 28357.3 |
| 5 | 1912.6 | 1756.5 | 1730.2 | 1669.5 | 1943.3 | 1920.3 | 2087.4 | 1854.6 | 2298.4 | 1849.2 | 1990.6 | 1942.1 | 1884.2 | 1737.1 | 28342.7 |
| 6 | 1853.0 | 1758.1 | 1724.9 | 1668.4 | 1940.2 | 1921.9 | 2092.7 | 1836.6 | 2261.7 | 1833.2 | 1980.7 | 1939.0 | 1880.4 | 1731.5 | 28188.4 |
| 7 | 1849.8 | 1762.5 | 1731.8 | 1677.0 | 1952.1 | 1930.8 | 2092.6 | 1843.5 | 2263.8 | 1842.1 | 1978.3 | 1948.3 | 1887.5 | 1742.8 | 28274.2 |
| 8 | 1849.8 | 1758.1 | 1738.0 | 1680.4 | 1942.5 | 1937.7 | 2100.7 | 1843.8 | 2266.5 | 1839.2 | 1976.1 | 1953.1 | 1892.9 | 1742.5 | 28298.7 |
| 9 | 1846.6 | 1764.0 | 1742.3 | 1684.8 | 1952.8 | 1939.2 | 2103.1 | 1851.6 | 2271.0 | 1836.5 | 1975.4 | 1958.4 | 1897.7 | 1745.7 | 28352.5 |
| 10 | 1853.0 | 1776.0 | 1752.4 | 1692.7 | 1944.9 | 1941.0 | 2108.4 | 1851.2 | 2278.8 | 1835.9 | 1982.7 | 1965.1 | 1903.9 | 1754.7 | 28424.7 |
| 11 | 1852.0 | 1777.1 | 1760.1 | 1700.3 | 1947.3 | 1954.5 | 2115.6 | 1854.5 | 2279.9 | 1841.6 | 1989.1 | 1969.6 | 1911.5 | 1762.2 | 28506.1 |
| 12 | 1854.5 | 1777.9 | 1766.3 | 1705.6 | 1958.2 | 1955.1 | 2117.0 | 1861.4 | 2275.9 | 1847.1 | 1984.5 | 1979.0 | 1918.0 | 1768.8 | 28562.6 |

Abbreviations: qAIC, quasi-akaike information criterion; GI, gastrointestinal; NOS, not otherwise specified; *C. difficile*, *Clostridium difficile*; EC, esophageal cancer; GC, gastric cancer; CRC, colorectal cancer; ALD, Alcoholic liver disease; LIHC, liver and intrahepatic bile duct cancer; AP, acute pancreatitis; PC, pancreatic cancer.

# Table S5. Excess mortality associated with digestive related diseases from March 2020 to September 2022. Digestive related diseases were the underlying causes of death (United States. 2020-2022)

| **Cause of death ^a^** | **Observed deaths, No.** | **Expected deaths**  **No. (95% CI)** | **Excess deaths**  **No. (95% CI) ^b^** | **Excess mortality**  **(95% CI) ^c^** | **Excess risk %**  **(95% CI) ^d^** |  |
| --- | --- | --- | --- | --- | --- | --- |
| **GI diseases** | | | | | |  |
| GI hemorrhage, NOS | 26963 | 23810 (23478, 23982) | 3153 (2820, 3324) | 9.5 (8.5, 10.0) | 13.2 (11.9, 14.6) |  |
| Ulcers | 10160 | 9574 (9382, 9674) | 586 (394, 686) | 1.8 (1.2, 2.1) | 6.1 (4.1, 8.2) |  |
| Paralytic ileus and intestine obstruction | 19358 | 19554 (19246, 19713) | -196 (-504, -37) | -0.6 (-1.5, -0.1) | -1.0 (-2.4, 0.4) |  |
| Intestine vascular disorder | 19851 | 20436 (20112, 20603) | -585 (-908, -418) | -1.8 (-2.7, -1.3) | -2.9 (-4.2, -1.5) |  |
| *C. difficile* colitis | 10469 | 8518 (8336, 8612) | 1951 (1770, 2046) | 5.9 (5.3, 6.2) | 22.9 (20.6, 25.3) |  |
| EC | 40259 | 43371 (42957, 43584) | -3112 (-3525, -2899) | -9.4 (-10.6, -8.7) | -7.2 (-8.1, -6.3) |  |
| GC | 28145 | 29848 (29510, 30023) | -1703 (-2042, -1529) | -5.1 (-6.2, -4.6) | -5.7 (-6.8, -4.6) |  |
| CRC | 137443 | 136399 (135592, 136813) | 1044 (237, 1457) | 3.1 (0.7, 4.4) | 0.8 (0.2, 1.3) |  |
| **Liver and pancreatic diseases** | | | | | |  |
| ALD | 80565 | 67356 (66818, 67632) | 13209 (12671, 13486) | 39.8 (38.2, 40.6) | 19.6 (18.8, 20.4) |  |
| Fibrosis/cirrhosis | 59010 | 55273 (54775, 55530) | 3737 (3238, 3993) | 11.3 (9.8, 12.0) | 6.8 (5.9, 7.6) |  |
| Chronic hepatitis C | 7408 | 6515 (6332, 6610) | 893 (710, 988) | 2.7 (2.1, 3.0) | 13.7 (11.1, 16.3) |  |
| Hepatic failure | 12685 | 11816 (11580, 11938) | 869 (633, 992) | 2.6 (1.9, 3.0) | 7.4 (5.5, 9.2) |  |
| LIHC | 73187 | 73830 (73253, 74127) | -643 (-1221, -347) | -1.9 (-3.7, -1.0) | -0.9 (-1.6, -0.2) |  |
| AP | 8713 | 7047 (6854, 7147) | 1666 (1474, 1767) | 5.0 (4.4, 5.3) | 23.6 (21.1, 26.3) |  |
| PC | 121885 | 125648 (124820, 126072) | -3763 (-4591, -3338) | -11.3 (-13.8, -10.1) | -3.0 (-3.5, -2.4) |  |

Abbreviations: CI, confidence interval; GI, gastrointestinal; NOS, not otherwise specified; *C. difficile*, *Clostridium difficile*; EC, esophageal cancer; GC, gastric cancer; CRC, colorectal cancer; ALD, alcohol liver disease; LIHC, liver and intrahepatic cancer; AP, acute pancreatitis; PC, pancreatic cancer.

^a^ The underlying cause of death was adopted.

^b^ Excess death number estimated by subtract the expected number from the observed number of death.

^c^ Excess mortality per 1,000,000 persons was estimated via the excess death number divided by population size.

^d^ Excess risk was calculated as the ratio of the excess-to-expected number of death.

# Table S6. Excess mortality associated with digestive related diseases from March 2020 to February 2021 (United States. 2020-2022)

| **Cause of death ^a^** | **Observed deaths, No.** | **Expected deaths**  **No. (95% CI)** | **Excess deaths**  **No. (95% CI) ^b^** | **Excess mortality**  **(95% CI) ^c^** | **Excess risk %**  **(95% CI) ^d^** |  |
| --- | --- | --- | --- | --- | --- | --- |
| **GI diseases** | | | | | |  |
| GI hemorrhage, NOS | 43771 | 34824 (34402, 35042) | 8947 (8524, 9164) | 27.0 (25.7, 27.6) | 25.7 (24.5, 26.9) |  |
| Ulcers | 8187 | 7088 (6913, 7179) | 1099 (925, 1190) | 3.3 (2.8, 3.6) | 15.5 (13.0, 18.0) |  |
| Paralytic ileus and intestine obstruction | 19248 | 19253 (18944, 19413) | -5 (-315, 155) | 0.0 (-0.9, 0.5) | 0.0 (-1.4, 1.4) |  |
| Vascular disorder of intestine | 15805 | 15926 (15642, 16073) | -121 (-406, 26) | -0.4 (-1.2, 0.1) | -0.8 (-2.3, 0.8) |  |
| *C. difficile* colitis | 7792 | 6614 (6453, 6699) | 1178 (1016, 1262) | 3.5 (3.1, 3.8) | 17.8 (15.2, 20.4) |  |
| EC | 17296 | 17983 (17703, 18128) | -687 (-967, -542) | -2.1 (-2.9, -1.6) | -3.8 (-5.2, -2.4) |  |
| GC | 12321 | 12266 (12049, 12379) | 55 (-162, 168) | 0.2 (-0.5, 0.5) | 0.4 (-1.3, 2.2) |  |
| CRC | 63079 | 61065 (60489, 61360) | 2014 (1439, 2310) | 6.1 (4.3, 7.0) | 3.3 (2.5, 4.1) |  |
| **Liver and pancreatic diseases** | | | | | |  |
| ALD | 40358 | 33115 (32750, 33303) | 7243 (6878, 7431) | 21.8 (20.7, 22.4) | 21.9 (20.7, 23.1) |  |
| Fibrosis/cirrhosis | 52015 | 47332 (46836, 47588) | 4683 (4186, 4938) | 14.1 (12.6, 14.9) | 9.9 (9.0, 10.8) |  |
| Chronic hepatitis C | 14932 | 12835 (12603, 12955) | 2097 (1865, 2217) | 6.3 (5.6, 6.7) | 16.3 (14.5, 18.2) |  |
| Hepatic failure | 30437 | 28329 (27968, 28514) | 2108 (1748, 2294) | 6.4 (5.3, 6.9) | 7.4 (6.2, 8.7) |  |
| LIHC | 31584 | 31400 (31011, 31601) | 184 (-206, 384) | 0.6 (-0.6, 1.2) | 0.6 (-0.5, 1.7) |  |
| AP | 7150 | 5800 (5642, 5882) | 1350 (1193, 1433) | 4.1 (3.6, 4.3) | 23.3 (20.4, 26.1) |  |
| PC | 49434 | 50073 (49555, 50340) | -639 (-1158, -373) | -1.9 (-3.5, -1.1) | -1.3 (-2.1, -0.4) |  |

Abbreviations: CI, confidence interval; GI, gastrointestinal; NOS, not otherwise specified; *C. difficile*, *Clostridium difficile*; EC, esophageal cancer; GC, gastric cancer; CRC, colorectal cancer; ALD, alcohol liver disease; LIHC, liver and intrahepatic cancer; AP, acute pancreatitis; PC, pancreatic cancer.

^a^ The contributing cause of death was adopted.

^b^ Excess death number estimated by subtract the expected number from the observed number of death.

^c^ Excess mortality per 1,000,000 persons was estimated via the excess death number divided by population size.

^d^ Excess risk was calculated as the ratio of the excess-to-expected number of death.

# Table S7. Excess mortality associated with digestive related diseases from March 2021 to February 2022 (United States. 2020-2022)

| **Cause of death ^a^** | **Observed deaths, No.** | **Expected deaths No. (95% CI)** | **Excess deaths**  **No. (95% CI) ^b^** | **Excess mortality**  **(95% CI) ^c^** | **Excess risk %**  **(95% CI) ^d^** |  |
| --- | --- | --- | --- | --- | --- | --- |
| **GI diseases** | - | - | - | - | - |  |
| GI hemorrhage, NOS | 45021 | 35102 (34677, 35320) | 9919 (9495, 10138) | 29.9 (28.6, 30.5) | 28.3 (27.1, 29.4) |  |
| Ulcers | 8585 | 7304 (7126, 7396) | 1281 (1104, 1374) | 3.9 (3.3, 4.1) | 17.5 (15.1, 20.0) |  |
| Paralytic ileus and intestine obstruction | 20601 | 20032 (19716, 20195) | 569 (253, 733) | 1.7 (0.8, 2.2) | 2.8 (1.4, 4.2) |  |
| Vascular disorder of intestine | 16403 | 16585 (16295, 16735) | -182 (-472, -32) | -0.5 (-1.4, -0.1) | -1.1 (-2.6, 0.4) |  |
| *C. difficile* colitis | 8247 | 5667 (5518, 5745) | 2580 (2431, 2658) | 7.8 (7.3, 8.0) | 45.5 (42.4, 48.7) |  |
| EC | 17627 | 18670 (18384, 18817) | -1043 (-1328, -895) | -3.1 (-4.0, -2.7) | -5.6 (-7.0, -4.2) |  |
| GC | 12036 | 12521 (12302, 12635) | -485 (-704, -371) | -1.5 (-2.1, -1.1) | -3.9 (-5.6, -2.1) |  |
| CRC | 63923 | 61558 (60980, 61855) | 2365 (1787, 2662) | 7.1 (5.4, 8.0) | 3.8 (3.0, 4.6) |  |
| **Liver and pancreatic diseases** | - | - | - | - | - |  |
| ALD | 43258 | 34735 (34361, 34928) | 8523 (8149, 8716) | 25.7 (24.6, 26.3) | 24.5 (23.4, 25.7) |  |
| Fibrosis/cirrhosis | 55267 | 49875 (49365, 50137) | 5392 (4883, 5654) | 16.2 (14.7, 17.0) | 10.8 (9.9, 11.7) |  |
| Chronic hepatitis C | 13746 | 11824 (11601, 11939) | 1922 (1699, 2038) | 5.8 (5.1, 6.1) | 16.3 (14.3, 18.2) |  |
| Hepatic failure | 31795 | 29044 (28679, 29232) | 2751 (2387, 2939) | 8.3 (7.2, 8.9) | 9.5 (8.3, 10.7) |  |
| LIHC | 32536 | 32138 (31744, 32341) | 398 (4, 601) | 1.2 (0.0, 1.8) | 1.2 (0.1, 2.3) |  |
| AP | 7272 | 5958 (5799, 6042) | 1314 (1154, 1397) | 4.0 (3.5, 4.2) | 22.1 (19.3, 24.9) |  |
| PC | 50931 | 51978 (51450, 52249) | -1047 (-1575, -776) | -3.2 (-4.7, -2.3) | -2.0 (-2.9, -1.2) |  |

Abbreviations: CI, confidence interval; GI, gastrointestinal; NOS, not otherwise specified; *C. difficile*, *Clostridium difficile*; EC, esophageal cancer; GC, gastric cancer; CRC, colorectal cancer; ALD, alcohol liver disease; LIHC, liver and intrahepatic cancer; AP, acute pancreatitis; PC, pancreatic cancer.

^a^ The contributing cause of death was adopted.

^b^ Excess death number estimated by subtract the expected number from the observed number of death.

^c^ Excess mortality per 1,000,000 persons was estimated via the excess death number divided by population size.

^d^ Excess risk was calculated as the ratio of the excess-to-expected number of death.

# Table S8. Excess mortality associated with digestive related diseases from March 2022 to September 2022 (United States. 2020-2022)

| **Cause of death ^a^** | **Observed deaths, No.** | **Expected deaths No. (95% CI)** | **Excess deaths**  **No. (95% CI) ^b^** | **Excess mortality**  **(95% CI) ^c^** | **Excess risk %**  **(95% CI) ^d^** |
| --- | --- | --- | --- | --- | --- |
| **GI diseases** | | | | | |
| GI hemorrhage, NOS | 22877 | 19587 (19270, 19751) | 3290 (2973, 3454) | 9.9 (9.0, 10.4) | 16.8 (15.3, 18.3) |
| Ulcers | 4538 | 4121 (3988, 4191) | 417 (284, 487) | 1.3 (0.9, 1.5) | 10.1 (6.9, 13.3) |
| Paralytic ileus and intestine obstruction | 12122 | 11682 (11441, 11807) | 440 (199, 565) | 1.3 (0.6, 1.7) | 3.8 (1.9, 5.6) |
| Vascular disorder of intestine | 9308 | 9604 (9383, 9719) | -296 (-517, -181) | -0.9 (-1.6, -0.5) | -3.1 (-5.0, -1.1) |
| *C. difficile* colitis | 4494 | 2826 (2721, 2882) | 1668 (1562, 1724) | 5.0 (4.7, 5.2) | 59.0 (54.4, 63.7) |
| EC | 10157 | 10920 (10702, 11034) | -763 (-982, -650) | -2.3 (-3.0, -2.0) | -7.0 (-8.8, -5.2) |
| GC | 6892 | 7256 (7089, 7344) | -364 (-531, -277) | -1.1 (-1.6, -0.8) | -5.0 (-7.2, -2.8) |
| CRC | 35971 | 34953 (34517, 35177) | 1018 (583, 1242) | 3.1 (1.8, 3.7) | 2.9 (1.9, 4.0) |
| **Liver and pancreatic diseases** | | | | | |
| ALD | 21996 | 20231 (19945, 20378) | 1765 (1480, 1913) | 5.3 (4.5, 5.8) | 8.7 (7.3, 10.2) |
| Fibrosis/cirrhosis | 29715 | 29049 (28660, 29250) | 666 (277, 866) | 2.0 (0.8, 2.6) | 2.3 (1.1, 3.5) |
| Chronic hepatitis C | 6862 | 6210 (6048, 6294) | 652 (491, 737) | 2.0 (1.5, 2.2) | 10.5 (7.9, 13.1) |
| Hepatic failure | 17033 | 16618 (16342, 16761) | 415 (139, 557) | 1.3 (0.4, 1.7) | 2.5 (1.0, 4.0) |
| LIHC | 18436 | 18622 (18322, 18777) | -186 (-486, -31) | -0.6 (-1.5, -0.1) | -1.0 (-2.4, 0.4) |
| AP | 3875 | 3412 (3292, 3476) | 463 (342, 526) | 1.4 (1.0, 1.6) | 13.6 (10.0, 17.2) |
| PC | 29362 | 30402 (29999, 30610) | -1040 (-1444, -832) | -3.1 (-4.4, -2.5) | -3.4 (-4.5, -2.3) |

Abbreviations: CI, confidence interval; GI, gastrointestinal; NOS, not otherwise specified; *C. difficile*, *Clostridium difficile*; EC, esophageal cancer; GC, gastric cancer; CRC, colorectal cancer; ALD, alcohol liver disease; LIHC, liver and intrahepatic cancer; AP, acute pancreatitis; PC, pancreatic cancer.

^a^ The contributing cause of death was adopted.

^b^ Excess death number estimated by subtract the expected number from the observed number of death.

^c^ Excess mortality per 1,000,000 persons was estimated via the excess death number divided by population size.

^d^ Excess risk was calculated as the ratio of the excess-to-expected number of death.

# Table S9. Excess mortality associated with selected digestive related diseases stratified by demographic factor from March 2020 to February 2021 (United States. 2020-2022)

| **Cause of death ^a^** | **Observed deaths, No.** | **Expected deaths No. (95% CI)** | **Excess deaths**  **No. (95% CI) ^b^** | **Excess mortality**  **(95% CI) ^c^** | **Excess risk % (95% CI) ^d^** |
| --- | --- | --- | --- | --- | --- |
| **Gastrointestinal diseases** | | | | | |
| **GI hemorrhage, NOS** | | | | | |
| Age |  |  |  |  |  |
| 20-64 years | 11338 | 8229 (8014, 8341) | 3109 (2893, 3221) | 16.0 (14.9, 16.6) | 37.8 (35.3, 40.3) |
| 65-84 years | 19895 | 15851 (15590, 15986) | 4044 (3783, 4179) | 86.8 (81.2, 89.7) | 25.5 (23.8, 27.3) |
| Sex |  |  |  |  |  |
| Female | 19424 | 15668 (15395, 15809) | 3756 (3484, 3897) | 22.6 (20.9, 23.4) | 24.0 (22.2, 25.7) |
| Male | 24347 | 19122 (18799, 19289) | 5225 (4901, 5392) | 32.0 (30.0, 33.0) | 27.3 (25.7, 28.9) |
| Race/ethnicity^e^ |  |  |  |  |  |
| NHW | 31563 | 26173 (25796, 26367) | 5390 (5013, 5584) | 27.5 (25.6, 28.5) | 20.6 (19.3, 21.9) |
| NHB | 5609 | 4107 (3962, 4183) | 1502 (1357, 1578) | 37.4 (33.8, 39.3) | 36.6 (33.0, 40.2) |
| Hispanic | 4177 | 2664 (2563, 2718) | 1513 (1412, 1566) | 24.9 (23.2, 25.8) | 56.8 (52.1, 61.6) |
| **Ulcers** | | | | | |
| Age |  |  |  |  |  |
| 20-64 years | 1272 | 1012 (911, 1066) | 260 (158, 313) | 1.3 (0.8, 1.6) | 25.7 (18.9, 32.7) |
| 65-84 years | 4050 | 3603 (3417, 3700) | 447 (261, 544) | 9.6 (5.6, 11.7) | 12.4 (9.0, 15.9) |
| Sex |  |  |  |  |  |
| Female | 3833 | 3400 (3281, 3462) | 433 (315, 496) | 2.6 (1.9, 3.0) | 12.7 (9.2, 16.3) |
| Male | 4354 | 3681 (3553, 3748) | 673 (545, 740) | 4.1 (3.3, 4.5) | 18.3 (14.8, 21.8) |
| Race/ethnicity^e^ |  |  |  |  |  |
| NHW | 6146 | 5405 (5261, 5480) | 741 (597, 817) | 3.8 (3.0, 4.2) | 13.7 (10.9, 16.6) |
| NHB | 813 | 676 (625, 704) | 137 (86, 165) | 3.4 (2.1, 4.1) | 20.3 (12.1, 28.7) |
| Hispanic | 555 | 506 (462, 531) | 49 (5, 73) | 0.8 (0.1, 1.2) | 9.7 (0.7, 19.0) |
| ***C. difficile* colitis** | | | | | |
| Age |  |  |  |  |  |
| 20-64 years | 492 | 369 (323, 394) | 123 (78, 148) | 0.6 (0.4, 0.8) | 33.3 (21.8, 45.4) |
| 65-84 years | 4049 | 3508 (3372, 3579) | 541 (405, 612) | 11.6 (8.7, 13.1) | 15.4 (11.9, 19.0) |
| Sex |  |  |  |  |  |
| Female | 4183 | 3596 (3476, 3659) | 587 (468, 650) | 3.5 (2.8, 3.9) | 16.3 (12.8, 19.9) |
| Male | 3609 | 3020 (2912, 3077) | 589 (481, 646) | 3.6 (2.9, 4.0) | 19.5 (15.6, 23.4) |
| Race/ethnicity^e^ |  |  |  |  |  |
| NHW | 6054 | 5156 (5005, 5236) | 898 (746, 977) | 4.6 (3.8, 5.0) | 17.4 (14.5, 20.4) |
| NHB | 791 | 617 (569, 644) | 174 (125, 201) | 4.3 (3.1, 5.0) | 28.2 (19.4, 37.3) |
| Hispanic | 509 | 508 (463, 532) | 1 (-43, 26) | 0.0 (-0.7, 0.4) | 0.2 (-8.3, 9.1) |
| **Colorectal cancer** | | | | | |
| Age |  |  |  |  |  |
| 20-64 years | 18262 | 17887 (17579, 18046) | 375 (68, 534) | 1.9 (0.4, 2.8) | 2.1 (0.6, 3.6) |
| 65-84 years | 30758 | 29390 (29026, 29578) | 1368 (1004, 1556) | 29.4 (21.6, 33.4) | 4.7 (3.5, 5.8) |
| Sex |  |  |  |  |  |
| Female | 28988 | 28359 (27972, 28559) | 629 (242, 828) | 3.8 (1.5, 5.0) | 2.2 (1.0, 3.4) |
| Male | 34091 | 32646 (32288, 32831) | 1445 (1087, 1629) | 8.9 (6.7, 10.0) | 4.4 (3.3, 5.5) |
| Race/ethnicity^e^ |  |  |  |  |  |
| NHW | 46441 | 45060 (44583, 45305) | 1381 (904, 1626) | 7.0 (4.6, 8.3) | 3.1 (2.1, 4.0) |
| NHB | 8377 | 7807 (7634, 7897) | 570 (397, 660) | 14.2 (9.9, 16.4) | 7.3 (5.0, 9.6) |
| Hispanic | 5134 | 4878 (4726, 4957) | 256 (104, 336) | 4.2 (1.7, 5.5) | 5.2 (2.4, 8.1) |
| **Liver and pancreatic diseases** | | | | | |
| **ALD** | | | | | |
| Age |  |  |  |  |  |
| 20-64 years | 29470 | 23544 (23230, 23707) | 5926 (5611, 6088) | 30.6 (28.9, 31.4) | 25.2 (23.7, 26.6) |
| 65-84 years | 10239 | 8839 (8637, 8944) | 1400 (1198, 1505) | 30.1 (25.7, 32.3) | 15.8 (13.6, 18.1) |
| Sex |  |  |  |  |  |
| Female | 12334 | 9869 (9674, 9970) | 2465 (2271, 2567) | 14.8 (13.6, 15.4) | 25.0 (22.8, 27.2) |
| Male | 28024 | 23224 (22925, 23378) | 4800 (4502, 4955) | 29.4 (27.6, 30.4) | 20.7 (19.3, 22.1) |
| Race/ethnicity^e^ |  |  |  |  |  |
| NHW | 27883 | 23459 (23159, 23614) | 4424 (4123, 4579) | 22.6 (21.0, 23.4) | 18.9 (17.5, 20.3) |
| NHB | 3229 | 2543 (2441, 2597) | 686 (584, 740) | 17.1 (14.5, 18.4) | 27.0 (22.6, 31.4) |
| Hispanic | 6429 | 5092 (4943, 5170) | 1337 (1189, 1415) | 22.0 (19.6, 23.3) | 26.3 (23.2, 29.4) |
| **Chronic hepatitis C** | | | | | |
| Age |  |  |  |  |  |
| 20-64 years | 7388 | 6467 (6298, 6555) | 921 (752, 1009) | 4.8 (3.9, 5.2) | 14.2 (11.7, 16.9) |
| 65-84 years | 5948 | 4958 (4789, 5046) | 990 (821, 1078) | 21.2 (17.6, 23.1) | 20.0 (16.9, 23.0) |
| Sex |  |  |  |  |  |
| Female | 4341 | 3568 (3451, 3629) | 773 (656, 835) | 4.6 (3.9, 5.0) | 21.7 (18.1, 25.3) |
| Male | 10591 | 9260 (9071, 9358) | 1331 (1143, 1430) | 8.2 (7.0, 8.8) | 14.4 (12.2, 16.6) |
| Race/ethnicity^e^ |  |  |  |  |  |
| NHW | 9277 | 8155 (7971, 8252) | 1122 (937, 1218) | 5.7 (4.8, 6.2) | 13.8 (11.5, 16.1) |
| NHB | 2798 | 2292 (2198, 2342) | 506 (412, 556) | 12.6 (10.2, 13.8) | 22.1 (17.6, 26.6) |
| Hispanic | 1914 | 1531 (1448, 1575) | 383 (300, 427) | 6.3 (4.9, 7.0) | 25.0 (19.5, 30.7) |
| **Fibrosis/cirrhosis** | | | | | |
| Age |  |  |  |  |  |
| 20-64 years | 21659 | 18741 (18394, 18920) | 2918 (2571, 3097) | 15.1 (13.3, 16.0) | 15.6 (14.0, 17.1) |
| 65-84 years | 26113 | 24411 (24091, 24576) | 1702 (1383, 1868) | 36.5 (29.7, 40.1) | 7.0 (5.7, 8.3) |
| Sex |  |  |  |  |  |
| Female | 21749 | 19800 (19514, 19947) | 1949 (1664, 2097) | 11.7 (10.0, 12.6) | 9.8 (8.4, 11.3) |
| Male | 30266 | 27489 (27156, 27661) | 2777 (2444, 2949) | 17.0 (15.0, 18.1) | 10.1 (8.9, 11.3) |
| Race/ethnicity^e^ |  |  |  |  |  |
| NHW | 36387 | 33900 (33507, 34103) | 2487 (2093, 2690) | 12.7 (10.7, 13.7) | 7.3 (6.2, 8.4) |
| NHB | 4749 | 4236 (4096, 4310) | 513 (373, 586) | 12.8 (9.3, 14.6) | 12.1 (8.9, 15.3) |
| Hispanic | 8026 | 6462 (6296, 6548) | 1564 (1399, 1651) | 25.7 (23.0, 27.2) | 24.2 (21.5, 26.9) |
| **Hepatic failure** | | | | | |
| Age |  |  |  |  |  |
| 20-64 years | 15128 | 13486 (13228, 13620) | 1642 (1383, 1776) | 8.5 (7.1, 9.2) | 12.2 (10.4, 14.0) |
| 65-84 years | 12805 | 12303 (12065, 12426) | 502 (264, 625) | 10.8 (5.7, 13.4) | 4.1 (2.3, 5.9) |
| Sex |  |  |  |  |  |
| Female | 13255 | 12512 (12293, 12626) | 743 (524, 857) | 4.5 (3.1, 5.1) | 5.9 (4.1, 7.7) |
| Male | 17182 | 15792 (15542, 15921) | 1390 (1140, 1520) | 8.5 (7.0, 9.3) | 8.8 (7.2, 10.4) |
| Race/ethnicity^e^ |  |  |  |  |  |
| NHW | 21004 | 19872 (19591, 20017) | 1132 (851, 1278) | 5.8 (4.3, 6.5) | 5.7 (4.3, 7.1) |
| NHB | 3466 | 3137 (3027, 3195) | 329 (219, 387) | 8.2 (5.4, 9.6) | 10.5 (6.8, 14.2) |
| Hispanic | 4045 | 3491 (3375, 3552) | 554 (439, 616) | 9.1 (7.2, 10.1) | 15.9 (12.3, 19.5) |
| **Acute pancreatitis** | | | | | |
| Age |  |  |  |  |  |
| 20-64 years | 2094 | 1518 (1378, 1592) | 576 (435, 649) | 3.0 (2.2, 3.3) | 37.9 (32.1, 43.9) |
| 65-84 years | 2274 | 1671 (1497, 1761) | 603 (430, 694) | 12.9 (9.2, 14.9) | 36.1 (30.6, 41.7) |
| Sex |  |  |  |  |  |
| Female | 2806 | 2311 (2210, 2364) | 495 (394, 549) | 3.0 (2.4, 3.3) | 21.4 (17.0, 26.0) |
| Male | 4344 | 3485 (3354, 3553) | 859 (729, 928) | 5.3 (4.5, 5.7) | 24.6 (21.0, 28.4) |
| Race/ethnicity^e^ |  |  |  |  |  |
| NHW | 5109 | 4188 (4053, 4259) | 921 (785, 992) | 4.7 (4.0, 5.1) | 22.0 (18.7, 25.4) |
| NHB | 994 | 766 (712, 795) | 228 (174, 258) | 5.7 (4.3, 6.4) | 29.8 (21.8, 38.0) |
| Hispanic | 635 | 605 (556, 631) | 30 (-18, 57) | 0.5 (-0.3, 0.9) | 5.0 (-3.0, 13.3) |

Abbreviations: CI, confidence interval; GI, gastrointestinal; NOS, not otherwise specified; *C. difficile*, *Clostridium difficile*; ALD, alcohol liver disease; NHW, non-Hispanic White inhabitants; NHB, non-Hispanic Black inhabitants.

^a^ The contributing cause of death was adopted.

^b^ Excess death number estimated by subtract the expected number from the observed number of death.

^c^ Excess mortality per 1,000,000 persons was estimated via the excess death number divided by population size.

^d^ Excess risk was calculated as the ratio of the excess-to-expected number of death.

^e^ Non-Hispanic unknown and non-Hispanic AIAN was excluded when stratified by race/ethnicity.

# Table S10. Excess mortality associated with selected digestive related diseases stratified by demographic factor from March 2021 to February 2022 (United States. 2020-2022)

| **Cause of death ^a^** | **Observed deaths, No.** | **Expected deaths No. (95% CI)** | **Excess deaths**  **No. (95% CI) ^b^** | **Excess mortality**  **(95% CI) ^c^** | **Excess risk % (95% CI) ^d^** |
| --- | --- | --- | --- | --- | --- |
| **Gastrointestinal diseases** | | | | | |
| **GI hemorrhage, NOS** | | | | | |
| Age |  |  |  |  |  |
| 20-64 years | 12229 | 8242 (8027, 8354) | 3987 (3771, 4099) | 20.6 (19.5, 21.1) | 48.4 (45.8, 51.0) |
| 65-84 years | 20833 | 16038 (15776, 16174) | 4795 (4532, 4931) | 102.9 (97.3, 105.8) | 29.9 (28.1, 31.7) |
| Sex |  |  |  |  |  |
| Female | 19867 | 15540 (15269, 15680) | 4327 (4056, 4468) | 26.0 (24.4, 26.8) | 27.8 (26.1, 29.6) |
| Male | 25154 | 19356 (19031, 19524) | 5798 (5473, 5966) | 35.5 (33.5, 36.6) | 30.0 (28.4, 31.6) |
| Race/ethnicity^e^ |  |  |  |  |  |
| NHW | 32938 | 26125 (25749, 26319) | 6813 (6436, 7007) | 34.8 (32.8, 35.7) | 26.1 (24.7, 27.4) |
| NHB | 5523 | 4125 (3979, 4201) | 1398 (1252, 1474) | 34.8 (31.1, 36.7) | 33.9 (30.4, 37.4) |
| Hispanic | 4089 | 2651 (2550, 2705) | 1438 (1337, 1491) | 23.6 (22.0, 24.5) | 54.2 (49.6, 59.0) |
| **Ulcers** | - | - | - | - | - |
| Age |  |  |  |  |  |
| 20-64 years | 1287 | 1004 (903, 1058) | 283 (182, 336) | 1.5 (0.9, 1.7) | 28.2 (21.3, 35.3) |
| 65-84 years | 4314 | 3889 (3696, 3989) | 425 (232, 525) | 9.1 (5.0, 11.3) | 10.9 (7.6, 14.3) |
| Sex |  |  |  |  |  |
| Female | 3997 | 3521 (3401, 3585) | 476 (355, 539) | 2.9 (2.1, 3.2) | 13.5 (10.0, 17.1) |
| Male | 4588 | 3739 (3610, 3807) | 849 (720, 917) | 5.2 (4.4, 5.6) | 22.7 (19.2, 26.3) |
| Race/ethnicity^e^ |  |  |  |  |  |
| NHW | 6333 | 5501 (5355, 5577) | 832 (687, 909) | 4.2 (3.5, 4.6) | 15.1 (12.3, 18.0) |
| NHB | 936 | 733 (680, 762) | 203 (150, 232) | 5.1 (3.7, 5.8) | 27.7 (19.6, 36.0) |
| Hispanic | 718 | 627 (578, 654) | 91 (42, 118) | 1.5 (0.7, 1.9) | 14.5 (6.3, 23.0) |
| ***C. difficile* colitis** | - | - | - | - | - |
| Age |  |  |  |  |  |
| 20-64 years | 518 | 320 (278, 344) | 198 (156, 221) | 1.0 (0.8, 1.1) | 61.9 (48.2, 76.1) |
| 65-84 years | 4620 | 3000 (2874, 3066) | 1620 (1495, 1686) | 34.8 (32.1, 36.2) | 54.0 (49.6, 58.5) |
| Sex |  |  |  |  |  |
| Female | 4411 | 2969 (2860, 3026) | 1442 (1334, 1500) | 8.7 (8.0, 9.0) | 48.6 (44.2, 53.0) |
| Male | 3836 | 2681 (2579, 2735) | 1155 (1054, 1209) | 7.1 (6.5, 7.4) | 43.1 (38.6, 47.6) |
| Race/ethnicity^e^ |  |  |  |  |  |
| NHW | 6470 | 4341 (4202, 4414) | 2129 (1990, 2202) | 10.9 (10.2, 11.2) | 49.0 (45.4, 52.7) |
| NHB | 809 | 519 (474, 543) | 290 (246, 315) | 7.2 (6.1, 7.8) | 55.9 (45.3, 66.8) |
| Hispanic | 501 | 504 (460, 529) | -3 (-47, 21) | 0.0 (-0.8, 0.3) | -0.6 (-9.1, 8.3) |
| **Colorectal cancer** | - | - | - | - | - |
| Age |  |  |  |  |  |
| 20-64 years | 18618 | 18148 (17838, 18308) | 470 (160, 630) | 2.4 (0.8, 3.3) | 2.6 (1.1, 4.1) |
| 65-84 years | 31836 | 28953 (28591, 29139) | 2883 (2522, 3069) | 61.9 (54.1, 65.9) | 10.0 (8.8, 11.2) |
| Sex |  |  |  |  |  |
| Female | 29447 | 28275 (27889, 28475) | 1172 (785, 1371) | 7.0 (4.7, 8.2) | 4.1 (3.0, 5.3) |
| Male | 34476 | 32907 (32547, 33092) | 1569 (1210, 1755) | 9.6 (7.4, 10.8) | 4.8 (3.7, 5.9) |
| Race/ethnicity^e^ |  |  |  |  |  |
| NHW | 47012 | 44813 (44337, 45057) | 2199 (1724, 2444) | 11.2 (8.8, 12.5) | 4.9 (4.0, 5.9) |
| NHB | 8234 | 7819 (7646, 7910) | 415 (241, 505) | 10.3 (6.0, 12.6) | 5.3 (3.0, 7.6) |
| Hispanic | 5320 | 5014 (4860, 5095) | 306 (152, 386) | 5.0 (2.5, 6.3) | 6.1 (3.3, 9.0) |
| **Liver and pancreatic diseases** | | | | | |
| **ALD** | - | - | - | - | - |
| Age |  |  |  |  |  |
| 20-64 years | 31526 | 24438 (24117, 24603) | 7088 (6768, 7254) | 36.6 (34.9, 37.4) | 29.0 (27.6, 30.4) |
| 65-84 years | 11048 | 9339 (9132, 9447) | 1709 (1501, 1816) | 36.7 (32.2, 39.0) | 18.3 (16.1, 20.5) |
| Sex |  |  |  |  |  |
| Female | 13056 | 10388 (10188, 10492) | 2668 (2468, 2772) | 16.0 (14.8, 16.6) | 25.7 (23.5, 27.8) |
| Male | 30202 | 24197 (23892, 24354) | 6005 (5700, 6163) | 36.8 (34.9, 37.8) | 24.8 (23.4, 26.2) |
| Race/ethnicity^e^ |  |  |  |  |  |
| NHW | 29720 | 24537 (24229, 24695) | 5183 (4876, 5342) | 26.4 (24.9, 27.3) | 21.1 (19.8, 22.5) |
| NHB | 3576 | 2606 (2502, 2661) | 970 (867, 1025) | 24.1 (21.6, 25.5) | 37.2 (32.8, 41.8) |
| Hispanic | 6725 | 5338 (5186, 5417) | 1387 (1235, 1467) | 22.8 (20.3, 24.1) | 26.0 (23.0, 29.0) |
| **Chronic hepatitis C** | - | - | - | - | - |
| Age |  |  |  |  |  |
| 20-64 years | 6537 | 5532 (5376, 5614) | 1005 (849, 1087) | 5.2 (4.4, 5.6) | 18.2 (15.3, 21.0) |
| 65-84 years | 5658 | 4919 (4750, 5006) | 739 (571, 827) | 15.9 (12.3, 17.8) | 15.0 (12.0, 18.0) |
| Sex |  |  |  |  |  |
| Female | 4049 | 3225 (3113, 3283) | 824 (713, 883) | 4.9 (4.3, 5.3) | 25.6 (21.7, 29.4) |
| Male | 9697 | 8551 (8370, 8645) | 1146 (965, 1241) | 7.0 (5.9, 7.6) | 13.4 (11.2, 15.7) |
| Race/ethnicity^e^ |  |  |  |  |  |
| NHW | 8752 | 7530 (7352, 7622) | 1222 (1045, 1315) | 6.2 (5.3, 6.7) | 16.2 (13.8, 18.7) |
| NHB | 2354 | 2046 (1957, 2093) | 308 (219, 355) | 7.7 (5.4, 8.8) | 15.1 (10.5, 19.7) |
| Hispanic | 1774 | 1334 (1257, 1376) | 440 (362, 481) | 7.2 (6.0, 7.9) | 33.0 (26.9, 39.2) |
| **Fibrosis/cirrhosis** | - | - | - | - | - |
| Age |  |  |  |  |  |
| 20-64 years | 22872 | 18805 (18457, 18984) | 4067 (3720, 4246) | 21.0 (19.2, 21.9) | 21.6 (20.1, 23.2) |
| 65-84 years | 28138 | 26015 (25684, 26185) | 2123 (1793, 2294) | 45.6 (38.5, 49.2) | 8.2 (6.9, 9.4) |
| Sex |  |  |  |  |  |
| Female | 23628 | 20885 (20591, 21036) | 2743 (2450, 2895) | 16.5 (14.7, 17.4) | 13.1 (11.7, 14.6) |
| Male | 31639 | 28714 (28373, 28889) | 2925 (2585, 3101) | 17.9 (15.8, 19.0) | 10.2 (9.0, 11.4) |
| Race/ethnicity^e^ |  |  |  |  |  |
| NHW | 39376 | 35631 (35227, 35839) | 3745 (3342, 3953) | 19.1 (17.1, 20.2) | 10.5 (9.4, 11.6) |
| NHB | 4757 | 4405 (4262, 4479) | 352 (210, 427) | 8.8 (5.2, 10.6) | 8.0 (4.9, 11.1) |
| Hispanic | 8071 | 6535 (6369, 6622) | 1536 (1369, 1623) | 25.3 (22.5, 26.7) | 23.5 (20.8, 26.2) |
| **Hepatic failure** | - | - | - | - | - |
| Age |  |  |  |  |  |
| 20-64 years | 16197 | 13670 (13410, 13805) | 2527 (2266, 2662) | 13.0 (11.7, 13.7) | 18.5 (16.7, 20.3) |
| 65-84 years | 13273 | 12571 (12330, 12695) | 702 (462, 827) | 15.1 (9.9, 17.8) | 5.6 (3.8, 7.4) |
| Sex |  |  |  |  |  |
| Female | 13994 | 12903 (12681, 13019) | 1091 (868, 1206) | 6.6 (5.2, 7.2) | 8.5 (6.7, 10.3) |
| Male | 17801 | 15979 (15728, 16110) | 1822 (1570, 1952) | 11.2 (9.6, 12.0) | 11.4 (9.8, 13.0) |
| Race/ethnicity^e^ |  |  |  |  |  |
| NHW | 22155 | 20128 (19846, 20275) | 2027 (1744, 2173) | 10.3 (8.9, 11.1) | 10.1 (8.6, 11.5) |
| NHB | 3526 | 3191 (3080, 3249) | 335 (224, 394) | 8.3 (5.6, 9.8) | 10.5 (6.9, 14.2) |
| Hispanic | 4070 | 3593 (3475, 3655) | 477 (360, 539) | 7.8 (5.9, 8.9) | 13.3 (9.8, 16.8) |
| **Acute pancreatitis** | - | - | - | - | - |
| Age |  |  |  |  |  |
| 20-64 years | 2178 | 1740 (1589, 1819) | 438 (287, 517) | 2.3 (1.5, 2.7) | 25.2 (20.0, 30.5) |
| 65-84 years | 2361 | 1659 (1486, 1749) | 702 (529, 792) | 15.1 (11.4, 17.0) | 42.3 (36.6, 48.1) |
| Sex |  |  |  |  |  |
| Female | 2913 | 2315 (2214, 2368) | 598 (497, 652) | 3.6 (3.0, 3.9) | 25.8 (21.3, 30.4) |
| Male | 4359 | 3615 (3483, 3685) | 744 (611, 813) | 4.6 (3.7, 5.0) | 20.6 (17.0, 24.2) |
| Race/ethnicity^e^ |  |  |  |  |  |
| NHW | 5248 | 4213 (4077, 4284) | 1035 (900, 1106) | 5.3 (4.6, 5.6) | 24.6 (21.2, 28.0) |
| NHB | 891 | 755 (701, 784) | 136 (82, 166) | 3.4 (2.0, 4.1) | 18.0 (10.4, 25.9) |
| Hispanic | 644 | 686 (635, 714) | -42 (-93, -14) | -0.7 (-1.5, -0.2) | -6.1 (-13.2, 1.3) |

Abbreviations: CI, confidence interval; GI, gastrointestinal; NOS, not otherwise specified; *C. difficile*, *Clostridium difficile*; ALD, alcohol liver disease; NHW, non-Hispanic White inhabitants; NHB, non-Hispanic Black inhabitants.

^a^ The contributing cause of death was adopted.

^b^ Excess death number estimated by subtract the expected number from the observed number of death.

^c^ Excess mortality per 1,000,000 persons was estimated via the excess death number divided by population size.

^d^ Excess risk was calculated as the ratio of the excess-to-expected number of death.

^e^ Non-Hispanic unknown and non-Hispanic AIAN was excluded when stratified by race/ethnicity.

# Table S11. Excess mortality associated with selected digestive related diseases stratified by demographic from March 2022 to September 2022 (United States. 2020-2022)

| **Cause of death ^a^** | **Observed deaths, No.** | **Expected deaths No. (95% CI)** | **Excess deaths**  **No. (95% CI) ^b^** | **Excess mortality**  **(95% CI) ^c^** | **Excess risk % (95% CI) ^d^** |
| --- | --- | --- | --- | --- | --- |
| **Gastrointestinal diseases** | | | | | |
| **GI hemorrhage, NOS** | | | | | |
| Age |  |  |  |  |  |
| 20-64 years | 5647 | 4593 (4432, 4678) | 1054 (893, 1138) | 5.4 (4.6, 5.9) | 22.9 (19.8, 26.2) |
| 65-84 years | 10827 | 8972 (8776, 9074) | 1855 (1658, 1957) | 39.8 (35.6, 42.0) | 20.7 (18.4, 23.0) |
| Sex |  |  |  |  |  |
| Female | 10410 | 8649 (8446, 8754) | 1761 (1559, 1866) | 10.6 (9.4, 11.2) | 20.4 (18.1, 22.7) |
| Male | 12467 | 10824 (10580, 10950) | 1643 (1400, 1770) | 10.1 (8.6, 10.8) | 15.2 (13.2, 17.2) |
| Race/ethnicity^e^ |  |  |  |  |  |
| NHW | 16813 | 14664 (14382, 14810) | 2149 (1867, 2295) | 11.0 (9.5, 11.7) | 14.7 (12.9, 16.4) |
| NHB | 2743 | 2293 (2184, 2350) | 450 (342, 507) | 11.2 (8.5, 12.6) | 19.6 (15.2, 24.1) |
| Hispanic | 2056 | 1444 (1370, 1484) | 612 (537, 652) | 10.1 (8.8, 10.7) | 42.4 (36.3, 48.6) |
| **Ulcers** | - | - | - | - | - |
| Age |  |  |  |  |  |
| 20-64 years | 597 | 540 (466, 580) | 57 (-17, 96) | 0.3 (-0.1, 0.5) | 10.6 (1.9, 19.6) |
| 65-84 years | 2341 | 2260 (2113, 2337) | 81 (-66, 158) | 1.7 (-1.4, 3.4) | 3.6 (-0.6, 7.8) |
| Sex |  |  |  |  |  |
| Female | 2146 | 1997 (1906, 2045) | 149 (59, 197) | 0.9 (0.4, 1.2) | 7.5 (3.0, 12.1) |
| Male | 2392 | 2100 (2003, 2151) | 292 (195, 343) | 1.8 (1.2, 2.1) | 13.9 (9.4, 18.5) |
| Race/ethnicity^e^ |  |  |  |  |  |
| NHW | 3372 | 3122 (3012, 3180) | 250 (141, 308) | 1.3 (0.7, 1.6) | 8.0 (4.4, 11.7) |
| NHB | 467 | 397 (358, 418) | 70 (31, 92) | 1.7 (0.8, 2.3) | 17.6 (7.2, 28.5) |
| Hispanic | 403 | 361 (324, 382) | 42 (5, 63) | 0.7 (0.1, 1.0) | 11.6 (1.0, 22.8) |
| ***C. difficile* colitis** | - | - | - | - | - |
| Age |  |  |  |  |  |
| 20-64 years | 240 | 119 (94, 135) | 121 (95, 136) | 0.6 (0.5, 0.7) | 101.7 (77.0, 128.0) |
| 65-84 years | 2508 | 1496 (1408, 1544) | 1012 (923, 1059) | 21.7 (19.8, 22.7) | 67.6 (61.1, 74.3) |
| Sex |  |  |  |  |  |
| Female | 2517 | 1434 (1358, 1474) | 1083 (1008, 1124) | 6.5 (6.1, 6.7) | 75.5 (68.7, 82.4) |
| Male | 1977 | 1395 (1322, 1435) | 582 (509, 621) | 3.6 (3.1, 3.8) | 41.7 (35.5, 48.0) |
| Race/ethnicity^e^ |  |  |  |  |  |
| NHW | 3475 | 2161 (2063, 2213) | 1314 (1216, 1366) | 6.7 (6.2, 7.0) | 60.8 (55.5, 66.2) |
| NHB | 481 | 259 (227, 277) | 222 (191, 240) | 5.5 (4.8, 6.0) | 85.7 (69.5, 102.7) |
| Hispanic | 273 | 273 (241, 292) | 0 (-33, 18) | 0.0 (-0.5, 0.3) | 0.0 (-11.5, 12.2) |
| **Colorectal cancer** | | | | | |
| Age |  |  |  |  |  |
| 20-64 years | 10609 | 10469 (10234, 10591) | 140 (-96, 262) | 0.7 (-0.5, 1.4) | 1.3 (-0.6, 3.3) |
| 65-84 years | 17844 | 16119 (15849, 16259) | 1725 (1455, 1864) | 37.0 (31.2, 40.0) | 10.7 (9.1, 12.3) |
| Sex |  |  |  |  |  |
| Female | 16714 | 16014 (15723, 16165) | 700 (409, 850) | 4.2 (2.5, 5.1) | 4.4 (2.8, 6.0) |
| Male | 19257 | 18725 (18454, 18866) | 532 (261, 672) | 3.3 (1.6, 4.1) | 2.8 (1.4, 4.3) |
| Race/ethnicity^e^ |  |  |  |  |  |
| NHW | 26457 | 25469 (25111, 25654) | 988 (629, 1172) | 5.0 (3.2, 6.0) | 3.9 (2.6, 5.1) |
| NHB | 4525 | 4431 (4300, 4499) | 94 (-36, 163) | 2.3 (-0.9, 4.1) | 2.1 (-0.8, 5.1) |
| Hispanic | 3073 | 2885 (2768, 2946) | 188 (72, 250) | 3.1 (1.2, 4.1) | 6.5 (2.8, 10.3) |
| **Liver and pancreatic diseases** | | | | | |
| **ALD** | - | - | - | - | - |
| Age |  |  |  |  |  |
| 20-64 years | 15515 | 14231 (13986, 14358) | 1284 (1039, 1411) | 6.6 (5.4, 7.3) | 9.0 (7.3, 10.7) |
| 65-84 years | 6061 | 5421 (5262, 5503) | 640 (482, 723) | 13.7 (10.3, 15.5) | 11.8 (9.0, 14.6) |
| Sex |  |  |  |  |  |
| Female | 6773 | 6068 (5915, 6147) | 705 (553, 785) | 4.2 (3.3, 4.7) | 11.6 (9.0, 14.3) |
| Male | 15223 | 14075 (13843, 14196) | 1148 (915, 1268) | 7.0 (5.6, 7.8) | 8.2 (6.4, 9.9) |
| Race/ethnicity^e^ |  |  |  |  |  |
| NHW | 15320 | 14456 (14220, 14578) | 864 (628, 986) | 4.4 (3.2, 5.0) | 6.0 (4.3, 7.7) |
| NHB | 1667 | 1503 (1424, 1545) | 164 (85, 206) | 4.1 (2.1, 5.1) | 10.9 (5.7, 16.3) |
| Hispanic | 3515 | 3054 (2939, 3114) | 461 (347, 522) | 7.6 (5.7, 8.6) | 15.1 (11.3, 18.9) |
| **Chronic hepatitis C** | | | | | |
| Age |  |  |  |  |  |
| 20-64 years | 2824 | 2764 (2654, 2822) | 60 (-50, 118) | 0.3 (-0.3, 0.6) | 2.2 (-1.6, 6.0) |
| 65-84 years | 3187 | 2696 (2572, 2762) | 491 (366, 556) | 10.5 (7.9, 11.9) | 18.2 (14.1, 22.4) |
| Sex |  |  |  |  |  |
| Female | 2046 | 1653 (1574, 1696) | 393 (313, 435) | 2.4 (1.9, 2.6) | 23.8 (18.5, 29.2) |
| Male | 4816 | 4533 (4401, 4602) | 283 (151, 352) | 1.7 (0.9, 2.2) | 6.2 (3.3, 9.3) |
| Race/ethnicity^e^ |  |  |  |  |  |
| NHW | 4424 | 4003 (3873, 4071) | 421 (292, 489) | 2.1 (1.5, 2.5) | 10.5 (7.3, 13.8) |
| NHB | 1241 | 1060 (996, 1095) | 181 (117, 215) | 4.5 (2.9, 5.3) | 17.1 (10.7, 23.7) |
| Hispanic | 800 | 665 (610, 695) | 135 (80, 165) | 2.2 (1.3, 2.7) | 20.3 (12.1, 28.8) |
| **Fibrosis/cirrhosis** | | | | | |
| Age |  |  |  |  |  |
| 20-64 years | 11260 | 10556 (10296, 10691) | 704 (444, 839) | 3.6 (2.3, 4.3) | 6.7 (4.7, 8.6) |
| 65-84 years | 15954 | 15283 (15029, 15414) | 671 (418, 803) | 14.4 (9.0, 17.2) | 4.4 (2.8, 6.0) |
| Sex |  |  |  |  |  |
| Female | 12813 | 12228 (12004, 12345) | 585 (360, 701) | 3.5 (2.2, 4.2) | 4.8 (3.0, 6.6) |
| Male | 16902 | 16661 (16402, 16795) | 241 (-18, 375) | 1.5 (-0.1, 2.3) | 1.4 (-0.1, 3.0) |
| Race/ethnicity^e^ |  |  |  |  |  |
| NHW | 21265 | 20962 (20652, 21122) | 303 (-6, 463) | 1.5 (0.0, 2.4) | 1.4 (0.1, 2.8) |
| NHB | 2459 | 2556 (2447, 2614) | -97 (-206, -40) | -2.4 (-5.1, -1.0) | -3.8 (-7.6, 0.0) |
| Hispanic | 4479 | 3650 (3525, 3715) | 829 (705, 895) | 13.6 (11.6, 14.7) | 22.7 (19.1, 26.3) |
| **Hepatic failure** | - | - | - | - | - |
| Age |  |  |  |  |  |
| 20-64 years | 8149 | 7766 (7570, 7868) | 383 (186, 485) | 2.0 (1.0, 2.5) | 4.9 (2.7, 7.2) |
| 65-84 years | 7448 | 7183 (7001, 7277) | 265 (84, 360) | 5.7 (1.8, 7.7) | 3.7 (1.3, 6.1) |
| Sex |  |  |  |  |  |
| Female | 7604 | 7456 (7287, 7545) | 148 (-22, 236) | 0.9 (-0.1, 1.4) | 2.0 (-0.3, 4.3) |
| Male | 9429 | 9073 (8884, 9172) | 356 (167, 455) | 2.2 (1.0, 2.8) | 3.9 (1.8, 6.0) |
| Race/ethnicity^e^ |  |  |  |  |  |
| NHW | 11820 | 11539 (11325, 11650) | 281 (67, 392) | 1.4 (0.3, 2.0) | 2.4 (0.6, 4.3) |
| NHB | 1889 | 1824 (1740, 1868) | 65 (-18, 110) | 1.6 (-0.4, 2.7) | 3.6 (-1.1, 8.3) |
| Hispanic | 2228 | 2037 (1949, 2084) | 191 (102, 238) | 3.1 (1.7, 3.9) | 9.4 (4.9, 14.0) |
| **Acute pancreatitis** | | | | | |
| Age |  |  |  |  |  |
| 20-64 years | 902 | 1065 (947, 1127) | -163 (-281, -101) | -0.8 (-1.4, -0.5) | -15.3 (-20.7, -9.7) |
| 65-84 years | 1443 | 879 (754, 946) | 564 (438, 630) | 12.1 (9.4, 13.5) | 64.2 (55.8, 72.7) |
| Sex |  |  |  |  |  |
| Female | 1604 | 1319 (1243, 1360) | 285 (209, 326) | 1.7 (1.3, 2.0) | 21.6 (15.7, 27.6) |
| Male | 2271 | 2078 (1977, 2131) | 193 (92, 246) | 1.2 (0.6, 1.5) | 9.3 (4.8, 13.8) |
| Race/ethnicity^e^ |  |  |  |  |  |
| NHW | 2809 | 2392 (2290, 2446) | 417 (314, 471) | 2.1 (1.6, 2.4) | 17.4 (13.1, 21.8) |
| NHB | 497 | 450 (409, 473) | 47 (5, 70) | 1.2 (0.1, 1.7) | 10.4 (0.9, 20.4) |
| Hispanic | 303 | 375 (337, 396) | -72 (-110, -51) | -1.2 (-1.8, -0.8) | -19.2 (-28.0, -9.8) |

Abbreviations: CI, confidence interval; GI, gastrointestinal; NOS, not otherwise specified; *C. difficile*, *Clostridium difficile*; ALD, alcohol liver disease; NHW, non-Hispanic White inhabitants; NHB, non-Hispanic Black inhabitants.

^a^ The contributing cause of death was adopted.

^b^ Excess death number estimated by subtract the expected number from the observed number of death.

^c^ Excess mortality per 1,000,000 persons was estimated via the excess death number divided by population size.

^d^ Excess risk was calculated as the ratio of the excess-to-expected number of death.

^e^ Non-Hispanic unknown and non-Hispanic AIAN was excluded when stratified by race/ethnicity.

# Table S12. The estimated excess mortality of gastrointestinal hemorrhage by state from March 2020 to September 2022 (United States. 2020-2022)

| **State** | **Abbreviation** | **Observed deaths, No..** | **Expected deaths,**  **No. (95% CI)** | **Excess deaths,**  **No. (95% CI) ^a^** | **Excess mortality**  **(95% CI) ^b^** | **Excess risk % (95% CI) ^c^** |
| --- | --- | --- | --- | --- | --- | --- |
| Alabama | AL | 1528 | 1385 (1312, 1425) | 143 (70, 182) | 28.6 (14.0, 36.4) | 10.3 (4.9, 15.9) |
| Arkansas | AR | 795 | 594 (546, 620) | 201 (153, 227) | 66.9 (50.9, 75.5) | 33.8 (24.7, 43.3) |
| Arizona | AZ | 2069 | 1469 (1394, 1510) | 600 (524, 640) | 84.8 (74.0, 90.4) | 40.8 (34.8, 47.0) |
| California | CA | 10265 | 8271 (8086, 8367) | 1994 (1809, 2091) | 50.5 (45.8, 53.0) | 24.1 (21.7, 26.5) |
| Colorado | CO | 2299 | 1822 (1739, 1867) | 477 (393, 521) | 83.3 (68.7, 91.0) | 26.2 (21.1, 31.4) |
| Connecticut | CT | 798 | 787 (732, 817) | 11 (-44, 41) | 3.1 (-12.2, 11.4) | 1.4 (-5.5, 8.6) |
| Florida | FL | 7704 | 6528 (6370, 6611) | 1176 (1017, 1259) | 55.1 (47.7, 59.0) | 18.0 (15.4, 20.7) |
| Georgia | GA | 3033 | 2410 (2312, 2462) | 623 (525, 675) | 58.6 (49.4, 63.5) | 25.9 (21.4, 30.4) |
| Iowa | IA | 827 | 947 (887, 980) | -120 (-180, -87) | -37.7 (-56.6, -27.4) | -12.7 (-18.5, -6.6) |
| Illinois | IL | 3416 | 2689 (2577, 2748) | 727 (616, 786) | 56.7 (48.0, 61.3) | 27.0 (22.8, 31.3) |
| Indiana | IN | 2684 | 2495 (2397, 2547) | 189 (91, 241) | 28.0 (13.5, 35.7) | 7.6 (3.5, 11.7) |
| Kansas | KS | 504 | 574 (527, 600) | -70 (-117, -44) | -23.9 (-39.9, -15.0) | -12.2 (-19.7, -4.4) |
| Kentucky | KY | 2215 | 2093 (2003, 2140) | 122 (33, 170) | 27.1 (7.3, 37.8) | 5.8 (1.5, 10.3) |
| Louisiana | LA | 916 | 924 (864, 956) | -8 (-68, 24) | -1.7 (-14.6, 5.2) | -0.9 (-7.2, 5.7) |
| Massachusetts | MA | 2307 | 2249 (2156, 2299) | 58 (-35, 107) | 8.3 (-5.0, 15.3) | 2.6 (-1.6, 6.8) |
| Maryland | MD | 2356 | 1712 (1631, 1755) | 644 (563, 688) | 104.7 (91.6, 111.9) | 37.6 (32.1, 43.2) |
| Michigan | MI | 3505 | 3247 (3129, 3309) | 258 (140, 320) | 25.6 (13.9, 31.8) | 7.9 (4.4, 11.5) |
| Minnesota | MN | 1802 | 1555 (1478, 1597) | 247 (169, 288) | 43.6 (29.8, 50.8) | 15.9 (10.6, 21.3) |
| Missouri | MO | 2065 | 1383 (1310, 1422) | 682 (609, 721) | 111.0 (99.2, 117.4) | 49.3 (42.9, 55.8) |
| Mississippi | MS | 753 | 614 (566, 641) | 139 (90, 166) | 46.8 (30.3, 55.9) | 22.6 (14.0, 31.6) |
| North Carolina | NC | 3397 | 3028 (2920, 3085) | 369 (261, 426) | 35.6 (25.2, 41.1) | 12.2 (8.4, 16.0) |
| New Jersey | NJ | 3270 | 2703 (2601, 2757) | 567 (465, 621) | 61.4 (50.4, 67.3) | 21.0 (16.9, 25.2) |
| New Mexico | NM | 385 | 382 (343, 403) | 3 (-35, 25) | 1.4 (-16.6, 11.9) | 0.8 (-9.0, 11.1) |
| New York | NY | 5440 | 4393 (4248, 4469) | 1047 (902, 1123) | 52.1 (44.8, 55.8) | 23.8 (20.6, 27.1) |
| Ohio | OH | 4207 | 3219 (3096, 3284) | 988 (865, 1053) | 83.9 (73.5, 89.5) | 30.7 (26.8, 34.7) |
| Oklahoma | OK | 1531 | 1053 (989, 1087) | 478 (415, 513) | 121.1 (105.1, 129.9) | 45.4 (38.2, 52.8) |
| Oregon | OR | 1337 | 1109 (1044, 1145) | 228 (162, 263) | 54.2 (38.5, 62.5) | 20.6 (14.2, 27.1) |
| Pennsylvania | PA | 5350 | 4124 (3991, 4194) | 1226 (1093, 1296) | 94.5 (84.3, 99.9) | 29.7 (26.3, 33.2) |
| South Carolina | SC | 2396 | 2027 (1939, 2074) | 369 (280, 416) | 72.7 (55.1, 81.9) | 18.2 (13.5, 23.0) |
| Tennessee | TN | 2797 | 2573 (2474, 2626) | 224 (124, 277) | 32.7 (18.1, 40.4) | 8.7 (4.7, 12.8) |
| Texas | TX | 8782 | 6257 (6102, 6338) | 2525 (2370, 2606) | 87.5 (82.1, 90.3) | 40.4 (37.4, 43.3) |
| Virginia | VA | 2467 | 1834 (1750, 1879) | 633 (549, 678) | 73.8 (64.0, 79.0) | 34.5 (29.3, 39.9) |
| Washington | WA | 2927 | 2564 (2459, 2620) | 363 (257, 418) | 47.7 (33.7, 54.9) | 14.2 (10.1, 18.3) |
| West Virginia | WV | 221 | 237 (207, 254) | -16 (-46, 1) | -8.9 (-25.5, 0.6) | -6.8 (-18.6, 5.9) |
| Wisconsin | WI | 2326 | 1917 (1831, 1962) | 409 (323, 455) | 69.7 (55.0, 77.5) | 21.3 (16.5, 26.3) |

Abbreviation: CI, confidence interval

Note: Gastrointestinal hemorrhage was the contributing causes of death.

^a^ Excess death number estimated by subtract the expected number from the observed number of death.

^b^ Excess mortality per 1,000,000 persons was estimated via the excess death number divided by population size.

^c^ Excess risk was calculated as the ratio of the excess-to-expected number of death.

# Table S13. The estimated excess mortality of colorectal cancer by state from March 2020 to September 2022 (United States. 2020-2022)

| **State** | **Abbreviation** | **Observed deaths, No.** | **Expected deaths,**  **No. (95% CI)** | **Excess deaths,**  **No. (95% CI) ^a^** | **Excess mortality**  **(95% CI) ^b^** | **Excess risk % (95% CI) ^c^** |
| --- | --- | --- | --- | --- | --- | --- |
| Alabama | AL | 2745 | 2495 (2385, 2554) | 250 (139, 308) | 50.0 (27.8, 61.6) | 10.0 (5.9, 14.2) |
| Arkansas | AR | 1667 | 2008 (1920, 2055) | -341 (-429, -294) | -113.4 (-142.7, -97.8) | -17.0 (-20.9, -12.9) |
| Arizona | AZ | 3446 | 3134 (3009, 3199) | 312 (188, 378) | 44.1 (26.6, 53.4) | 10.0 (6.3, 13.7) |
| California | CA | 16790 | 16497 (16235, 16633) | 293 (31, 429) | 7.4 (0.8, 10.9) | 1.8 (0.2, 3.3) |
| Colorado | CO | 2395 | 2107 (2017, 2154) | 288 (199, 336) | 50.3 (34.8, 58.7) | 13.7 (9.2, 18.3) |
| Connecticut | CT | 1159 | 1158 (1092, 1194) | 1 (-66, 37) | 0.3 (-18.3, 10.3) | 0.1 (-5.6, 5.9) |
| Florida | FL | 11901 | 10828 (10605, 10944) | 1073 (849, 1189) | 50.3 (39.8, 55.7) | 9.9 (7.9, 11.9) |
| Georgia | GA | 5014 | 4624 (4490, 4694) | 390 (257, 460) | 36.7 (24.2, 43.3) | 8.4 (5.5, 11.5) |
| Iowa | IA | 1645 | 1515 (1439, 1556) | 130 (54, 171) | 40.9 (17.0, 53.8) | 8.6 (3.4, 13.9) |
| Idaho | ID | 218 | 194 (166, 209) | 24 (-3, 40) | 13.2 (-1.7, 22.1) | 12.4 (-2.1, 27.8) |
| Illinois | IL | 6242 | 6097 (5926, 6187) | 145 (-27, 234) | 11.3 (-2.1, 18.3) | 2.4 (-0.1, 4.9) |
| Indiana | IN | 3763 | 4206 (4074, 4275) | -443 (-574, -374) | -65.6 (-85.0, -55.4) | -10.5 (-13.4, -7.7) |
| Kansas | KS | 1286 | 1128 (1062, 1163) | 158 (92, 194) | 53.9 (31.4, 66.2) | 14.0 (7.9, 20.3) |
| Kentucky | KY | 2934 | 2685 (2569, 2747) | 249 (132, 310) | 55.4 (29.4, 69.0) | 9.3 (5.4, 13.3) |
| Louisiana | LA | 2468 | 2435 (2338, 2486) | 33 (-63, 85) | 7.1 (-13.5, 18.3) | 1.4 (-2.6, 5.4) |
| Massachusetts | MA | 2756 | 2791 (2688, 2846) | -35 (-139, 19) | -5.0 (-19.9, 2.7) | -1.3 (-4.9, 2.5) |
| Maryland | MD | 3115 | 3174 (3063, 3232) | -59 (-169, 0) | -9.6 (-27.5, 0.0) | -1.9 (-5.3, 1.6) |
| Maine | ME | 104 | 162 (137, 177) | -58 (-83, -43) | -42.7 (-61.2, -31.7) | -35.8 (-47.5, -22.9) |
| Michigan | MI | 5364 | 5110 (4967, 5185) | 254 (111, 329) | 25.2 (11.0, 32.7) | 5.0 (2.2, 7.8) |
| Minnesota | MN | 2649 | 2675 (2573, 2728) | -26 (-127, 28) | -4.6 (-22.4, 4.9) | -1.0 (-4.7, 2.8) |
| Missouri | MO | 3369 | 2870 (2762, 2927) | 499 (392, 556) | 81.3 (63.8, 90.5) | 17.4 (13.5, 21.4) |
| Mississippi | MS | 1976 | 2039 (1951, 2086) | -63 (-152, -16) | -21.2 (-51.2, -5.4) | -3.1 (-7.3, 1.2) |
| North Carolina | NC | 4913 | 4991 (4853, 5064) | -78 (-217, -6) | -7.5 (-20.9, -0.6) | -1.6 (-4.3, 1.2) |
| Nebraska | NE | 527 | 585 (538, 611) | -58 (-106, -32) | -29.7 (-54.3, -16.4) | -9.9 (-17.4, -2.1) |
| New Jersey | NJ | 4315 | 4107 (3982, 4173) | 208 (82, 274) | 22.5 (8.9, 29.7) | 5.1 (2.0, 8.2) |
| New Mexico | NM | 382 | 349 (312, 369) | 33 (-3, 54) | 15.6 (-1.4, 25.6) | 9.5 (-1.2, 20.7) |
| Nevada | NV | 1178 | 1199 (1131, 1235) | -21 (-89, 16) | -6.9 (-29.1, 5.2) | -1.8 (-7.3, 3.9) |
| New York | NY | 9002 | 8856 (8663, 8957) | 146 (-48, 246) | 7.3 (-2.4, 12.2) | 1.6 (-0.4, 3.8) |
| Ohio | OH | 6471 | 5901 (5751, 5980) | 570 (419, 649) | 48.4 (35.6, 55.1) | 9.7 (7.0, 12.3) |
| Oklahoma | OK | 2467 | 2122 (2028, 2172) | 345 (250, 395) | 87.4 (63.3, 100.0) | 16.3 (11.7, 20.9) |
| Oregon | OR | 2120 | 2040 (1951, 2087) | 80 (-9, 127) | 19.0 (-2.1, 30.2) | 3.9 (-0.5, 8.4) |
| Pennsylvania | PA | 7290 | 6763 (6594, 6851) | 527 (358, 615) | 40.6 (27.6, 47.4) | 7.8 (5.3, 10.3) |
| South Carolina | SC | 2870 | 2974 (2867, 3030) | -104 (-210, -47) | -20.5 (-41.3, -9.3) | -3.5 (-7.0, 0.1) |
| Tennessee | TN | 3901 | 3879 (3757, 3944) | 22 (-100, 86) | 3.2 (-14.6, 12.5) | 0.6 (-2.6, 3.7) |
| Texas | TX | 12798 | 12225 (12004, 12340) | 573 (351, 688) | 19.9 (12.2, 23.8) | 4.7 (2.9, 6.5) |
| Virginia | VA | 4060 | 4152 (4016, 4224) | -92 (-229, -21) | -10.7 (-26.7, -2.4) | -2.2 (-5.2, 0.8) |
| Washington | WA | 3424 | 3097 (2988, 3154) | 327 (218, 385) | 42.9 (28.6, 50.5) | 10.6 (6.9, 14.3) |
| West Virginia | WV | 877 | 1053 (989, 1087) | -176 (-240, -142) | -97.7 (-133.3, -78.8) | -16.7 (-22.1, -11.1) |
| Wisconsin | WI | 2754 | 2384 (2289, 2435) | 370 (274, 420) | 63.0 (46.7, 71.5) | 15.5 (11.2, 19.9) |

Abbreviation: CI, confidence interval

Note: Colorectal cancer was the contributing causes of death.

^a^ Excess death number estimated by subtract the expected number from the observed number of death.

^b^ Excess mortality per 1,000,000 persons was estimated via the excess death number divided by population size.

^c^ Excess risk was calculated as the ratio of the excess-to-expected number of death.

# Table S14. The estimated excess mortality of alcoholic liver disease by state from March 2020 to September 2022 (United States. 2020-2022)

| **State** | **Abbreviation** | **Observed deaths, No.** | **Expected deaths,**  **No. (95% CI)** | **Excess deaths,**  **No. (95% CI) ^a^** | **Excess mortality**  **(95% CI) ^b^** | **Excess risk % (95% CI) ^c^** |
| --- | --- | --- | --- | --- | --- | --- |
| Alabama | AL | 696 | 638 (589, 665) | 58 (8, 85) | 11.6 (1.6, 17.0) | 9.1 (1.1, 17.3) |
| Arizona | AZ | 3093 | 2258 (2165, 2308) | 835 (741, 884) | 118.0 (104.7, 124.9) | 37.0 (32.2, 41.8) |
| California | CA | 16263 | 13315 (13077, 13438) | 2948 (2710, 3072) | 74.7 (68.7, 77.9) | 22.1 (20.3, 24.0) |
| Colorado | CO | 3215 | 2717 (2615, 2771) | 498 (396, 552) | 87.0 (69.2, 96.4) | 18.3 (14.3, 22.5) |
| Florida | FL | 7001 | 5471 (5326, 5547) | 1530 (1385, 1606) | 71.7 (64.9, 75.3) | 28.0 (25.0, 31.0) |
| Georgia | GA | 2212 | 2122 (2032, 2170) | 90 (0, 138) | 8.5 (0.0, 13.0) | 4.2 (-0.1, 8.6) |
| Iowa | IA | 396 | 469 (427, 493) | -73 (-115, -49) | -23.0 (-36.2, -15.4) | -15.6 (-23.7, -7.0) |
| Illinois | IL | 2784 | 2474 (2377, 2526) | 310 (212, 361) | 24.2 (16.5, 28.2) | 12.5 (8.4, 16.7) |
| Indiana | IN | 2105 | 1993 (1905, 2039) | 112 (25, 159) | 16.6 (3.7, 23.6) | 5.6 (1.2, 10.2) |
| Kentucky | KY | 1043 | 1014 (952, 1048) | 29 (-34, 63) | 6.5 (-7.6, 14.0) | 2.9 (-3.3, 9.2) |
| Louisiana | LA | 433 | 308 (273, 327) | 125 (91, 145) | 26.8 (19.5, 31.1) | 40.6 (27.7, 54.1) |
| Massachusetts | MA | 1478 | 1370 (1297, 1409) | 108 (36, 147) | 15.4 (5.1, 21.0) | 7.9 (2.5, 13.5) |
| Maryland | MD | 924 | 801 (745, 831) | 123 (68, 154) | 20.0 (11.1, 25.0) | 15.4 (8.0, 22.9) |
| Michigan | MI | 3199 | 2432 (2335, 2483) | 767 (670, 818) | 76.2 (66.6, 81.3) | 31.5 (27.0, 36.1) |
| Minnesota | MN | 2463 | 1871 (1786, 1916) | 592 (508, 638) | 104.4 (89.6, 112.5) | 31.6 (26.5, 36.9) |
| Missouri | MO | 1068 | 957 (896, 990) | 111 (51, 144) | 18.1 (8.3, 23.4) | 11.6 (5.0, 18.4) |
| North Carolina | NC | 2918 | 2145 (2055, 2194) | 773 (682, 821) | 74.6 (65.8, 79.2) | 36.0 (31.1, 41.0) |
| New Jersey | NJ | 1101 | 1052 (989, 1087) | 49 (-15, 83) | 5.3 (-1.6, 9.0) | 4.7 (-1.4, 10.9) |
| New Mexico | NM | 1566 | 1143 (1077, 1179) | 423 (357, 459) | 200.5 (169.2, 217.6) | 37.0 (30.3, 43.9) |
| Nevada | NV | 856 | 662 (612, 690) | 194 (143, 221) | 63.4 (46.7, 72.2) | 29.3 (20.8, 38.1) |
| New York | NY | 3562 | 2755 (2651, 2809) | 807 (704, 862) | 40.1 (35.0, 42.9) | 29.3 (25.1, 33.6) |
| Ohio | OH | 3398 | 2897 (2792, 2953) | 501 (395, 556) | 42.6 (33.6, 47.2) | 17.3 (13.4, 21.3) |
| Oklahoma | OK | 1827 | 1700 (1619, 1743) | 127 (46, 170) | 32.2 (11.7, 43.1) | 7.5 (2.6, 12.5) |
| Oregon | OR | 2411 | 1940 (1854, 1986) | 471 (385, 517) | 112.0 (91.5, 122.9) | 24.3 (19.4, 29.3) |
| Pennsylvania | PA | 2745 | 2454 (2357, 2506) | 291 (194, 342) | 22.4 (15.0, 26.4) | 11.9 (7.7, 16.1) |
| South Carolina | SC | 1801 | 1651 (1571, 1693) | 150 (71, 193) | 29.5 (14.0, 38.0) | 9.1 (4.1, 14.2) |
| Tennessee | TN | 2691 | 2390 (2294, 2441) | 301 (205, 352) | 43.9 (29.9, 51.3) | 12.6 (8.4, 16.9) |
| Texas | TX | 7876 | 6972 (6797, 7063) | 904 (729, 995) | 31.3 (25.3, 34.5) | 13.0 (10.5, 15.5) |
| Virginia | VA | 1624 | 1449 (1374, 1489) | 175 (101, 215) | 20.4 (11.8, 25.1) | 12.1 (6.7, 17.6) |
| Washington | WA | 3884 | 3444 (3327, 3505) | 440 (324, 502) | 57.8 (42.5, 65.9) | 12.8 (9.3, 16.4) |
| Wisconsin | WI | 2111 | 1892 (1807, 1938) | 219 (134, 264) | 37.3 (22.8, 45.0) | 11.6 (6.9, 16.4) |

Abbreviation: CI, confidence interval

Note: Alcoholic liver disease was the contributing causes of death.

^a^ Excess death number estimated by subtract the expected number from the observed number of death.

^b^ Excess mortality per 1,000,000 persons was estimated via the excess death number divided by population size.

^c^ Excess risk was calculated as the ratio of the excess-to-expected number of death.

# Table S15. The estimated excess mortality of hepatic fibrosis/cirrhosis by state from March 2020 to September 2022 (United States. 2020-2022)

| **State** | **Abbreviation** | **Observed deaths, No.** | **Expected deaths,**  **No. (95% CI)** | **Excess deaths,**  **No. (95% CI) ^a^** | **Excess mortality**  **(95% CI) ^b^** | **Excess risk % (95% CI) ^c^** |
| --- | --- | --- | --- | --- | --- | --- |
| Alabama | AL | 2639 | 2430 (2334, 2482) | 209 (112, 260) | 41.8 (22.4, 52.0) | 8.6 (4.5, 12.8) |
| Arkansas | AR | 1405 | 1436 (1362, 1476) | -31 (-105, 9) | -10.3 (-34.9, 3.0) | -2.2 (-7.2, 3.0) |
| Arizona | AZ | 3559 | 2815 (2705, 2873) | 744 (633, 802) | 105.1 (89.4, 113.3) | 26.4 (22.3, 30.6) |
| California | CA | 13299 | 12278 (12053, 12395) | 1021 (795, 1138) | 25.9 (20.1, 28.8) | 8.3 (6.5, 10.2) |
| Colorado | CO | 2193 | 2087 (1997, 2134) | 106 (17, 154) | 18.5 (3.0, 26.9) | 5.1 (0.7, 9.5) |
| Connecticut | CT | 715 | 767 (712, 796) | -52 (-106, -22) | -14.4 (-29.4, -6.1) | -6.8 (-13.5, 0.2) |
| Florida | FL | 9559 | 8356 (8177, 8449) | 1203 (1024, 1296) | 56.4 (48.0, 60.7) | 14.4 (12.1, 16.7) |
| Georgia | GA | 3758 | 2876 (2770, 2931) | 882 (777, 938) | 83.0 (73.1, 88.3) | 30.7 (26.5, 34.9) |
| Iowa | IA | 522 | 717 (664, 746) | -195 (-247, -166) | -61.3 (-77.7, -52.2) | -27.2 (-33.3, -20.8) |
| Illinois | IL | 4321 | 4106 (3969, 4178) | 215 (78, 287) | 16.8 (6.1, 22.4) | 5.2 (2.1, 8.4) |
| Indiana | IN | 3271 | 2983 (2876, 3040) | 288 (181, 344) | 42.7 (26.8, 51.0) | 9.7 (5.9, 13.4) |
| Kansas | KS | 447 | 570 (523, 595) | -123 (-169, -97) | -41.9 (-57.6, -33.1) | -21.6 (-28.7, -14.1) |
| Kentucky | KY | 3070 | 2874 (2766, 2932) | 196 (87, 253) | 43.6 (19.4, 56.3) | 6.8 (3.1, 10.6) |
| Louisiana | LA | 1544 | 1591 (1513, 1633) | -47 (-125, -5) | -10.1 (-26.8, -1.1) | -3.0 (-7.7, 1.9) |
| Massachusetts | MA | 2488 | 2169 (2077, 2217) | 319 (228, 368) | 45.6 (32.6, 52.6) | 14.7 (10.2, 19.3) |
| Maryland | MD | 2169 | 2127 (2036, 2175) | 42 (-48, 90) | 6.8 (-7.8, 14.6) | 2.0 (-2.3, 6.3) |
| Michigan | MI | 4123 | 3636 (3518, 3699) | 487 (368, 549) | 48.4 (36.6, 54.6) | 13.4 (10.0, 16.9) |
| Minnesota | MN | 1938 | 1850 (1766, 1895) | 88 (3, 133) | 15.5 (0.5, 23.5) | 4.8 (0.1, 9.5) |
| Missouri | MO | 2511 | 2006 (1918, 2052) | 505 (418, 552) | 82.2 (68.1, 89.9) | 25.2 (20.3, 30.1) |
| Mississippi | MS | 785 | 759 (705, 789) | 26 (-28, 55) | 8.8 (-9.4, 18.5) | 3.4 (-3.7, 10.8) |
| North Carolina | NC | 4853 | 5077 (4933, 5152) | -224 (-367, -148) | -21.6 (-35.4, -14.3) | -4.4 (-7.1, -1.7) |
| New Jersey | NJ | 2814 | 2465 (2365, 2518) | 349 (249, 402) | 37.8 (27.0, 43.5) | 14.2 (10.0, 18.4) |
| New Mexico | NM | 1380 | 1248 (1179, 1285) | 132 (63, 169) | 62.6 (29.9, 80.1) | 10.6 (4.8, 16.5) |
| Nevada | NV | 593 | 436 (395, 459) | 157 (116, 180) | 51.3 (37.9, 58.8) | 36.0 (25.3, 47.2) |
| New York | NY | 5012 | 4859 (4723, 4931) | 153 (16, 225) | 7.6 (0.8, 11.2) | 3.1 (0.3, 6.0) |
| Ohio | OH | 5331 | 5203 (5050, 5284) | 128 (-26, 208) | 10.9 (-2.2, 17.7) | 2.5 (-0.3, 5.2) |
| Oklahoma | OK | 2472 | 2229 (2136, 2278) | 243 (151, 292) | 61.5 (38.2, 74.0) | 10.9 (6.6, 15.3) |
| Oregon | OR | 1677 | 1296 (1226, 1334) | 381 (310, 419) | 90.6 (73.7, 99.6) | 29.4 (23.3, 35.7) |
| Pennsylvania | PA | 5556 | 5933 (5774, 6016) | -377 (-535, -294) | -29.1 (-41.2, -22.7) | -6.4 (-8.8, -3.9) |
| South Carolina | SC | 2636 | 2518 (2419, 2570) | 118 (19, 171) | 23.2 (3.7, 33.7) | 4.7 (0.7, 8.7) |
| Tennessee | TN | 3816 | 3836 (3701, 3907) | -20 (-155, 51) | -2.9 (-22.6, 7.4) | -0.5 (-3.7, 2.7) |
| Texas | TX | 16549 | 13789 (13531, 13922) | 2760 (2502, 2894) | 95.6 (86.7, 100.3) | 20.0 (18.2, 21.9) |
| Virginia | VA | 3537 | 3283 (3170, 3342) | 254 (142, 313) | 29.6 (16.5, 36.5) | 7.7 (4.2, 11.3) |
| Washington | WA | 2727 | 2570 (2471, 2623) | 157 (58, 210) | 20.6 (7.6, 27.6) | 6.1 (2.2, 10.1) |
| West Virginia | WV | 888 | 909 (850, 941) | -21 (-80, 11) | -11.7 (-44.4, 6.1) | -2.3 (-8.6, 4.2) |
| Wisconsin | WI | 1864 | 1843 (1759, 1888) | 21 (-63, 66) | 3.6 (-10.7, 11.2) | 1.1 (-3.4, 5.8) |

Abbreviation: CI, confidence interval

Note: Hepatic fibrosis/cirrhosis was the contributing causes of death.

^a^ Excess death number estimated by subtract the expected number from the observed number of death.

^b^ Excess mortality per 1,000,000 persons was estimated via the excess death number divided by population size.

^c^ Excess risk was calculated as the ratio of the excess-to-expected number of death.

# Table S16. The estimated excess mortality of hepatic failure by state from March 2020 to September 2022 (United States. 2020-2022)

| **State** | **Abbreviation** | **Observed deaths, No.** | **Expected deaths,**  **No. (95% CI)** | **Excess deaths,**  **No. (95% CI) ^a^** | **Excess mortality**  **(95% CI) ^b^** | **Excess risk % (95% CI) ^c^** |
| --- | --- | --- | --- | --- | --- | --- |
| Alabama | AL | 1211 | 1075 (1011, 1110) | 136 (72, 171) | 27.2 (14.4, 34.2) | 12.7 (6.4, 19.1) |
| Arkansas | AR | 267 | 155 (131, 170) | 112 (87, 126) | 37.3 (28.9, 41.9) | 72.3 (52.2, 93.5) |
| Arizona | AZ | 1716 | 1357 (1285, 1396) | 359 (287, 398) | 50.7 (40.5, 56.2) | 26.5 (20.5, 32.5) |
| California | CA | 8347 | 7648 (7474, 7738) | 699 (525, 789) | 17.7 (13.3, 20.0) | 9.1 (6.8, 11.5) |
| Colorado | CO | 1042 | 881 (822, 912) | 161 (103, 193) | 28.1 (18.0, 33.7) | 18.3 (11.2, 25.6) |
| Connecticut | CT | 119 | 137 (114, 151) | -18 (-41, -4) | -5.0 (-11.4, -1.1) | -13.1 (-28.0, 3.2) |
| Florida | FL | 4560 | 4293 (4158, 4363) | 267 (133, 338) | 12.5 (6.2, 15.8) | 6.2 (3.2, 9.3) |
| Georgia | GA | 2487 | 2721 (2619, 2775) | -234 (-337, -180) | -22.0 (-31.7, -16.9) | -8.6 (-12.2, -5.0) |
| Illinois | IL | 2312 | 2045 (1956, 2092) | 267 (179, 315) | 20.8 (14.0, 24.6) | 13.1 (8.5, 17.7) |
| Indiana | IN | 1577 | 1674 (1594, 1717) | -97 (-177, -54) | -14.4 (-26.2, -8.0) | -5.8 (-10.4, -1.1) |
| Kansas | KS | 214 | 230 (200, 247) | -16 (-46, 1) | -5.5 (-15.7, 0.3) | -7.0 (-19.0, 5.9) |
| Kentucky | KY | 994 | 871 (813, 902) | 123 (66, 155) | 27.4 (14.7, 34.5) | 14.1 (7.1, 21.3) |
| Louisiana | LA | 460 | 446 (405, 469) | 14 (-28, 37) | 3.0 (-6.0, 7.9) | 3.1 (-6.1, 12.8) |
| Massachusetts | MA | 1432 | 1353 (1281, 1392) | 79 (7, 118) | 11.3 (1.0, 16.9) | 5.8 (0.4, 11.4) |
| Maryland | MD | 1025 | 1009 (946, 1042) | 16 (-46, 50) | 2.6 (-7.5, 8.1) | 1.6 (-4.5, 7.9) |
| Michigan | MI | 2306 | 2604 (2504, 2657) | -298 (-398, -245) | -29.6 (-39.6, -24.3) | -11.4 (-15.0, -7.8) |
| Minnesota | MN | 791 | 948 (887, 980) | -157 (-217, -124) | -27.7 (-38.3, -21.9) | -16.6 (-22.3, -10.6) |
| Missouri | MO | 1238 | 1123 (1058, 1159) | 115 (49, 150) | 18.7 (8.0, 24.4) | 10.2 (4.2, 16.5) |
| North Carolina | NC | 2718 | 2289 (2195, 2339) | 429 (335, 479) | 41.4 (32.3, 46.2) | 18.7 (14.3, 23.2) |
| New Jersey | NJ | 1865 | 1759 (1677, 1803) | 106 (24, 150) | 11.5 (2.6, 16.2) | 6.0 (1.3, 10.9) |
| New York | NY | 3594 | 3859 (3729, 3927) | -265 (-395, -197) | -13.2 (-19.6, -9.8) | -6.9 (-9.9, -3.8) |
| Ohio | OH | 2659 | 2435 (2339, 2487) | 224 (127, 275) | 19.0 (10.8, 23.4) | 9.2 (5.1, 13.4) |
| Oklahoma | OK | 130 | 90 (71, 101) | 40 (22, 52) | 10.1 (5.6, 13.2) | 44.4 (20.7, 70.3) |
| Oregon | OR | 841 | 902 (843, 934) | -61 (-119, -29) | -14.5 (-28.3, -6.9) | -6.8 (-13.0, -0.4) |
| Pennsylvania | PA | 3245 | 2999 (2891, 3055) | 246 (139, 303) | 19.0 (10.7, 23.4) | 8.2 (4.5, 12.0) |
| South Carolina | SC | 1766 | 1844 (1760, 1889) | -78 (-162, -33) | -15.4 (-31.9, -6.5) | -4.2 (-8.6, 0.3) |
| Tennessee | TN | 2014 | 1964 (1878, 2011) | 50 (-37, 96) | 7.3 (-5.4, 14.0) | 2.5 (-1.9, 7.1) |
| Texas | TX | 6991 | 6399 (6242, 6481) | 592 (435, 674) | 20.5 (15.1, 23.4) | 9.3 (6.7, 11.8) |
| Utah | UT | 67 | 80 (63, 91) | -13 (-31, -2) | -4.0 (-9.6, -0.6) | -16.2 (-35.1, 5.0) |
| Virginia | VA | 1721 | 1427 (1353, 1467) | 294 (220, 334) | 34.3 (25.6, 38.9) | 20.6 (15.0, 26.4) |
| Washington | WA | 1953 | 1998 (1910, 2044) | -45 (-132, 2) | -5.9 (-17.3, 0.3) | -2.3 (-6.5, 2.1) |
| Wisconsin | WI | 1014 | 965 (904, 998) | 49 (-12, 82) | 8.3 (-2.0, 14.0) | 5.1 (-1.3, 11.6) |

Abbreviation: CI, confidence interval

Note: Hepatic failure was the contributing causes of death.

^a^ Excess death number estimated by subtract the expected number from the observed number of death.

^b^ Excess mortality per 1,000,000 persons was estimated via the excess death number divided by population size.

^c^ Excess risk was calculated as the ratio of the excess-to-expected number of death.

# Table S17. Excess mortality associated with digestive system diseases stratified by demographic factors and wave (United States. 2020-2022)

| **Group**^a^ | **Observed deaths, No.** | **Expected deaths,**  **No. (95% CI)** | **Excess deaths,**  **No. (95% CI) ^b^** | **Excess mortality**  **(95% CI) ^c^** | **Excess risk % (95% CI) ^d^** |
| --- | --- | --- | --- | --- | --- |
| Overall | 704701 | 624873 (622596, 626036) | 79828 (77551, 80992) | 242.1 (235.2, 245.6) | 12.8 (12.5, 13.0) |
| Age |  |  |  |  |  |
| 20-64 years | 254018 | 214323 (213276, 214859) | 39695 (38648, 40231) | 204.8 (199.4, 207.5) | 18.5 (18.1, 19.0) |
| 65-84 years | 322277 | 288553 (287190, 289251) | 33724 (32361, 34421) | 723.9 (694.6, 738.8) | 11.7 (11.3, 12.1) |
| Sex |  |  |  |  |  |
| Female | 323920 | 288931 (287574, 289625) | 34989 (33633, 35684) | 210.1 (202.0, 214.3) | 12.1 (11.7, 12.5) |
| Male | 380781 | 336123 (334768, 336817) | 44658 (43302, 45351) | 273.6 (265.3, 277.9) | 13.3 (12.9, 13.6) |
| Race/ethnicity ^e^ |  |  |  |  |  |
| NHW | 516533 | 464134 (462268, 465088) | 52399 (50533, 53353) | 267.3 (257.8, 272.2) | 11.3 (11.0, 11.6) |
| NHB | 74891 | 64032 (63478, 64317) | 10859 (10305, 11144) | 270.1 (256.4, 277.2) | 17.0 (16.1, 17.8) |
| Hispanic | 76152 | 63166 (62674, 63419) | 12986 (12493, 13239) | 213.6 (205.5, 217.7) | 20.6 (19.7, 21.4) |
| Wave ^f^ |  |  |  |  |  |
| Wave I | 66293 | 63707 (62980, 64080) | 2586 (1859, 2959) | 7.8 (5.6, 9.0) | 4.1 (3.3, 4.9) |
| Wave II | 83980 | 74388 (73603, 74791) | 9592 (8806, 9994) | 29.1 (26.7, 30.3) | 12.9 (12.1, 13.7) |
| Wave III | 204547 | 179373 (178153, 179998) | 25174 (23954, 25798) | 76.3 (72.6, 78.2) | 14.0 (13.5, 14.5) |
| Wave IV | 119227 | 100953 (100037, 101421) | 18274 (17359, 18743) | 55.4 (52.6, 56.8) | 18.1 (17.4, 18.8) |
| Wave V | 144446 | 127946 (126916, 128474) | 16500 (15470, 17028) | 50.0 (46.9, 51.6) | 12.9 (12.3, 13.5) |
| Wave VI | 86208 | 78505 (77698, 78919) | 7703 (6896, 8117) | 23.4 (20.9, 24.6) | 9.8 (9.1, 10.5) |

Abbreviations: CI, confidence interval; NHW, non-Hispanic White; NHB, non-Hispanic Black.

^a^ The contributing cause of death was adopted. Digestive system diseases included diseases of oral cavity, salivary glands and jaws (K00-K14), diseases of esophagus, stomach and duodenum (K20-K31), diseases of appendix (K35-K38), hernia (K40-K46), noninfective enteritis and colitis (K50-K52), other diseases of intestines (K65-K67), diseases of liver (K70-K77), diseases of gallbladder, biliary tract and pancreas (K80-K87), other diseases of digestive system (K90-K93).

^b^ Excess death number estimated by subtract the expected number from the observed number of death.

^c^ Excess mortality per 1,000,000 persons was estimated via the excess death number divided by population size.

^d^ Excess risk was calculated as the ratio of the excess-to-expected number of death.

^e^ Non-Hispanic unknown and non-Hispanic AIAN was excluded when stratified by race/ethnicity.

^f^ Waves were identified according to the weekly surveillance of COVID-19 deaths in the US. Wave I was from March 2020 to June 2020, Wave II was from June 2020 to October 2020, Wave III was from October 2020 to June 2021, Wave IV was from June 2021 to November 2021, Wave V was from November 2021 to May 2022 and Wave VI was from May 2022 to September 2022.

# Table S18. Excess mortality associated with digestive organ malignancies stratified by demographic factors and wave (United States. 2020-2022)

| **Group**^a^ | **Observed deaths, No.** | **Expected deaths,**  **No. (95% CI)** | **Excess deaths,**  **No. (95% CI) ^b^** | **Excess mortality**  **(95% CI) ^c^** | **Excess risk % (95% CI) ^d^** |
| --- | --- | --- | --- | --- | --- |
| Overall | 469511 | 468339 (466332, 469364) | 1172 (-834, 2198) | 3.6 (-2.5, 6.7) | 0.3 (0.0, 0.5) |
| Age |  |  |  |  |  |
| 20-64 years | 131534 | 132056 (131165, 132512) | -522 (-1413, -65) | -2.7 (-7.3, -0.3) | -0.4 (-0.9, 0.1) |
| 65-84 years | 262519 | 254279 (253025, 254920) | 8240 (6986, 8882) | 176.9 (150.0, 190.6) | 3.2 (2.8, 3.6) |
| Sex |  |  |  |  |  |
| Female | 196697 | 194703 (193565, 195285) | 1994 (856, 2577) | 12.0 (5.1, 15.5) | 1.0 (0.6, 1.5) |
| Male | 272814 | 273918 (272658, 274563) | -1104 (-2364, -459) | -6.8 (-14.5, -2.8) | -0.4 (-0.8, 0.0) |
| Race/ethnicity ^e^ |  |  |  |  |  |
| NHW | 339635 | 338311 (336768, 339100) | 1324 (-219, 2113) | 6.8 (-1.1, 10.8) | 0.4 (0.1, 0.7) |
| NHB | 58673 | 58704 (58170, 58979) | -31 (-565, 243) | -0.8 (-14.1, 6.0) | -0.1 (-0.9, 0.8) |
| Hispanic | 44187 | 42525 (42110, 42739) | 1662 (1246, 1876) | 27.3 (20.5, 30.9) | 3.9 (2.9, 4.9) |
| Wave ^f^ |  |  |  |  |  |
| Wave I | 47762 | 47836 (47195, 48166) | -74 (-716, 255) | -0.2 (-2.2, 0.8) | -0.2 (-1.0, 0.7) |
| Wave II | 58121 | 58162 (57455, 58525) | -41 (-749, 321) | -0.1 (-2.3, 1.0) | -0.1 (-0.9, 0.7) |
| Wave III | 133504 | 132783 (131715, 133330) | 721 (-348, 1268) | 2.2 (-1.1, 3.8) | 0.5 (0.0, 1.1) |
| Wave IV | 78025 | 77469 (76653, 77887) | 556 (-260, 975) | 1.7 (-0.8, 3.0) | 0.7 (0.0, 1.4) |
| Wave V | 92269 | 92259 (91368, 92715) | 10 (-880, 467) | 0.0 (-2.7, 1.4) | 0.0 (-0.6, 0.7) |
| Wave VI | 59830 | 59829 (59112, 60197) | 1 (-717, 369) | 0.0 (-2.2, 1.1) | 0.0 (-0.8, 0.8) |

Abbreviations: CI, confidence interval; NHW, non-Hispanic White; NHB, non-Hispanic Black.

^a^ The contributing cause of death was adopted. Digestive organ malignancies included malignant neoplasm of esophagus (C15), stomach (C16), small intestine (C17), colon (C18), rectosigmoid junction (C19), rectum (C20), anus and anal canal (C21), liver and intrahepatic bile duct (C22), gallbladder (C23), other and unspecified parts of biliary tract (C24), pancreas (C25), and other and ill-defined digestive organs (C26).

^b^ Excess death number estimated by subtract the expected number from the observed number of death.

^c^ Excess mortality per 1,000,000 persons was estimated via the excess death number divided by population size.

^d^ Excess risk was calculated as the ratio of the excess-to-expected number of death.

^e^ Non-Hispanic unknown and non-Hispanic AIAN was excluded when stratified by race/ethnicity.

^f^ Waves were identified according to the weekly surveillance of COVID-19 deaths in the US. Wave I was from March 2020 to June 2020, Wave II was from June 2020 to October 2020, Wave III was from October 2020 to June 2021, Wave IV was from June 2021 to November 2021, Wave V was from November 2021 to May 2022 and Wave VI was from May 2022 to September 2022.

# Table S19. Excess mortality associated with gastrointestinal hemorrhage stratified by demographic factors and wave (United States. 2020-2022)

| **Group** ^a^ | **Observed deaths, No.** | **Expected deaths,**  **No. (95% CI)** | **Excess deaths,**  **No. (95% CI) ^b^** | **Excess mortality**  **(95% CI) ^c^** | **Excess risk % (95% CI) ^d^** |
| --- | --- | --- | --- | --- | --- |
| Overall | 136491 | 110004 (109239, 110397) | 26487 (25721, 26879) | 80.3 (78.0, 81.5) | 24.1 (23.4, 24.7) |
| Age |  |  |  |  |  |
| 20-64 years | 37951 | 27602 (27153, 27834) | 10349 (9899, 10580) | 53.4 (51.1, 54.6) | 37.5 (36.1, 38.9) |
| 65-84 years | 59531 | 47681 (47206, 47925) | 11850 (11375, 12094) | 254.4 (244.2, 259.6) | 24.9 (23.9, 25.9) |
| Sex |  |  |  |  |  |
| Female | 58755 | 47363 (46863, 47620) | 11392 (10892, 11649) | 68.4 (65.4, 70.0) | 24.1 (23.1, 25.1) |
| Male | 77736 | 62699 (62114, 63000) | 15037 (14452, 15337) | 92.1 (88.6, 94.0) | 24.0 (23.1, 24.9) |
| Race/ethnicity ^e^ |  |  |  |  |  |
| NHW | 98692 | 81694 (81036, 82031) | 16998 (16340, 17336) | 86.7 (83.4, 88.4) | 20.8 (20.1, 21.6) |
| NHB | 15390 | 11863 (11626, 11987) | 3527 (3289, 3650) | 87.7 (81.8, 90.8) | 29.7 (27.7, 31.8) |
| Hispanic | 13853 | 9537 (9324, 9647) | 4316 (4103, 4427) | 71.0 (67.5, 72.8) | 45.3 (42.8, 47.7) |
| Wave ^f^ |  |  |  |  |  |
| Wave I | 12863 | 11583 (11334, 11711) | 1280 (1032, 1409) | 3.9 (3.1, 4.3) | 11.1 (9.1, 13.0) |
| Wave II | 16248 | 13124 (12860, 13261) | 3124 (2859, 3261) | 9.5 (8.7, 9.9) | 23.8 (21.9, 25.7) |
| Wave III | 40665 | 32179 (31765, 32392) | 8486 (8072, 8699) | 25.7 (24.5, 26.4) | 26.4 (25.1, 27.6) |
| Wave IV | 22822 | 17431 (17127, 17589) | 5391 (5086, 5548) | 16.3 (15.4, 16.8) | 30.9 (29.2, 32.6) |
| Wave V | 28062 | 22469 (22123, 22647) | 5593 (5247, 5772) | 17.0 (15.9, 17.5) | 24.9 (23.4, 26.4) |
| Wave VI | 15831 | 13218 (12953, 13356) | 2613 (2347, 2750) | 7.9 (7.1, 8.3) | 19.8 (17.9, 21.6) |

Abbreviations: CI, confidence interval; NHW, non-Hispanic White; NHB, non-Hispanic Black.

^a^ The contributing cause of death was adopted. Gastrointestinal hemorrhage included esophageal varices with bleeding (I85.0); gastro-esophageal laceration-hemorrhage syndrome (K22.6); hemorrhage of esophagus, NOS (K22.8); gastric ulcer with acute bleeding (K25.0), acute bleeding and perforation (K25.2), chronic or unspecified bleeding (K25.4), chronic or unspecified bleeding and perforation (K25.6); and duodenal ulcers (K26.0, K26.2, K26.4, and K26.6), gastric ulcer at unspecified site (K27.0, K27.2, K27.4, and K27.6), and gastrojejunostomy ulcer (K28.0, K28.2, K28.4, and K28.6) as classified above; acute (corrosive) gastritis with bleeding (K29.0); anus and rectum bleeding (K62.5) and GI bleeding, NOS (K92.2).

^b^ Excess death number estimated by subtract the expected number from the observed number of death.

^c^ Excess mortality per 1,000,000 persons was estimated via the excess death number divided by population size.

^d^ Excess risk was calculated as the ratio of the excess-to-expected number of death.

^e^ Non-Hispanic unknown and non-Hispanic AIAN was excluded when stratified by race/ethnicity.

^f^ Waves were identified according to the weekly surveillance of COVID-19 deaths in the US. Wave I was from March 2020 to June 2020, Wave II was from June 2020 to October 2020, Wave III was from October 2020 to June 2021, Wave IV was from June 2021 to November 2021, Wave V was from November 2021 to May 2022 and Wave VI was from May 2022 to September 2022.

# Table S20. Sensitivity analysis of excess mortality associated with digestive related diseases from March 2020 to September 2022.^a^ (United States. 2020-2022)

| **Cause of death ^b^** | **Observed deaths, No.** | **Expected deaths,**  **No. (95% CI)** | **Excess deaths,**  **No. (95% CI) ^c^** | **Excess mortality**  **(95% CI) ^d^** | **Excess risk % (95% CI) ^e^** |
| --- | --- | --- | --- | --- | --- |
| **GI diseases** | | | | | |
| GI hemorrhage, NOS | 111669 | 91855 (91202, 92190) | 19814 (19161, 20149) | 59.7 (57.7, 60.7) | 21.6 (20.9, 22.3) |
| Ulcers | 21310 | 18935 (18644, 19086) | 2375 (2083, 2525) | 7.2 (6.3, 7.6) | 12.5 (11.0, 14.1) |
| Paralytic and intestinal obstruction | 51971 | 50007 (49519, 50258) | 1964 (1476, 2215) | 5.9 (4.4, 6.7) | 3.9 (3.0, 4.8) |
| Intestine vascular disorder | 41516 | 42808 (42344, 43047) | -1292 (-1757, -1054) | -3.9 (-5.3, -3.2) | -3.0 (-3.9, -2.1) |
| *C. difficile* colitis | 20533 | 16050 (15802, 16179) | 4483 (4234, 4611) | 13.5 (12.8, 13.9) | 27.9 (26.2, 29.7) |
| EC | 45080 | 48828 (48395, 49051) | -3748 (-4181, -3525) | -11.3 (-12.6, -10.6) | -7.7 (-8.5, -6.8) |
| GC | 31249 | 31772 (31423, 31952) | -523 (-872, -343) | -1.6 (-2.6, -1.0) | -1.6 (-2.7, -0.6) |
| CRC | 162973 | 158284 (157461, 158705) | 4689 (3867, 5111) | 14.1 (11.7, 15.4) | 3.0 (2.5, 3.5) |
| **Liver and pancreatic diseases** | | | | | |
| ALD | 105612 | 88713 (88129, 89012) | 16899 (16316, 17199) | 50.9 (49.2, 51.8) | 19.0 (18.3, 19.8) |
| Fibrosis/cirrhosis | 136997 | 128566 (127849, 128933) | 8431 (7715, 8799) | 25.4 (23.2, 26.5) | 6.6 (6.0, 7.1) |
| Chronic hepatitis C | 35540 | 31445 (31097, 31624) | 4095 (3747, 4274) | 12.3 (11.3, 12.9) | 13.0 (11.9, 14.2) |
| Hepatic failure | 79265 | 74938 (74398, 75216) | 4327 (3787, 4604) | 13.0 (11.4, 13.9) | 5.8 (5.0, 6.5) |
| LIHC | 82556 | 81132 (80573, 81418) | 1424 (866, 1711) | 4.3 (2.6, 5.2) | 1.8 (1.1, 2.5) |
| AP | 18297 | 15188 (14935, 15319) | 3109 (2856, 3240) | 9.4 (8.6, 9.8) | 20.5 (18.7, 22.2) |
| PC | 129727 | 130159 (129416, 130540) | -432 (-1175, -51) | -1.3 (-3.5, -0.2) | -0.3 (-0.9, 0.2) |

Abbreviations: CI, confidence interval; GI, gastrointestinal; NOS, not otherwise specified; *C. difficile*, *Clostridium difficile*; EC, esophageal cancer; GC, gastric cancer; CRC, colorectal cancer; ALD, alcohol liver disease; LIHC, liver and intrahepatic cancer; AP, acute pancreatitis; PC, pancreatic cancer.

^a^ Sensitivity analysis was conducted by removing January 2018 and February 2018.

^b^ The contributing cause of death was adopted.

^c^ Excess death number estimated by subtract the expected number from the observed number of death.

^d^ Excess mortality per 1,000,000 persons was estimated via the excess death number divided by population size.

^e^ Excess risk was calculated as the ratio of the excess-to-expected number of death.

#

# Table S21. Sensitivity analysis of excess mortality associated with digestive related diseases from March 2020 to September 2022.^a^ (United States. 2020-2022)

| **Cause of death ^b^** | **Observed deaths, No.** | **Expected deaths,**  **No. (95% CI)** | **Excess deaths,**  **No. (95% CI) ^c^** | **Excess mortality**  **(95% CI) ^d^** | **Excess risk % (95% CI) ^e^** |
| --- | --- | --- | --- | --- | --- |
| **GI diseases** | | | | | |
| GI hemorrhage, NOS | 111669 | 90060 (89410, 90393) | 21609 (20959, 21943) | 65.1 (63.1, 66.1) | 24.0 (23.3, 24.7) |
| Ulcers | 21310 | 18327 (18058, 18466) | 2983 (2715, 3122) | 9.0 (8.2, 9.4) | 16.3 (14.7, 17.8) |
| Paralytic ileus and intestine obstruction | 51971 | 51155 (50673, 51403) | 816 (333, 1064) | 2.5 (1.0, 3.2) | 1.6 (0.7, 2.5) |
| Vascular disorder of intestine | 41516 | 42394 (41933, 42632) | -878 (-1340, -641) | -2.6 (-4.0, -1.9) | -2.1 (-3.0, -1.1) |
| *C. difficile* colitis | 20533 | 15148 (14906, 15273) | 5385 (5143, 5510) | 16.2 (15.5, 16.6) | 35.5 (33.7, 37.4) |
| EC | 45080 | 47515 (47069, 47745) | -2435 (-2881, -2205) | -7.3 (-8.7, -6.6) | -5.1 (-6.0, -4.2) |
| GC | 31249 | 31903 (31553, 32084) | -654 (-1004, -474) | -2.0 (-3.0, -1.4) | -2.0 (-3.1, -1.0) |
| CRC | 162973 | 158037 (157153, 158490) | 4936 (4051, 5389) | 14.9 (12.2, 16.2) | 3.1 (2.6, 3.6) |
| **Liver and pancreatic diseases** | | | | | |
| ALD | 105612 | 88367 (87779, 88669) | 17245 (16657, 17547) | 52.0 (50.2, 52.9) | 19.5 (18.8, 20.2) |
| Fibrosis/cirrhosis | 136997 | 126465 (125632, 126892) | 10532 (9700, 10959) | 31.7 (29.2, 33.0) | 8.3 (7.8, 8.9) |
| Chronic hepatitis C | 35540 | 30956 (30590, 31145) | 4584 (4217, 4773) | 13.8 (12.7, 14.4) | 14.8 (13.6, 16.0) |
| Hepatic failure | 79265 | 74100 (73505, 74405) | 5165 (4570, 5471) | 15.6 (13.8, 16.5) | 7.0 (6.2, 7.7) |
| LIHC | 82556 | 82244 (81599, 82575) | 312 (-333, 643) | 0.9 (-1.0, 1.9) | 0.4 (-0.3, 1.1) |
| AP | 18297 | 15201 (14941, 15335) | 3096 (2836, 3231) | 9.3 (8.5, 9.7) | 20.4 (18.6, 22.1) |
| PC | 129727 | 132602 (131774, 133027) | -2875 (-3704, -2451) | -8.7 (-11.2, -7.4) | -2.2 (-2.7, -1.6) |

Abbreviations: CI, confidence interval; GI, gastrointestinal; NOS, not otherwise specified; *C. difficile*, *Clostridium difficile*; EC, esophageal cancer; GC, gastric cancer; CRC, colorectal cancer; ALD, alcohol liver disease; LIHC, liver and intrahepatic cancer; AP, acute pancreatitis; PC, pancreatic cancer.

^a^ Sensitivity analysis was conducted with harmonics = 8.

^b^ The contributing cause of death was adopted.

^c^ Excess death number estimated by subtract the expected number from the observed number of death.

^d^ Excess mortality per 1,000,000 persons was estimated via the excess death number divided by population size.

^e^ Excess risk was calculated as the ratio of the excess-to-expected number of death.

#

# Table S22. Sensitivity analysis of excess mortality associated with selected gastrointestinal diseases stratified by demographic factor and pandemic wave. ^a^ (United States. 2020-2022)

| **Cause of death ^b^** | **Observed deaths, No.** | **Expected deaths,**  **No. (95% CI)** | **Excess deaths,**  **No. (95% CI) ^c^** | **Excess mortality**  **(95% CI) ^d^** | **Excess risk % (95% CI) ^e^** |
| --- | --- | --- | --- | --- | --- |
| **GI hemorrhage, NOS** | | | | | |
| Age |  |  |  |  |  |
| 20-64 years | 29214 | 21699 (21358, 21875) | 7515 (7173, 7691) | 38.8 (37.0, 39.7) | 34.6 (33.1, 36.2) |
| 65-84 years | 51555 | 41792 (41387, 42000) | 9763 (9358, 9972) | 209.6 (200.9, 214.0) | 23.4 (22.3, 24.4) |
| Sex |  |  |  |  |  |
| Female | 49701 | 40816 (40390, 41035) | 8885 (8459, 9104) | 53.4 (50.8, 54.7) | 21.8 (20.7, 22.8) |
| Male | 61968 | 50678 (50158, 50945) | 11290 (10770, 11557) | 69.2 (66.0, 70.8) | 22.3 (21.3, 23.2) |
| Race/ethnicity ^f^ |  |  |  |  |  |
| NHW | 81314 | 68556 (67963, 68860) | 12758 (12166, 13062) | 65.1 (62.1, 66.6) | 18.6 (17.8, 19.4) |
| NHB | 13875 | 11073 (10839, 11194) | 2802 (2568, 2924) | 69.7 (63.9, 72.7) | 25.3 (23.2, 27.4) |
| Hispanic | 10322 | 6891 (6728, 6976) | 3431 (3269, 3516) | 56.4 (53.8, 57.8) | 49.8 (46.9, 52.7) |
| Wave ^g^ |  |  |  |  |  |
| Wave I | 10532 | 9612 (9400, 9721) | 920 (709, 1030) | 2.8 (2.1, 3.1) | 9.6 (7.5, 11.7) |
| Wave II | 13288 | 10842 (10618, 10959) | 2446 (2221, 2562) | 7.4 (6.7, 7.7) | 22.6 (20.5, 24.7) |
| Wave III | 33477 | 26748 (26396, 26930) | 6729 (6376, 6911) | 20.3 (19.2, 20.8) | 25.2 (23.8, 26.5) |
| Wave IV | 18776 | 14597 (14337, 14732) | 4179 (3918, 4314) | 12.6 (11.8, 13.0) | 28.6 (26.8, 30.5) |
| Wave V | 22879 | 18884 (18587, 19037) | 3995 (3699, 4148) | 12.0 (11.1, 12.5) | 21.2 (19.6, 22.7) |
| Wave VI | 12717 | 11172 (10945, 11291) | 1545 (1317, 1663) | 4.7 (4.0, 5.0) | 13.8 (11.9, 15.8) |
| **Ulcers** | - | - | - | - | - |
| Age |  |  |  |  |  |
| 20-64 years | 3156 | 2444 (2285, 2527) | 712 (554, 796) | 3.7 (2.9, 4.1) | 29.1 (24.7, 33.7) |
| 65-84 years | 10705 | 10678 (10357, 10844) | 27 (-294, 192) | 0.6 (-6.3, 4.1) | 0.3 (-1.6, 2.2) |
| Sex |  |  |  |  |  |
| Female | 9976 | 8754 (8561, 8855) | 1222 (1028, 1322) | 7.3 (6.2, 7.9) | 14.0 (11.7, 16.2) |
| Male | 11334 | 10102 (9889, 10212) | 1232 (1019, 1343) | 7.5 (6.2, 8.2) | 12.2 (10.1, 14.3) |
| Race/ethnicity ^f^ |  |  |  |  |  |
| NHW | 15851 | 14522 (14286, 14644) | 1329 (1093, 1452) | 6.8 (5.6, 7.4) | 9.2 (7.5, 10.9) |
| NHB | 2216 | 1756 (1674, 1800) | 460 (378, 504) | 11.4 (9.4, 12.5) | 26.2 (21.0, 31.5) |
| Hispanic | 1676 | 1485 (1409, 1525) | 191 (116, 232) | 3.1 (1.9, 3.8) | 12.9 (7.5, 18.3) |
| Wave ^g^ |  |  |  |  |  |
| Wave I | 2059 | 1992 (1897, 2042) | 67 (-27, 117) | 0.2 (-0.1, 0.4) | 3.4 (-1.1, 7.9) |
| Wave II | 2478 | 2108 (2010, 2159) | 370 (273, 422) | 1.1 (0.8, 1.3) | 17.6 (13.0, 22.2) |
| Wave III | 6201 | 5569 (5412, 5652) | 632 (474, 714) | 1.9 (1.4, 2.2) | 11.3 (8.6, 14.1) |
| Wave IV | 3559 | 2952 (2837, 3013) | 607 (492, 668) | 1.8 (1.5, 2.0) | 20.6 (16.6, 24.6) |
| Wave V | 4481 | 4048 (3913, 4118) | 433 (299, 504) | 1.3 (0.9, 1.5) | 10.7 (7.5, 14.0) |
| Wave VI | 2532 | 2267 (2166, 2320) | 265 (165, 319) | 0.8 (0.5, 1.0) | 11.7 (7.4, 16.1) |
| ***C. difficile* colitis** | - | - | - | - | - |
| Age |  |  |  |  |  |
| 20-64 years | 1250 | 861 (793, 898) | 389 (321, 425) | 2.0 (1.7, 2.2) | 45.2 (37.2, 53.3) |
| 65-84 years | 11177 | 8341 (8141, 8444) | 2836 (2637, 2940) | 60.9 (56.6, 63.1) | 34.0 (31.5, 36.5) |
| Sex |  |  |  |  |  |
| Female | 11111 | 8505 (8324, 8599) | 2606 (2426, 2701) | 15.6 (14.6, 16.2) | 30.6 (28.2, 33.1) |
| Male | 9422 | 7520 (7350, 7609) | 1902 (1732, 1991) | 11.7 (10.6, 12.2) | 25.3 (22.8, 27.8) |
| Race/ethnicity ^f^ |  |  |  |  |  |
| NHW | 15999 | 12423 (12205, 12537) | 3576 (3357, 3689) | 18.2 (17.1, 18.8) | 28.8 (26.8, 30.8) |
| NHB | 2081 | 1459 (1384, 1499) | 622 (547, 662) | 15.5 (13.6, 16.5) | 42.6 (36.6, 48.8) |
| Hispanic | 1283 | 1406 (1332, 1445) | -123 (-196, -83) | -2.0 (-3.2, -1.4) | -8.7 (-13.7, -3.7) |
| Wave ^g^ |  |  |  |  |  |
| Wave I | 2105 | 2074 (1985, 2122) | 31 (-59, 78) | 0.1 (-0.2, 0.2) | 1.5 (-2.8, 5.9) |
| Wave II | 2349 | 2094 (2004, 2141) | 255 (166, 303) | 0.8 (0.5, 0.9) | 12.2 (7.7, 16.8) |
| Wave III | 5856 | 4885 (4748, 4957) | 971 (834, 1043) | 2.9 (2.5, 3.1) | 19.9 (16.8, 23.0) |
| Wave IV | 3436 | 2361 (2266, 2411) | 1075 (980, 1126) | 3.2 (3.0, 3.4) | 45.5 (40.7, 50.4) |
| Wave V | 4277 | 3014 (2907, 3071) | 1263 (1155, 1319) | 3.8 (3.5, 4.0) | 41.9 (37.7, 46.2) |
| Wave VI | 2510 | 1622 (1543, 1664) | 888 (809, 930) | 2.7 (2.4, 2.8) | 54.7 (48.8, 60.9) |
| **CRC** | - | - | - | - | - |
| Age |  |  |  |  |  |
| 20-64 years | 47489 | 45910 (45430, 46157) | 1579 (1099, 1826) | 8.1 (5.7, 9.4) | 3.4 (2.5, 4.4) |
| 65-84 years | 80438 | 74349 (73795, 74634) | 6089 (5534, 6374) | 130.7 (118.8, 136.8) | 8.2 (7.4, 8.9) |
| Sex |  |  |  |  |  |
| Female | 75149 | 73617 (73020, 73923) | 1532 (936, 1839) | 9.2 (5.6, 11.0) | 2.1 (1.4, 2.8) |
| Male | 87824 | 84012 (83444, 84304) | 3812 (3244, 4104) | 23.4 (19.9, 25.1) | 4.5 (3.8, 5.2) |
| Race/ethnicity ^f^ |  |  |  |  |  |
| NHW | 119910 | 115349 (114639, 115714) | 4561 (3850, 4925) | 23.3 (19.6, 25.1) | 4.0 (3.4, 4.5) |
| NHB | 21136 | 20725 (20443, 20871) | 411 (129, 557) | 10.2 (3.2, 13.9) | 2.0 (0.6, 3.4) |
| Hispanic | 13527 | 12722 (12481, 12847) | 805 (564, 930) | 13.2 (9.3, 15.3) | 6.3 (4.5, 8.1) |
| Wave ^g^ |  |  |  |  |  |
| Wave I | 16586 | 16296 (16032, 16433) | 290 (26, 426) | 0.9 (0.1, 1.3) | 1.8 (0.2, 3.3) |
| Wave II | 20033 | 19757 (19466, 19907) | 276 (-14, 427) | 0.8 (0.0, 1.3) | 1.4 (0.0, 2.8) |
| Wave III | 46842 | 45190 (44751, 45416) | 1652 (1212, 1878) | 5.0 (3.7, 5.7) | 3.7 (2.7, 4.6) |
| Wave IV | 27015 | 26089 (25756, 26262) | 926 (592, 1098) | 2.8 (1.8, 3.3) | 3.5 (2.3, 4.8) |
| Wave V | 32039 | 31042 (30678, 31230) | 997 (633, 1185) | 3.0 (1.9, 3.6) | 3.2 (2.1, 4.3) |
| Wave VI | 20458 | 19909 (19618, 20060) | 549 (257, 700) | 1.7 (0.8, 2.1) | 2.8 (1.4, 4.2) |

Abbreviations: CI, confidence interval; GI, gastrointestinal; NOS, not otherwise specified; *C. difficile*, *Clostridium difficile*; CRC, colorectal cancer; NHW, non-Hispanic White inhabitants; NHB, non-Hispanic Black inhabitants.

^a^ Sensitivity analysis was conducted by removing January 2018 and February 2018.

^b^ The contributing cause of death was adopted.

^c^ Excess death number estimated by subtract the expected number from the observed number of death.

^d^ Excess mortality per 1,000,000 persons was estimated via the excess death number divided by population size.

^e^ Excess risk was calculated as the ratio of the excess-to-expected number of death.

^f^ Non-Hispanic unknown and non-Hispanic AIAN was excluded when stratified by race/ethnicity.

^g^ Waves were identified according to the weekly surveillance of COVID-19 deaths in the US. Wave I was from March 2020 to June 2020, Wave II was from June 2020 to October 2020, Wave III was from October 2020 to June 2021, Wave IV was from June 2021 to November 2021, Wave V was from November 2021 to May 2022 and Wave VI was from May 2022 to September 2022.

# Table S23. Sensitivity analysis of excess mortality associated with selected liver and pancreatic diseases stratified by demographic factor and pandemic wave. ^a^ (United States. 2020-2022)

| **Cause of death ^b^** | **Observed deaths, No.** | **Expected deaths,**  **No. (95% CI)** | **Excess deaths,**  **No. (95% CI) ^c^** | **Excess mortality**  **(95% CI) ^d^** | **Excess risk % (95% CI) ^e^** |
| --- | --- | --- | --- | --- | --- |
| **ALD** | | | | | |
| Age |  |  |  |  |  |
| 20-64 years | 76511 | 62786 (62278, 63047) | 13725 (13217, 13986) | 70.8 (68.2, 72.2) | 21.9 (21.0, 22.7) |
| 65-84 years | 27348 | 23444 (23121, 23611) | 3904 (3581, 4070) | 83.8 (76.9, 87.4) | 16.7 (15.3, 18.0) |
| Sex |  |  |  |  |  |
| Female | 32163 | 26975 (26653, 27141) | 5188 (4866, 5354) | 31.2 (29.2, 32.2) | 19.2 (17.9, 20.5) |
| Male | 73449 | 61502 (61016, 61752) | 11947 (11461, 12197) | 73.2 (70.2, 74.7) | 19.4 (18.6, 20.3) |
| Race/ethnicity ^f^ |  |  |  |  |  |
| NHW | 72923 | 62543 (62052, 62795) | 10380 (9890, 10632) | 53.0 (50.5, 54.2) | 16.6 (15.8, 17.4) |
| NHB | 8472 | 6621 (6457, 6707) | 1851 (1687, 1936) | 46.0 (42.0, 48.2) | 28.0 (25.2, 30.7) |
| Hispanic | 16669 | 13924 (13683, 14048) | 2745 (2505, 2870) | 45.1 (41.2, 47.2) | 19.7 (17.9, 21.5) |
| Wave ^g^ |  |  |  |  |  |
| Wave I | 9457 | 8684 (8501, 8779) | 773 (591, 868) | 2.3 (1.8, 2.6) | 8.9 (6.7, 11.1) |
| Wave II | 13166 | 10621 (10419, 10726) | 2545 (2343, 2650) | 7.7 (7.1, 8.0) | 24.0 (21.9, 26.1) |
| Wave III | 31286 | 25085 (24775, 25246) | 6201 (5890, 6361) | 18.7 (17.7, 19.2) | 24.7 (23.3, 26.1) |
| Wave IV | 18434 | 14682 (14444, 14805) | 3752 (3515, 3876) | 11.3 (10.6, 11.7) | 25.6 (23.7, 27.4) |
| Wave V | 20945 | 18019 (17756, 18155) | 2926 (2663, 3062) | 8.8 (8.0, 9.2) | 16.2 (14.7, 17.8) |
| Wave VI | 12324 | 11622 (11410, 11731) | 702 (491, 812) | 2.1 (1.5, 2.4) | 6.0 (4.2, 7.9) |
| **Chronic hepatitis C** | | | | | |
| Age |  |  |  |  |  |
| 20-64 years | 16749 | 14950 (14701, 15079) | 1799 (1551, 1928) | 9.3 (8.0, 9.9) | 12.0 (10.3, 13.7) |
| 65-84 years | 14793 | 12912 (12673, 13036) | 1881 (1642, 2005) | 40.4 (35.2, 43.0) | 14.6 (12.7, 16.4) |
| Sex |  |  |  |  |  |
| Female | 10436 | 9092 (8905, 9189) | 1344 (1157, 1441) | 8.1 (6.9, 8.7) | 14.8 (12.6, 17.0) |
| Male | 25104 | 22270 (21977, 22421) | 2834 (2542, 2986) | 17.4 (15.6, 18.3) | 12.7 (11.3, 14.1) |
| Race/ethnicity ^f^ |  |  |  |  |  |
| NHW | 22453 | 20147 (19869, 20291) | 2306 (2028, 2450) | 11.8 (10.3, 12.5) | 11.4 (10.0, 12.9) |
| NHB | 6393 | 5420 (5276, 5496) | 973 (828, 1048) | 24.2 (20.6, 26.1) | 18.0 (15.1, 20.9) |
| Hispanic | 4488 | 3588 (3468, 3651) | 900 (780, 963) | 14.8 (12.8, 15.8) | 25.1 (21.5, 28.8) |
| Wave ^g^ |  |  |  |  |  |
| Wave I | 4065 | 3644 (3526, 3707) | 421 (302, 483) | 1.3 (0.9, 1.5) | 11.6 (8.2, 15.0) |
| Wave II | 4731 | 4083 (3958, 4149) | 648 (522, 714) | 2.0 (1.6, 2.2) | 15.9 (12.6, 19.2) |
| Wave III | 10560 | 9344 (9154, 9442) | 1216 (1027, 1315) | 3.7 (3.1, 4.0) | 13.0 (10.9, 15.2) |
| Wave IV | 5715 | 4941 (4804, 5014) | 774 (636, 846) | 2.3 (1.9, 2.5) | 15.7 (12.7, 18.7) |
| Wave V | 6708 | 5935 (5784, 6014) | 773 (622, 852) | 2.3 (1.9, 2.6) | 13.0 (10.3, 15.7) |
| Wave VI | 3761 | 3497 (3381, 3558) | 264 (148, 325) | 0.8 (0.4, 1.0) | 7.5 (4.1, 11.0) |
| **Fibrosis/cirrhosis** | | | | | |
| Age |  |  |  |  |  |
| 20-64 years | 55791 | 49329 (48813, 49595) | 6462 (5945, 6727) | 33.3 (30.7, 34.7) | 13.1 (12.2, 14.0) |
| 65-84 years | 70205 | 66419 (65914, 66678) | 3786 (3281, 4046) | 81.3 (70.4, 86.8) | 5.7 (4.9, 6.5) |
| Sex |  |  |  |  |  |
| Female | 58190 | 54213 (53756, 54448) | 3977 (3521, 4212) | 23.9 (21.1, 25.3) | 7.3 (6.5, 8.2) |
| Male | 78807 | 73880 (73347, 74154) | 4927 (4394, 5201) | 30.2 (26.9, 31.9) | 6.7 (5.9, 7.4) |
| Race/ethnicity ^f^ |  |  |  |  |  |
| NHW | 97028 | 92410 (91815, 92716) | 4618 (4022, 4924) | 23.6 (20.5, 25.1) | 5.0 (4.3, 5.7) |
| NHB | 11965 | 11262 (11033, 11380) | 703 (475, 822) | 17.5 (11.8, 20.4) | 6.2 (4.3, 8.2) |
| Hispanic | 20576 | 17081 (16818, 17217) | 3495 (3232, 3631) | 57.5 (53.2, 59.7) | 20.5 (18.8, 22.1) |
| Wave ^g^ |  |  |  |  |  |
| Wave I | 12871 | 12718 (12492, 12835) | 153 (-72, 270) | 0.5 (-0.2, 0.8) | 1.2 (-0.5, 3.0) |
| Wave II | 16352 | 14913 (14669, 15039) | 1439 (1195, 1566) | 4.3 (3.6, 4.7) | 9.6 (8.0, 11.3) |
| Wave III | 39900 | 36594 (36212, 36791) | 3306 (2924, 3503) | 10.0 (8.8, 10.6) | 9.0 (8.0, 10.1) |
| Wave IV | 23043 | 20901 (20612, 21050) | 2142 (1854, 2292) | 6.5 (5.6, 6.9) | 10.2 (8.8, 11.7) |
| Wave V | 28129 | 26786 (26459, 26955) | 1343 (1016, 1511) | 4.0 (3.1, 4.6) | 5.0 (3.8, 6.2) |
| Wave VI | 16702 | 16654 (16397, 16788) | 48 (-210, 181) | 0.1 (-0.6, 0.5) | 0.3 (-1.2, 1.8) |
| **Hepatic failure** | | | | | |
| Age |  |  |  |  |  |
| 20-64 years | 39474 | 35223 (34828, 35427) | 4251 (3855, 4455) | 21.9 (19.9, 23.0) | 12.1 (11.0, 13.2) |
| 65-84 years | 33526 | 32631 (32255, 32824) | 895 (520, 1089) | 19.2 (11.2, 23.4) | 2.7 (1.6, 3.8) |
| Sex |  |  |  |  |  |
| Female | 34853 | 32886 (32531, 33069) | 1967 (1611, 2150) | 11.8 (9.7, 12.9) | 6.0 (4.9, 7.1) |
| Male | 44412 | 41758 (41358, 41965) | 2654 (2253, 2860) | 16.3 (13.8, 17.5) | 6.4 (5.4, 7.3) |
| Race/ethnicity ^f^ |  |  |  |  |  |
| NHW | 54979 | 52202 (51754, 52432) | 2777 (2329, 3007) | 14.2 (11.9, 15.3) | 5.3 (4.4, 6.2) |
| NHB | 8881 | 8051 (7876, 8143) | 830 (654, 921) | 20.6 (16.3, 22.9) | 10.3 (8.0, 12.6) |
| Hispanic | 10343 | 9241 (9052, 9339) | 1102 (914, 1200) | 18.1 (15.0, 19.7) | 11.9 (9.8, 14.1) |
| Wave ^g^ |  |  |  |  |  |
| Wave I | 7433 | 7695 (7522, 7785) | -262 (-435, -171) | -0.8 (-1.3, -0.5) | -3.4 (-5.6, -1.2) |
| Wave II | 9599 | 8954 (8767, 9051) | 645 (458, 742) | 1.9 (1.4, 2.2) | 7.2 (5.1, 9.4) |
| Wave III | 23338 | 21487 (21198, 21637) | 1851 (1562, 2000) | 5.6 (4.7, 6.0) | 8.6 (7.2, 10.0) |
| Wave IV | 13496 | 12157 (11939, 12270) | 1339 (1121, 1452) | 4.0 (3.4, 4.4) | 11.0 (9.1, 12.9) |
| Wave V | 15832 | 15239 (14995, 15365) | 593 (349, 719) | 1.8 (1.1, 2.2) | 3.9 (2.3, 5.5) |
| Wave VI | 9567 | 9406 (9215, 9506) | 161 (-31, 260) | 0.5 (-0.1, 0.8) | 1.7 (-0.3, 3.8) |
| **Acute pancreatitis** | | | | | |
| Age |  |  |  |  |  |
| 20-64 years | 5174 | 3975 (3751, 4092) | 1199 (974, 1315) | 6.2 (5.0, 6.8) | 30.2 (26.6, 33.7) |
| 65-84 years | 6078 | 4079 (3815, 4216) | 1999 (1735, 2135) | 42.9 (37.2, 45.8) | 49.0 (45.3, 52.8) |
| Sex |  |  |  |  |  |
| Female | 7323 | 5887 (5725, 5972) | 1436 (1273, 1520) | 8.6 (7.6, 9.1) | 24.4 (21.6, 27.3) |
| Male | 10974 | 9257 (9041, 9370) | 1717 (1500, 1829) | 10.5 (9.2, 11.2) | 18.5 (16.3, 20.8) |
| Race/ethnicity ^f^ |  |  |  |  |  |
| NHW | 13166 | 10809 (10587, 10924) | 2357 (2135, 2472) | 12.0 (10.9, 12.6) | 21.8 (19.7, 23.9) |
| NHB | 2382 | 1913 (1827, 1959) | 469 (383, 515) | 11.7 (9.5, 12.8) | 24.5 (19.6, 29.6) |
| Hispanic | 1582 | 1723 (1641, 1766) | -141 (-222, -97) | -2.3 (-3.7, -1.6) | -8.2 (-12.7, -3.6) |
| Wave ^g^ |  |  |  |  |  |
| Wave I | 1806 | 1556 (1475, 1600) | 250 (169, 293) | 0.8 (0.5, 0.9) | 16.1 (10.8, 21.5) |
| Wave II | 2264 | 1820 (1732, 1866) | 444 (357, 491) | 1.3 (1.1, 1.5) | 24.4 (19.3, 29.6) |
| Wave III | 5365 | 4362 (4226, 4433) | 1003 (868, 1075) | 3.0 (2.6, 3.2) | 23.0 (19.7, 26.3) |
| Wave IV | 2994 | 2446 (2344, 2500) | 548 (447, 602) | 1.7 (1.3, 1.8) | 22.4 (18.1, 26.8) |
| Wave V | 3647 | 3105 (2990, 3165) | 542 (428, 603) | 1.6 (1.3, 1.8) | 17.5 (13.7, 21.3) |
| Wave VI | 2221 | 1900 (1811, 1948) | 321 (231, 368) | 1.0 (0.7, 1.1) | 16.9 (12.1, 21.8) |

Abbreviations: CI, confidence interval; ALD, alcoholic liver disease NHW, non-Hispanic White inhabitants; NHB, non-Hispanic Black inhabitants.

^a^ Sensitivity analysis was conducted by removing January 2018 and February 2018.

^b^ The contributing cause of death was adopted.

^c^ Excess death number estimated by subtract the expected number from the observed number of death.

^d^ Excess mortality per 1,000,000 persons was estimated via the excess death number divided by population size.

^e^ Excess risk was calculated as the ratio of the excess-to-expected number of death.

^f^ Non-Hispanic unknown and non-Hispanic AIAN was excluded when stratified by race/ethnicity.

^g^ Waves were identified according to the weekly surveillance of COVID-19 deaths in the US. Wave I was from March 2020 to June 2020, Wave II was from June 2020 to October 2020, Wave III was from October 2020 to June 2021, Wave IV was from June 2021 to November 2021, Wave V was from November 2021 to May 2022 and Wave VI was from May 2022 to September 2022.

# Table S24. Sensitivity analysis of excess mortality associated with selected gastrointestinal diseases stratified by demographic factor and pandemic wave. ^a^ (United States. 2020-2022)

| **Cause of death ^b^** | **Observed deaths, No.** | **Expected deaths,**  **No. (95% CI)** | **Excess deaths,**  **No. (95% CI) ^c^** | **Excess mortality**  **(95% CI) ^d^** | **Excess risk % (95% CI) ^e^** |
| --- | --- | --- | --- | --- | --- |
| **GI hemorrhage, NOS** | | | | | |
| Age |  |  |  |  |  |
| 20-64 years | 29214 | 21306 (20960, 21484) | 7908 (7562, 8087) | 40.8 (39.0, 41.7) | 37.1 (35.5, 38.7) |
| 65-84 years | 51555 | 40895 (40495, 41101) | 10660 (10260, 10866) | 228.8 (220.2, 233.2) | 26.1 (25.0, 27.2) |
| Sex |  |  |  |  |  |
| Female | 49701 | 40102 (39687, 40316) | 9599 (9184, 9813) | 57.6 (55.2, 58.9) | 23.9 (22.8, 25.0) |
| Male | 61968 | 49600 (49076, 49870) | 12368 (11844, 12637) | 75.8 (72.6, 77.4) | 24.9 (24.0, 25.9) |
| Race/ethnicity ^f^ |  |  |  |  |  |
| NHW | 81314 | 67486 (66906, 67784) | 13828 (13248, 14126) | 70.5 (67.6, 72.1) | 20.5 (19.7, 21.3) |
| NHB | 13875 | 10484 (10259, 10601) | 3391 (3165, 3508) | 84.4 (78.7, 87.3) | 32.3 (30.2, 34.6) |
| Hispanic | 10322 | 6758 (6597, 6842) | 3564 (3403, 3648) | 58.6 (56.0, 60.0) | 52.7 (49.8, 55.7) |
| Wave ^g^ |  |  |  |  |  |
| Wave I | 10532 | 9512 (9301, 9622) | 1020 (809, 1129) | 3.1 (2.4, 3.4) | 10.7 (8.6, 12.8) |
| Wave II | 13288 | 10673 (10449, 10789) | 2615 (2391, 2731) | 7.9 (7.2, 8.2) | 24.5 (22.4, 26.6) |
| Wave III | 33477 | 26343 (25992, 26524) | 7134 (6783, 7315) | 21.5 (20.4, 22.0) | 27.1 (25.7, 28.4) |
| Wave IV | 18776 | 14290 (14031, 14424) | 4486 (4227, 4620) | 13.5 (12.7, 13.9) | 31.4 (29.5, 33.3) |
| Wave V | 22879 | 18424 (18130, 18576) | 4455 (4161, 4607) | 13.4 (12.5, 13.9) | 24.2 (22.6, 25.8) |
| Wave VI | 12717 | 10817 (10592, 10934) | 1900 (1675, 2017) | 5.7 (5.0, 6.1) | 17.6 (15.5, 19.6) |
| **Ulcers** | | | | | |
| Age |  |  |  |  |  |
| 20-64 years | 3156 | 2539 (2377, 2624) | 617 (454, 701) | 3.2 (2.3, 3.6) | 24.3 (20.0, 28.7) |
| 65-84 years | 10705 | 9676 (9373, 9832) | 1029 (726, 1185) | 22.1 (15.6, 25.4) | 10.6 (8.5, 12.7) |
| Sex |  |  |  |  |  |
| Female | 9976 | 8890 (8704, 8988) | 1086 (899, 1183) | 6.5 (5.4, 7.1) | 12.2 (10.0, 14.4) |
| Male | 11334 | 9371 (9171, 9475) | 1963 (1763, 2067) | 12.0 (10.8, 12.7) | 20.9 (18.7, 23.2) |
| Race/ethnicity ^f^ |  |  |  |  |  |
| NHW | 15851 | 13905 (13674, 14025) | 1946 (1715, 2066) | 9.9 (8.7, 10.5) | 14.0 (12.2, 15.8) |
| NHB | 2216 | 1775 (1693, 1819) | 441 (358, 485) | 11.0 (8.9, 12.1) | 24.8 (19.7, 30.1) |
| Hispanic | 1676 | 1458 (1383, 1498) | 218 (143, 258) | 3.6 (2.4, 4.2) | 15.0 (9.5, 20.5) |
| Wave ^g^ |  |  |  |  |  |
| Wave I | 2059 | 1953 (1865, 1999) | 106 (19, 153) | 0.3 (0.1, 0.5) | 5.4 (0.9, 10.0) |
| Wave II | 2478 | 2053 (1963, 2101) | 425 (335, 473) | 1.3 (1.0, 1.4) | 20.7 (16.0, 25.5) |
| Wave III | 6201 | 5417 (5271, 5494) | 784 (638, 860) | 2.4 (1.9, 2.6) | 14.5 (11.6, 17.3) |
| Wave IV | 3559 | 2863 (2757, 2919) | 696 (590, 752) | 2.1 (1.8, 2.3) | 24.3 (20.3, 28.4) |
| Wave V | 4481 | 3897 (3773, 3962) | 584 (461, 649) | 1.8 (1.4, 2.0) | 15.0 (11.6, 18.4) |
| Wave VI | 2532 | 2145 (2053, 2193) | 387 (296, 436) | 1.2 (0.9, 1.3) | 18.0 (13.5, 22.7) |
| ***C. difficile* colitis** | | | | | |
| Age |  |  |  |  |  |
| 20-64 years | 1250 | 843 (774, 881) | 407 (337, 444) | 2.1 (1.7, 2.3) | 48.3 (40.2, 56.6) |
| 65-84 years | 11177 | 7977 (7770, 8085) | 3200 (2992, 3308) | 68.7 (64.2, 71.0) | 40.1 (37.5, 42.7) |
| Sex |  |  |  |  |  |
| Female | 11111 | 8014 (7838, 8105) | 3097 (2922, 3189) | 18.6 (17.5, 19.2) | 38.6 (36.1, 41.2) |
| Male | 9422 | 7123 (6957, 7209) | 2299 (2134, 2386) | 14.1 (13.1, 14.6) | 32.3 (29.6, 35.0) |
| Race/ethnicity ^f^ |  |  |  |  |  |
| NHW | 15999 | 11670 (11441, 11789) | 4329 (4100, 4448) | 22.1 (20.9, 22.7) | 37.1 (35.0, 39.2) |
| NHB | 2081 | 1389 (1316, 1428) | 692 (619, 731) | 17.2 (15.4, 18.2) | 49.8 (43.5, 56.3) |
| Hispanic | 1283 | 1302 (1231, 1340) | -19 (-90, 19) | -0.3 (-1.5, 0.3) | -1.5 (-6.8, 4.0) |
| Wave ^g^ |  |  |  |  |  |
| Wave I | 2105 | 1995 (1907, 2042) | 110 (22, 157) | 0.3 (0.1, 0.5) | 5.5 (1.1, 10.1) |
| Wave II | 2349 | 2008 (1920, 2055) | 341 (253, 388) | 1.0 (0.8, 1.2) | 17.0 (12.3, 21.8) |
| Wave III | 5856 | 4658 (4524, 4729) | 1198 (1063, 1268) | 3.6 (3.2, 3.8) | 25.7 (22.5, 29.0) |
| Wave IV | 3436 | 2203 (2111, 2252) | 1233 (1141, 1282) | 3.7 (3.4, 3.9) | 56.0 (50.8, 61.2) |
| Wave V | 4277 | 2813 (2709, 2868) | 1464 (1360, 1519) | 4.4 (4.1, 4.6) | 52.0 (47.5, 56.6) |
| Wave VI | 2510 | 1471 (1395, 1511) | 1039 (964, 1080) | 3.1 (2.9, 3.3) | 70.6 (64.0, 77.4) |
| **Colorectal cancer** | | | | | |
| Age |  |  |  |  |  |
| 20-64 years | 47489 | 46704 (46199, 46963) | 785 (281, 1045) | 4.0 (1.4, 5.4) | 1.7 (0.8, 2.6) |
| 65-84 years | 80438 | 74494 (73924, 74787) | 5944 (5374, 6237) | 127.6 (115.3, 133.9) | 8.0 (7.2, 8.7) |
| Sex |  |  |  |  |  |
| Female | 75149 | 72730 (72116, 73046) | 2419 (1805, 2734) | 14.5 (10.8, 16.4) | 3.3 (2.6, 4.1) |
| Male | 87824 | 84657 (84087, 84950) | 3167 (2597, 3460) | 19.4 (15.9, 21.2) | 3.7 (3.1, 4.4) |
| Race/ethnicity ^f^ |  |  |  |  |  |
| NHW | 119910 | 115754 (115026, 116128) | 4156 (3428, 4529) | 21.2 (17.5, 23.1) | 3.6 (3.0, 4.2) |
| NHB | 21136 | 20053 (19775, 20196) | 1083 (806, 1227) | 26.9 (20.1, 30.5) | 5.4 (4.0, 6.8) |
| Hispanic | 13527 | 12850 (12597, 12981) | 677 (425, 808) | 11.1 (7.0, 13.3) | 5.3 (3.5, 7.0) |
| Wave ^g^ |  |  |  |  |  |
| Wave I | 16586 | 16272 (15988, 16419) | 314 (30, 461) | 0.9 (0.1, 1.4) | 1.9 (0.4, 3.5) |
| Wave II | 20033 | 19699 (19387, 19861) | 334 (21, 495) | 1.0 (0.1, 1.5) | 1.7 (0.3, 3.1) |
| Wave III | 46842 | 45200 (44727, 45443) | 1642 (1169, 1885) | 4.9 (3.5, 5.7) | 3.6 (2.7, 4.6) |
| Wave IV | 27015 | 26104 (25744, 26289) | 911 (552, 1097) | 2.7 (1.7, 3.3) | 3.5 (2.3, 4.7) |
| Wave V | 32039 | 30922 (30531, 31124) | 1117 (726, 1318) | 3.4 (2.2, 4.0) | 3.6 (2.5, 4.7) |
| Wave VI | 20458 | 19840 (19527, 20002) | 618 (304, 780) | 1.9 (0.9, 2.4) | 3.1 (1.7, 4.5) |

Abbreviations: CI, confidence interval; GI, gastrointestinal; NOS, not otherwise specified; *C. difficile*, *Clostridium difficile*; NHW, non-Hispanic White inhabitants; NHB, non-Hispanic Black inhabitants.

^a^ Sensitivity analysis was conducted with harmonics = 8.

^b^ The contributing cause of death was adopted.

^c^ Excess death number estimated by subtract the expected number from the observed number of death.

^d^ Excess mortality per 1,000,000 persons was estimated via the excess death number divided by population size.

^e^ Excess risk was calculated as the ratio of the excess-to-expected number of death.

^f^ Non-Hispanic unknown and non-Hispanic AIAN was excluded when stratified by race/ethnicity.

^g^ Waves were identified according to the weekly surveillance of COVID-19 deaths in the US. Wave I was from March 2020 to June 2020, Wave II was from June 2020 to October 2020, Wave III was from October 2020 to June 2021, Wave IV was from June 2021 to November 2021, Wave V was from November 2021 to May 2022 and Wave VI was from May 2022 to September 2022.

# Table S25. Sensitivity analysis of excess mortality associated with selected liver and pancreatic diseases stratified by demographic factor and pandemic wave. ^a^ (United States. 2020-2022)

| **Cause of death ^b^** | **Observed deaths, No.** | **Expected deaths,**  **No. (95% CI)** | **Excess deaths,**  **No. (95% CI) ^c^** | **Excess mortality**  **(95% CI) ^d^** | **Excess risk % (95% CI) ^e^** |
| --- | --- | --- | --- | --- | --- |
| **Alcoholic liver disease** | | | | | |
| Age |  |  |  |  |  |
| 20-64 years | 76511 | 62646 (62147, 62903) | 13865 (13365, 14122) | 71.5 (68.9, 72.9) | 22.1 (21.3, 23.0) |
| 65-84 years | 27348 | 23411 (23080, 23582) | 3937 (3606, 4108) | 84.5 (77.4, 88.2) | 16.8 (15.4, 18.2) |
| Sex |  |  |  |  |  |
| Female | 32163 | 26526 (26207, 26691) | 5637 (5318, 5802) | 33.9 (31.9, 34.8) | 21.3 (19.9, 22.6) |
| Male | 73449 | 61581 (61088, 61834) | 11868 (11376, 12122) | 72.7 (69.7, 74.3) | 19.3 (18.4, 20.1) |
| Race/ethnicity ^f^ |  |  |  |  |  |
| NHW | 72923 | 62660 (62169, 62912) | 10263 (9772, 10515) | 52.4 (49.9, 53.6) | 16.4 (15.5, 17.2) |
| NHB | 8472 | 6733 (6567, 6820) | 1739 (1573, 1826) | 43.3 (39.1, 45.4) | 25.8 (23.2, 28.5) |
| Hispanic | 16669 | 13477 (13242, 13599) | 3192 (2957, 3314) | 52.5 (48.6, 54.5) | 23.7 (21.8, 25.6) |
| Wave ^g^ |  |  |  |  |  |
| Wave I | 9457 | 8665 (8481, 8761) | 792 (608, 888) | 2.4 (1.8, 2.7) | 9.1 (7.0, 11.4) |
| Wave II | 13166 | 10572 (10369, 10678) | 2594 (2390, 2700) | 7.8 (7.2, 8.1) | 24.5 (22.4, 26.7) |
| Wave III | 31286 | 25027 (24714, 25189) | 6259 (5946, 6420) | 18.9 (17.9, 19.3) | 25.0 (23.6, 26.4) |
| Wave IV | 18434 | 14655 (14416, 14779) | 3779 (3539, 3903) | 11.4 (10.7, 11.8) | 25.8 (24.0, 27.6) |
| Wave V | 20945 | 17900 (17635, 18037) | 3045 (2780, 3182) | 9.2 (8.4, 9.6) | 17.0 (15.4, 18.6) |
| Wave VI | 12324 | 11547 (11334, 11657) | 777 (564, 887) | 2.3 (1.7, 2.7) | 6.7 (4.9, 8.6) |
| **Chronic hepatitis C** | | | | | |
| Age |  |  |  |  |  |
| 20-64 years | 16749 | 14785 (14525, 14920) | 1964 (1704, 2099) | 10.1 (8.8, 10.8) | 13.3 (11.6, 15.0) |
| 65-84 years | 14793 | 12670 (12394, 12812) | 2123 (1848, 2266) | 45.6 (39.7, 48.6) | 16.8 (14.9, 18.6) |
| Sex |  |  |  |  |  |
| Female | 10436 | 8479 (8298, 8573) | 1957 (1777, 2051) | 11.8 (10.7, 12.3) | 23.1 (20.7, 25.5) |
| Male | 25104 | 22397 (22104, 22549) | 2707 (2414, 2859) | 16.6 (14.8, 17.5) | 12.1 (10.7, 13.5) |
| Race/ethnicity ^f^ |  |  |  |  |  |
| NHW | 22453 | 19787 (19495, 19938) | 2666 (2374, 2817) | 13.6 (12.1, 14.4) | 13.5 (12.0, 15.0) |
| NHB | 6393 | 5413 (5269, 5488) | 980 (836, 1056) | 24.4 (20.8, 26.3) | 18.1 (15.2, 21.0) |
| Hispanic | 4488 | 3518 (3391, 3584) | 970 (844, 1037) | 16.0 (13.9, 17.1) | 27.6 (23.9, 31.3) |
| Wave ^g^ |  |  |  |  |  |
| Wave I | 4065 | 3609 (3484, 3675) | 456 (330, 521) | 1.4 (1.0, 1.6) | 12.6 (9.2, 16.1) |
| Wave II | 4731 | 4041 (3909, 4111) | 690 (557, 759) | 2.1 (1.7, 2.3) | 17.1 (13.8, 20.4) |
| Wave III | 10560 | 9222 (9022, 9326) | 1338 (1138, 1442) | 4.0 (3.4, 4.3) | 14.5 (12.3, 16.7) |
| Wave IV | 5715 | 4845 (4700, 4920) | 870 (725, 946) | 2.6 (2.2, 2.9) | 18.0 (14.9, 21.0) |
| Wave V | 6708 | 5822 (5663, 5905) | 886 (727, 969) | 2.7 (2.2, 2.9) | 15.2 (12.5, 18.0) |
| Wave VI | 3761 | 3417 (3296, 3481) | 344 (222, 408) | 1.0 (0.7, 1.2) | 10.1 (6.6, 13.6) |
| **Fibrosis/cirrhosis** | | | | | |
| Age |  |  |  |  |  |
| 20-64 years | 55791 | 48304 (47737, 48595) | 7487 (6920, 7778) | 38.6 (35.7, 40.1) | 15.5 (14.5, 16.5) |
| 65-84 years | 70205 | 65561 (65027, 65835) | 4644 (4111, 4919) | 99.7 (88.2, 105.6) | 7.1 (6.3, 7.9) |
| Sex |  |  |  |  |  |
| Female | 58190 | 53179 (52714, 53418) | 5011 (4546, 5250) | 30.1 (27.3, 31.5) | 9.4 (8.5, 10.3) |
| Male | 78807 | 72810 (72259, 73093) | 5997 (5446, 6280) | 36.7 (33.4, 38.5) | 8.2 (7.5, 9.0) |
| Race/ethnicity ^f^ |  |  |  |  |  |
| NHW | 97028 | 90560 (89912, 90893) | 6468 (5820, 6801) | 33.0 (29.7, 34.7) | 7.1 (6.5, 7.8) |
| NHB | 11965 | 11206 (10970, 11328) | 759 (523, 881) | 18.9 (13.0, 21.9) | 6.8 (4.9, 8.7) |
| Hispanic | 20576 | 16787 (16523, 16923) | 3789 (3526, 3926) | 62.3 (58.0, 64.6) | 22.6 (20.9, 24.3) |
| Wave ^g^ |  |  |  |  |  |
| Wave I | 12871 | 12580 (12317, 12716) | 291 (29, 427) | 0.9 (0.1, 1.3) | 2.3 (0.6, 4.1) |
| Wave II | 16352 | 14755 (14471, 14902) | 1597 (1312, 1744) | 4.8 (4.0, 5.3) | 10.8 (9.1, 12.5) |
| Wave III | 39900 | 36120 (35675, 36349) | 3780 (3335, 4009) | 11.4 (10.0, 12.1) | 10.5 (9.4, 11.6) |
| Wave IV | 23043 | 20514 (20179, 20687) | 2529 (2193, 2702) | 7.6 (6.6, 8.1) | 12.3 (10.9, 13.8) |
| Wave V | 28129 | 26260 (25880, 26455) | 1869 (1490, 2065) | 5.6 (4.5, 6.2) | 7.1 (5.9, 8.4) |
| Wave VI | 16702 | 16236 (15937, 16390) | 466 (168, 620) | 1.4 (0.5, 1.9) | 2.9 (1.3, 4.4) |
| **Hepatic failure** | | | | | |
| Age |  |  |  |  |  |
| 20-64 years | 39474 | 34998 (34582, 35211) | 4476 (4061, 4690) | 23.1 (21.0, 24.2) | 12.8 (11.7, 13.9) |
| 65-84 years | 33526 | 32136 (31751, 32335) | 1390 (1005, 1588) | 29.8 (21.6, 34.1) | 4.3 (3.2, 5.4) |
| Sex |  |  |  |  |  |
| Female | 34853 | 32847 (32491, 33030) | 2006 (1651, 2190) | 12.0 (9.9, 13.2) | 6.1 (5.0, 7.2) |
| Male | 44412 | 40972 (40570, 41179) | 3440 (3038, 3647) | 21.1 (18.6, 22.3) | 8.4 (7.4, 9.4) |
| Race/ethnicity ^f^ |  |  |  |  |  |
| NHW | 54979 | 51585 (51115, 51827) | 3394 (2923, 3636) | 17.3 (14.9, 18.6) | 6.6 (5.7, 7.5) |
| NHB | 8881 | 8154 (7977, 8246) | 727 (550, 819) | 18.1 (13.7, 20.4) | 8.9 (6.7, 11.2) |
| Hispanic | 10343 | 9108 (8921, 9205) | 1235 (1048, 1332) | 20.3 (17.2, 21.9) | 13.6 (11.4, 15.8) |
| Wave ^g^ |  |  |  |  |  |
| Wave I | 7433 | 7640 (7449, 7740) | -207 (-398, -108) | -0.6 (-1.2, -0.3) | -2.7 (-4.9, -0.5) |
| Wave II | 9599 | 8874 (8668, 8981) | 725 (519, 832) | 2.2 (1.6, 2.5) | 8.2 (6.0, 10.3) |
| Wave III | 23338 | 21328 (21009, 21493) | 2010 (1691, 2175) | 6.1 (5.1, 6.6) | 9.4 (8.0, 10.8) |
| Wave IV | 13496 | 11980 (11740, 12104) | 1516 (1277, 1640) | 4.6 (3.8, 4.9) | 12.7 (10.8, 14.6) |
| Wave V | 15832 | 15026 (14758, 15165) | 806 (538, 945) | 2.4 (1.6, 2.8) | 5.4 (3.7, 7.0) |
| Wave VI | 9567 | 9252 (9042, 9361) | 315 (105, 424) | 0.9 (0.3, 1.3) | 3.4 (1.3, 5.5) |
| **Acute pancreatitis** | | | | | |
| Age |  |  |  |  |  |
| 20-64 years | 5174 | 4332 (4098, 4453) | 842 (608, 964) | 4.3 (3.1, 5.0) | 19.4 (16.2, 22.7) |
| 65-84 years | 6078 | 4321 (4037, 4468) | 1757 (1473, 1904) | 37.7 (31.6, 40.9) | 40.7 (37.1, 44.2) |
| Sex |  |  |  |  |  |
| Female | 7323 | 6037 (5870, 6124) | 1286 (1120, 1373) | 7.7 (6.7, 8.2) | 21.3 (18.5, 24.1) |
| Male | 10974 | 9112 (8898, 9223) | 1862 (1648, 1973) | 11.4 (10.1, 12.1) | 20.4 (18.2, 22.7) |
| Race/ethnicity ^f^ |  |  |  |  |  |
| NHW | 13166 | 10834 (10615, 10948) | 2332 (2112, 2446) | 11.9 (10.8, 12.5) | 21.5 (19.5, 23.6) |
| NHB | 2382 | 2024 (1936, 2071) | 358 (269, 404) | 8.9 (6.7, 10.1) | 17.7 (13.0, 22.5) |
| Hispanic | 1582 | 1693 (1613, 1736) | -111 (-192, -68) | -1.8 (-3.2, -1.1) | -6.6 (-11.1, -1.9) |
| Wave ^g^ |  |  |  |  |  |
| Wave I | 1806 | 1557 (1474, 1602) | 249 (166, 293) | 0.8 (0.5, 0.9) | 16.0 (10.7, 21.4) |
| Wave II | 2264 | 1819 (1729, 1866) | 445 (355, 493) | 1.3 (1.1, 1.5) | 24.5 (19.4, 29.6) |
| Wave III | 5365 | 4358 (4219, 4431) | 1007 (867, 1080) | 3.0 (2.6, 3.3) | 23.1 (19.8, 26.4) |
| Wave IV | 2994 | 2455 (2350, 2510) | 539 (435, 595) | 1.6 (1.3, 1.8) | 22.0 (17.6, 26.4) |
| Wave V | 3647 | 3111 (2994, 3173) | 536 (418, 597) | 1.6 (1.3, 1.8) | 17.2 (13.5, 21.1) |
| Wave VI | 2221 | 1900 (1809, 1949) | 321 (229, 369) | 1.0 (0.7, 1.1) | 16.9 (12.1, 21.8) |

Abbreviations: CI, confidence interval; NHW, non-Hispanic White inhabitants; NHB, non-Hispanic Black inhabitants.

^a^ Sensitivity analysis was conducted with harmonics = 8.

^b^ The contributing cause of death was adopted.

^c^ Excess death number estimated by subtract the expected number from the observed number of death.

^d^ Excess mortality per 1,000,000 persons was estimated via the excess death number divided by population size.

^e^ Excess risk was calculated as the ratio of the excess-to-expected number of death.

^f^ Non-Hispanic unknown and non-Hispanic AIAN was excluded when stratified by race/ethnicity.

^g^ Waves were identified according to the weekly surveillance of COVID-19 deaths in the US. Wave I was from March 2020 to June 2020, Wave II was from June 2020 to October 2020, Wave III was from October 2020 to June 2021, Wave IV was from June 2021 to November 2021, Wave V was from November 2021 to May 2022 and Wave VI was from May 2022 to September 2022.

#
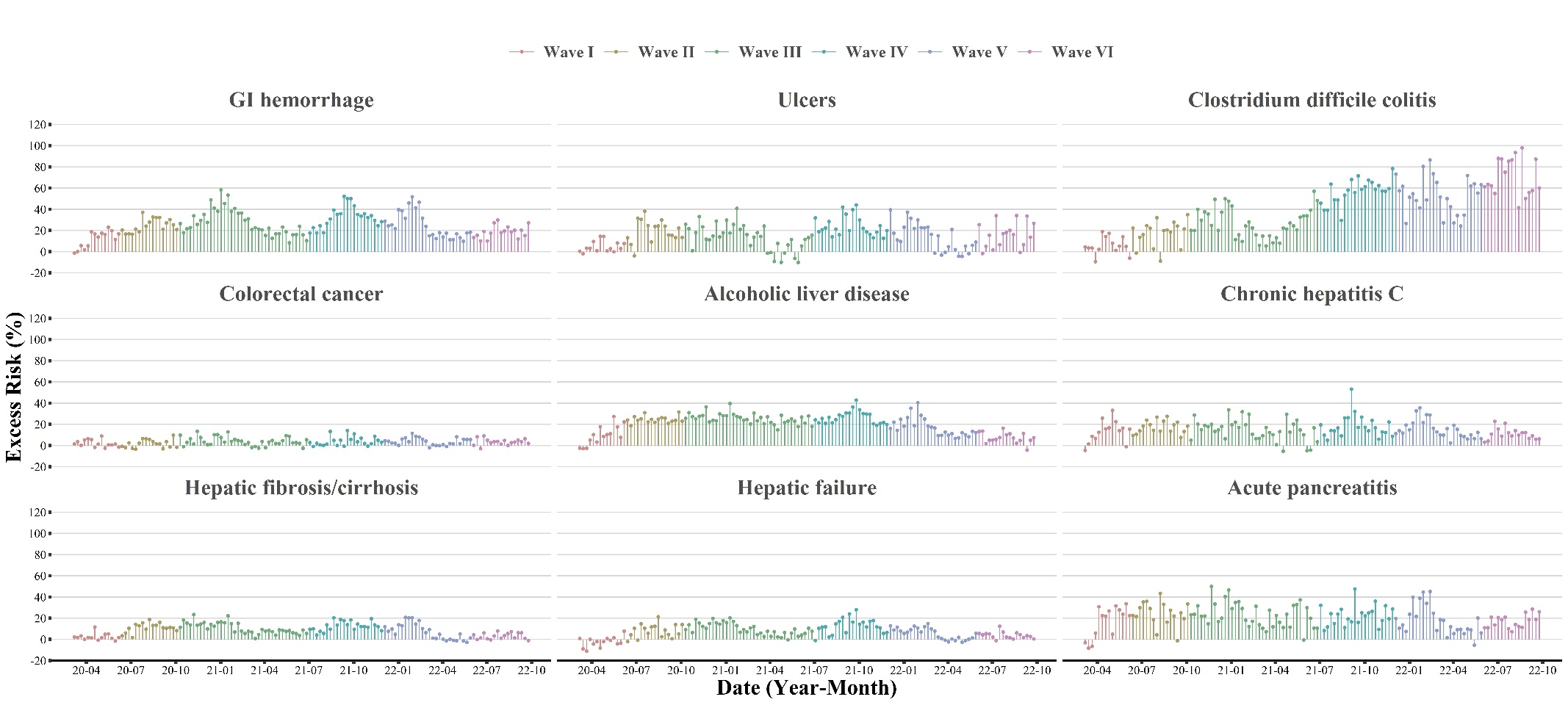
**Fig. S1 Weekly estimates of excess risks (%) associated with selected** **gastrointestinal, liver, and pancreatic diseases (United States. 2020-2022)** This figure shows the time-series estimates of excess risk (%) associated with selected gastrointestinal, liver, and pancreatic diseases from March 2020 to September 2022. The contributing cause of death was adopted.

#
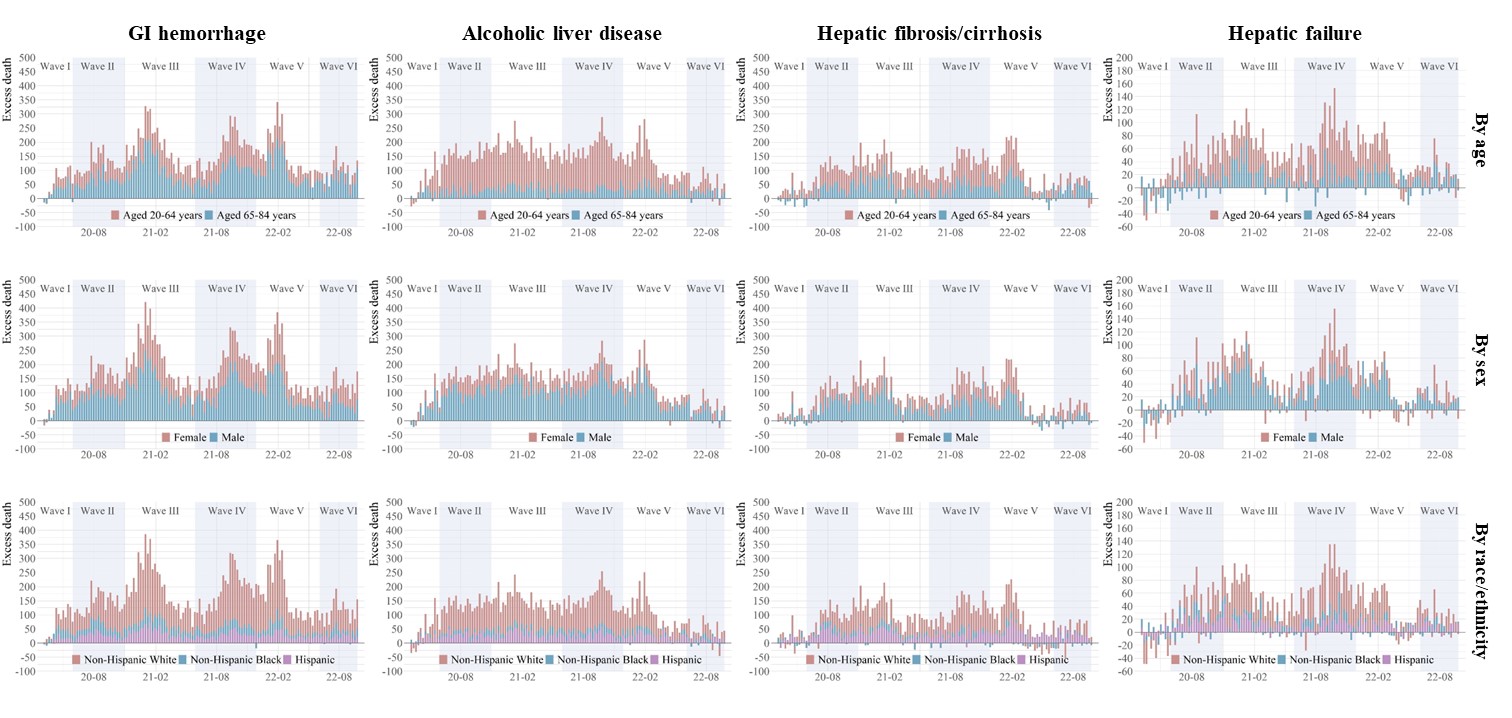
**Fig. S2 Weekly estimates of excess deaths for A) GI hemorrhage, B) Alcoholic liver disease and C) Hepatic fibrosis/cirrhosis by demographic characteristic (United States. 2020-2022)** The contributing cause of death was adopted. For each subtype, panels from left to right are weekly excess death estimates by age, sex, and racial/ethnic, respectively. For age group stratification, the estimates are shown in red for inhabitants aged 20-64 years and blue for inhabitants aged 65-84 years. For sex stratification, the estimates are shown in red for females and blue for males. For race/ethnicity stratification, the estimates are shown in red for non-Hispanic White, blue for non-Hispanic Black, and purple for Hispanic inhabitants.

#
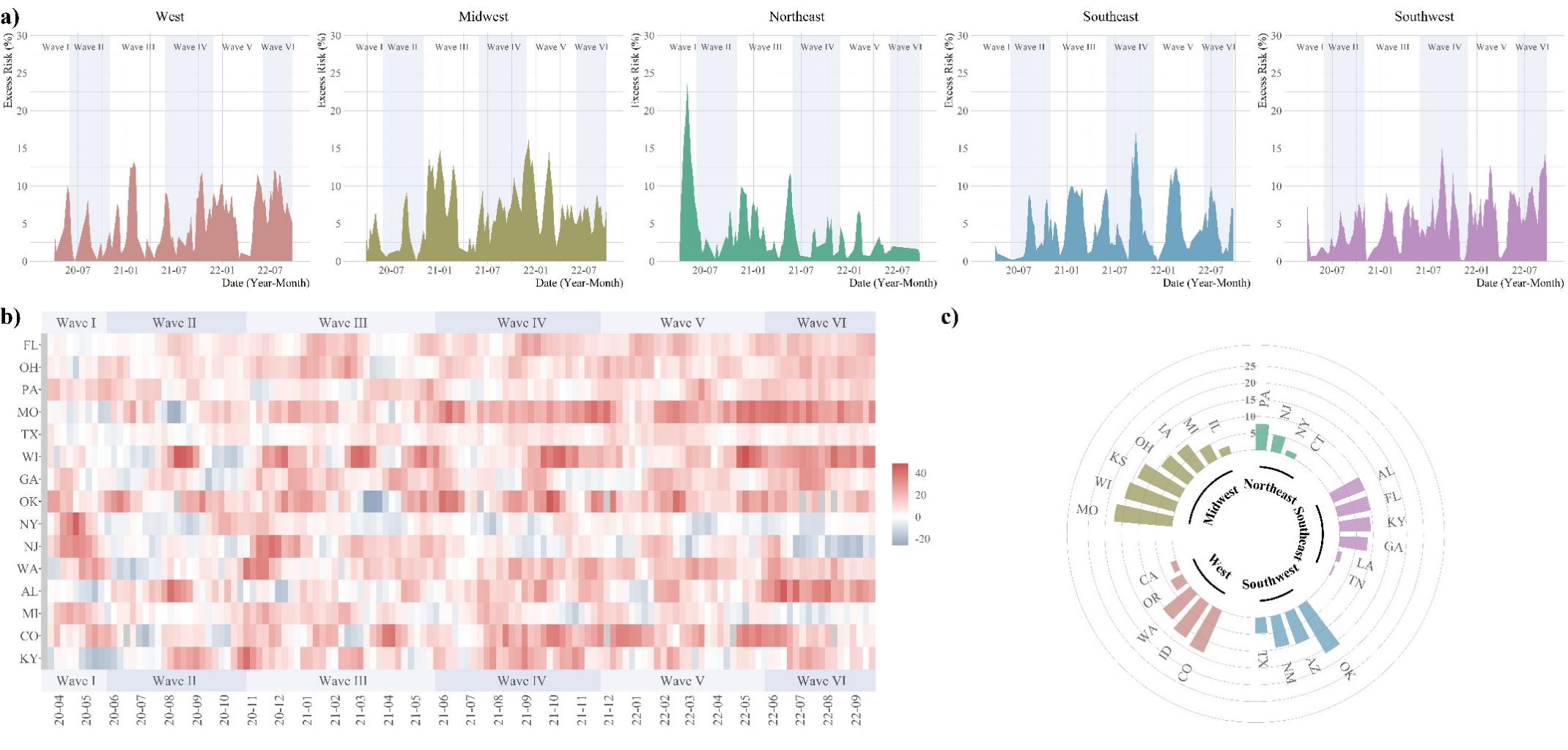
**Fig. S3 Temporal and spatial excess risks (%) associated with colorectal cancer in the United States** **(United States. 2020-2022)** A) temporal variated excess risks stratified by region; B) temporal variated excess risks stratified by state, where the 4-week moving average was operated for each time series; C) overall estimates by states (states with negative estimated values were not shown). The contributing cause of death was adopted.

#
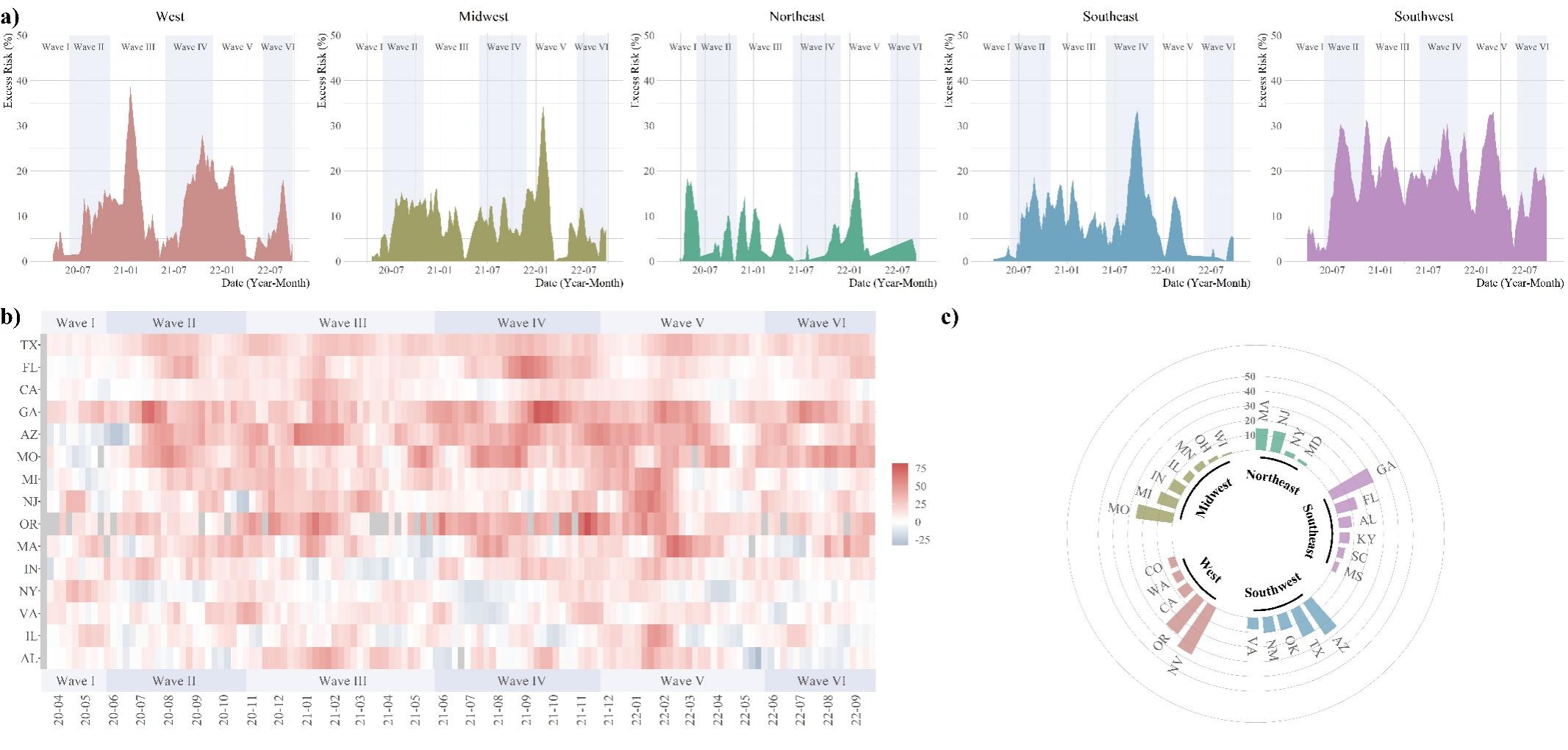
**Fig. S4 Temporal and spatial excess risk (%) associated with hepatic fibrosis/cirrhosis in the United States (United States. 2020-2022)** A) stratification by region; B) stratification by state, where the 4-week moving average was operated for each time series; C) overall estimates by states (states with negative estimated values were not shown). The contributing cause of death was adopted.

#
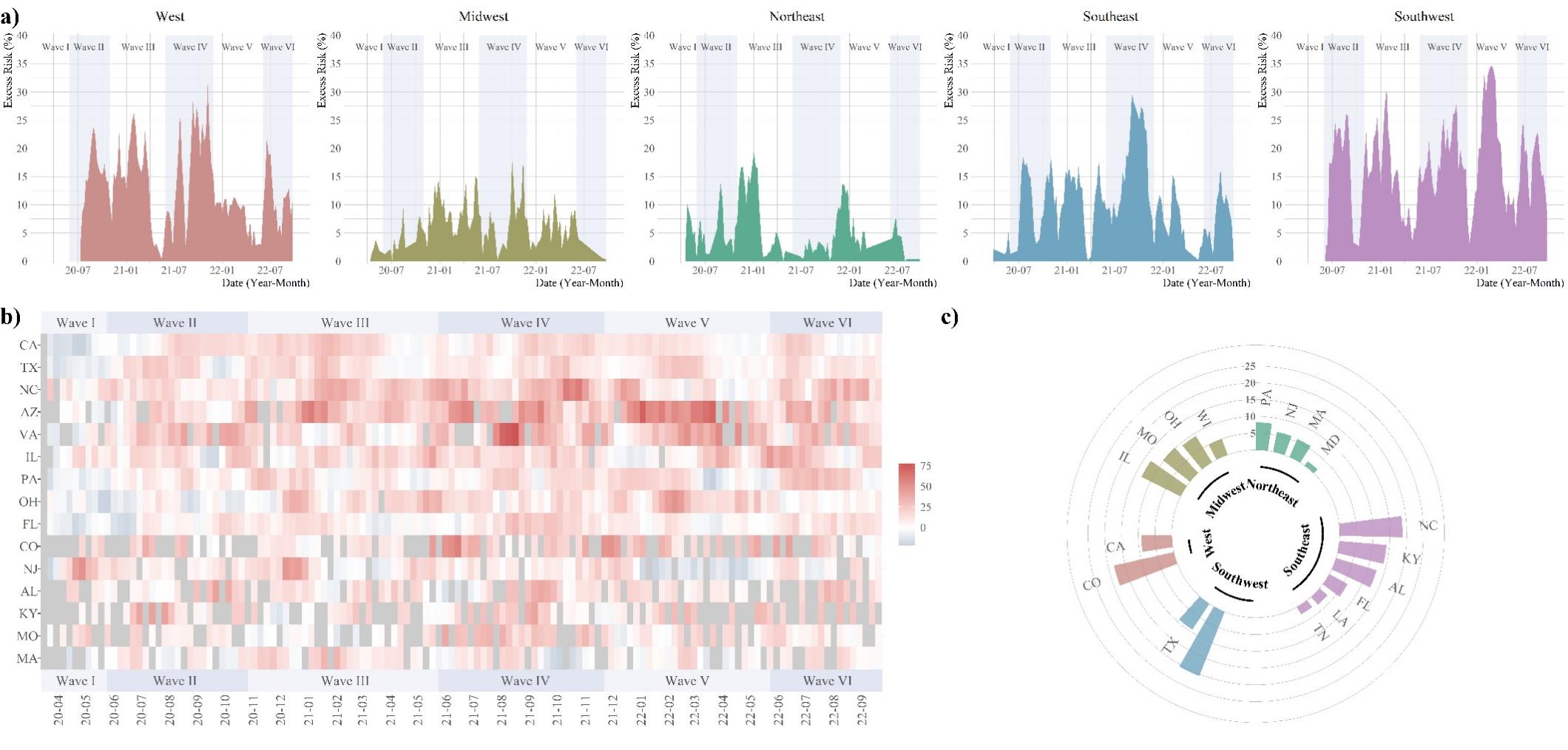
**Fig. S5 Temporal and spatial excess risks (%) associated with hepatic failure (United States. 2020-2022)** A) stratification by region; B) stratification by state, where the 4-week moving average was operated for each time series; C) overall estimates by states (states with negative estimated values were not shown). The contributing cause of death was adopted.
